# Understanding generational differences in digital skills and recreational behaviour for effective visitor management in forest destinations

**DOI:** 10.1038/s41598-025-02036-5

**Published:** 2025-05-23

**Authors:** Fruzsina Stefán, Mariusz Ciesielski, Andreas Weber, Kamil Choromański, Dariusz Gotlib, Karolina Taczanowska

**Affiliations:** 1 Institute of Landscape Development, Recreation and Conservation Planning / Department of Landscape, Water and Infrastructure, BOKU University, Vienna, Austria; 2 Department of Geomatics, Forest Research Institute, Sękocin Stary, Poland; 3 Faculty of Geodesy and Cartography, Warsaw University of Technology, Warsaw, Poland; 4 London Centre for Nanotechnology, University College London, London, England

**Keywords:** Digitalisation, Outdoor recreation, Visitor management, Forest, Digital society, Self-organizing maps (SOMs), Sustainability, Psychology and behaviour, Ecosystem services

## Abstract

Digitalisation is reshaping how people interact with natural environments, particularly in the context of outdoor recreation. This study examines generational differences in digital skills and recreational behaviour and their implications for visitor management in urban forests. Based on an online panel survey conducted in the Vienna metropolitan area (*n* = 3,121), we identified four distinct visitor profiles using Self-Organizing Maps: All-rounders (49.6%), Traditional Planners and Navigators (17.2%), Tech-Savvy Navigators (14.5%), and Balanced Adapters (18.7%). These clusters differ in both digital competence and used information sources used for planning and navigating forest visits. Significant relationships were found between generational groups and visitor profiles: older adults (Baby Boomers, Traditionalists) were featured more prominently among Traditional Planners, while younger generations (Generation Z, Millennials) were more prevalent in the Tech-Savvy group. Balanced Adapters included older individuals transitioning towards digital tool use. Further, our findings highlight that younger users reported less frequent forest visits than older cohorts. These insights underscore the need for inclusive, hybrid infrastructure and communication strategies that address varying recreational needs, including trip planning and navigation behaviours. This study contributes to forest management and nearby recreation planning by revealing a generational gap in technology use in digitally evolving societies.

**Supplementary Information:**

The online version contains supplementary material available at 10.1038/s41598-025-02036-5.

## Introduction

### Background

Forests play a crucial role in recreation and tourism, offering a diverse range of outdoor activities that contribute to both physical and mental well-being^1–3^. As key providers of cultural ecosystem services, forests support relaxation, exercise, and social interaction^4,5^. Recent trends, including greater health awareness, environmental concern, and post-pandemic behavioural shifts, have intensified the demand for nature-based experiences^6–10^.

Recreational activities range from intensive sports (e.g., running, cycling) to low-impact practices like walking, nature observation, and forest bathing^11–13^. These activities foster a deeper connection with nature, reduce stress, and enhance social cohesion, psycho-physical health, and overall well-being^1,14–18^. Recreational preferences often reflect age-related differences in visit frequency and activity choices^19,20^.

### Managing forest recreation in urban regions

The Vienna metropolitan area offers extensive green infrastructure with high recreational potential^21^. However, like many other urban areas, Vienna is experiencing increasing urbanisation and population growth, which have introduced several challenges for the provisioning and sustainable management of green spaces, including forests^21–23^. These pressures affect both ecosystems and local communities. Additional contributing factors, such as early retirement, increased car ownership, the shift to remote working post-COVID-19, rising health consciousness, and renewed interest in nature, are likely to further expand outdoor leisure activities. These continued growth trends place significant pressure on local environments^21,24,25^. Understanding recreational needs and behaviour in ecologically sensitive areas close to the city limits has become increasingly important^26,27^. Balancing public access and conservation has become a central challenge. Managers must adapt the infrastructure to respond to increased and diversified recreational demand, while promoting sustainable use^28–30^.

### Digitalisation of forest recreation management

The rapid development of technology and advancements in digitalisation are affecting human-environment systems are the subject of international interdisciplinary debate^31,32^. Digital tools increasingly act as interfaces between visitors and site management authorities^13,33–35^, transforming how activities are planned, navigated, and shared^36–38^.

These technologies offer benefits such as personalised recommendations, real-time updates, and community engagement^36^. For site managers, digitalisation facilitates sustainability and data-informed decision-making^39^. Tools such as GNSS-tracking, participatory GIS and visitor feedback platforms support behavioural insights, dynamic communication, and responsive planning^40,41^. Social media also plays a role in visitor outreach and education^31^ .

The rise of “datafication” and big data brings new potential in these scenarios and new opportunities for strategic planning and behavioural analysis^36^. However, digital datasets may not equally represent all societal groups or processes^42^. Literature highlights disparities, particularly regarding digital competence and its role in tourism and spatial planning^38^. Technical competencies are increasingly indispensable, particularly across generations.

### Digital skills across generations

Digital skills, the ability to operate hardware and software^43^, are essential in contemporary society, influencing many aspects of daily life, including outdoor recreation^44^. However, these competencies vary significantly across generations due to differences in access, education, and socio-economic background^45–48^. Understanding generational patterns in digital engagement is, therefore, critical for developing inclusive recreation strategies.

Each generation’s digital skills reflect the technological environment during their formative years (Hietajärvi 2024; Atasoy 2021^49^. Traditionalists (1900–1945) and Baby Boomers (1946–1964), who encountered digital technologies later in life, typically demonstrated lower proficiency. Generation X (1965–1980) adapted in adulthood, while Millennials (1981–1996) matured alongside the internet, becoming fluent digital communicators. Generation Z (1997–2010), raised in a hyper-connected world, often exhibit intuitive digital fluency and multitasking ability(Francis & Hoefel; Dexter 2018).

Although Gen Z shows high digital fluency, this does not always translate into interpersonal communication skills^50^. Some studies have noted weaker face-to-face communication^51–53^. However, their technological immersion does not appear to reduce their interest in nature or the time spent outdoors^54^.

In the context of this study, we refer to digital competence as participants’ self-assessed ability to effectively use digital technologies, not just access or frequency of use, but their perceived readiness and confidence to apply digital tools in everyday life, including recreational planning and navigation. This framing aligns with the evolving concept of the digital divide, which has shifted from a focus on acces (first-level) to include disparities in digital competence (second-level)^43,55^. While access to technology has broadly expanded, its effective use varies widely. Informal digital exposure alone is insufficient to develop comprehensive competence^53,56,57^. Digital skills are shaped by age, education, and socio-economic status, while gender differences appear minimal^46,58,59^.

These generational gaps influence how different groups engage with technology in outdoor settings. Younger generations tend to use digital tools more frequently for planning and navigating forest visits^36^, thereby increasing the demand for real-time digital infrastructure and personalised guidance^36,60^. In contrast, older generations often prefer printed maps and in-person information.

These differences call for adaptive management strategies that accommodate a range of preferences. Participatory Geographic Information Systems (PGIS) and Volunteered Geographic Information (VGI) offer opportunities to bridge digital and nature-based experiences while capturing valuable behavioural data^61,62^. Research on digital skill gaps, particularly in the tourism and leisure sectors^56^, stress the growing demand for digital skills across all population groups. Recreation planners can use these insights to design more inclusive, generation-sensitive infrastructure and programs. Understanding visitor behaviour, particularly generational needs and information preferences, is key to developing adaptive strategies. Understanding visitor behaviour, especially generational needs and information preferences, is key to developing adaptive, inclusive strategies.

**Fig. 1:**
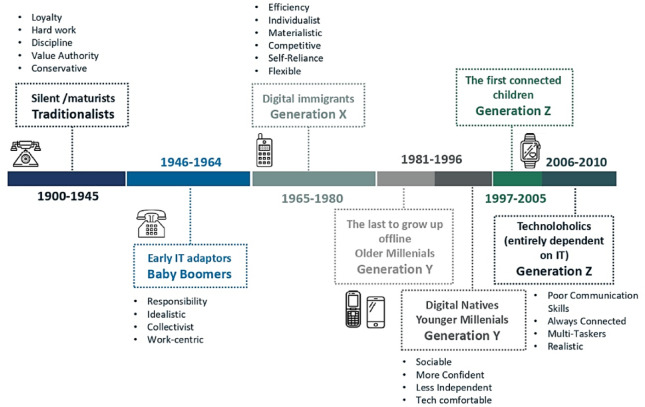
A comparative timeline of generations. After Seyfi et al. (2024)^63^; Francis, Hoefel (2018)^60^; Neilson (2021)^64^; Deloitte (2020)^65^. Created by the authors. The generational classifications reflect birth year ranges and the dominant digital environments during formative years. These distinctions inform our interpretation of digital skill levels and technology adoption in forest recreation.

### Study objectives

Our study aims to address the intersection of digital skills, generational differences, and outdoor recreational behaviour, with a focus on forests. Specifically, we examine how digital competence influences the use of digital tools for planning and engaging in forest visits across generational groups.

Focusing on recreational use in the forests within the Vienna Metropolitan Area, this study investigates how generational differences reflect broader patterns of digital technology adoption in outdoor recreation. We analyse visitor preferences for digital versus traditional information sources to understand generational trends in trip planning and on-site navigation. The findings aim to clarify how digital habits shape forest use and visitor engagement. Our specific research questions are as follows:

1. What information sources are used for planning and navigating forest visits in the Vienna Metropolitan area?
2. How do visitors assess their digital skills?
3. What visitor profiles emerge based on digital skills and the preferred information sources used for planning forest visits and navigation during forest trips?
4. Do these visitor profiles differ across generations?
5. Do perceived digital skills differ across generations?
6. Does forest visitation differ by generation and digital skill level?

## Methodology

### Study area

This study is based on empirical data collected from residents of the Vienna metropolitan area in Austria. Vienna is known for its extensive green spaces, including urban forests, parks, and recreational areas, which are easily accessible to residents and visitors^21,66^. This blend of urban and natural environments makes Vienna an ideal setting to investigate how different generations use digital tools to enhance their outdoor recreation experiences.

Vienna’s progressive approach to urban planning and sustainability further underlines the relevance of this study. The city has long been a leader in integrating green infrastructure into urban development, serving as a model for other metropolitan regions^67^. In addition, Vienna’s commitment to digital innovation and smart city initiatives provides a strong foundation for exploring how digital competence can support inclusive and adaptive urban forest management^68^.

**Fig. 2:**
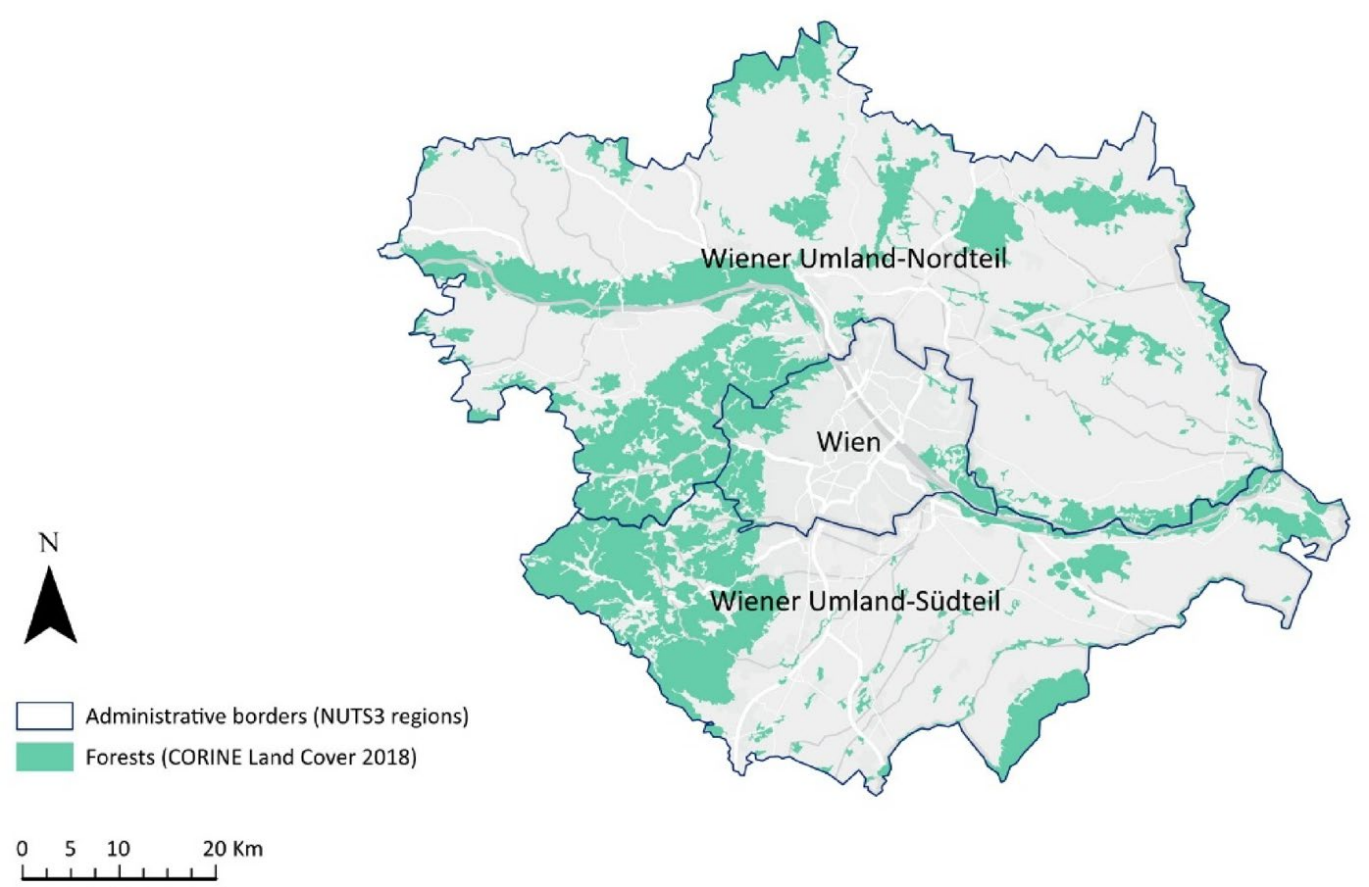
Forest areas in the Vienna metropolitan region, based on the CORINE Land Cover 2018 and NUTS3 administrative boundaries. Map created by the authors using ArcGIS Pro (version 3.1.0; ESRI, Redlands, CA, USA; https://www.esri.com/arcgis).

Forests are significant for urban residents because they provide accessible natural spaces for recreation and relaxation^69^. Figure 2 shows the locations of the forest areas within the study area. The Vienna metropolitan area functions as an integrated urban region where both intra-city forests (urban forests) and those near the urban fringe (peri-urban forests) are used collectively as a continuous recreational landscape^70,71^. Studies show that proximity to green spaces is a key determinant of recreational use, with urban residents frequently visiting nearby forests regardless of administrative classification^72,73^.

### Survey design and data collection

#### Online panel survey details

An online survey was conducted via the Maptionnaire platform with a representative sample of residents in autumn 2023 (*n* = 3,121). Participants were recruited through a commercial online panel (Marketagent), and quotas were applied to reflect the population characteristics of the Vienna metropolitan area based on gender, age, and regional distribution (Section “*Sample description (n = 3*,*121)*”, Table 1). Each participant was given a unique ID linked to the date and time at which they completed the questionnaire, enabling the linkage of all answers to individual users. Responses remained anonymous (no personal data or IP addresses of the respondents were stored). The survey was accessible to respondents in a browser via a smartphone, tablet, or computer.

The Supplementary Information accompanying this study is organised into four documents (S1–S4), which provide additional details on the methodology and results: S1 includes the full survey instrument and regional population data used for sample design; S2 presents generational trends in forest visitation and digital tool usage before, during, and after forest visits; S3 summarises patterns of digital skills and tool use across Self-Organizing Map (SOM) clusters; S4 contains the hot spot analysis of forest recreation starting points by generation and digital skills, along with statistical analyses of forest visit frequency and group differences.

The study design was approved by the Commission on the Ethics of Research Involving Human Subjects at the Forest Research Institute (IBL) in Poland and was performed following relevant guidelines and regulations. Informed consent was obtained from all participants and their legal guardians using an online panel provider.

#### Sample description (*n* = 3,121)

A representative sample reflecting the population’s characteristics was selected using the quota method. The online survey aimed to capture the social demographics of Vienna and its surrounding communities, ensuring diverse representations across gender, age, and regional distribution.

**Table 1:** Comparison of sample composition (*n* = 3,121) and population distribution based on census data (Statistik Austria, 2023).

	Population composition		Sample composition	
Population characteristics	*N*	Percentage	*n*	Percentage
Total	2,679,090	100%	3,121	100%
Age group:				
< 16 years	Not included in this study	Not included in this study	Not included in this study	Not included in this study
16–18 years	78,586	3.4%*	56	1.8%
19–30 years	432,609	18.1%*	612	19.6%
31–40 years	405,968	17.0%*	562	18.0%
41–50 years	362,497	15.8%*	534	17.1%
51–65 years	545,562	25.1%*	865	27.7%
Older than 65 years	437,702	20.6%*	615	19.7%
Gender:				
Female	1,369,813	51.1%	1,585	50.8%
Male	1,309,277	48.9%	1,526	48.9%
Diverse	No data	No data	9	0.3%
Regions:				
R 1.1: Vienna Centre	331,911	12.4%	371	11.9%
R 1.2: Vienna Centre East	193,959	7.2%	194	6.2%
R 1.3: Vienna South	545,616	20.4%	886	28.4%
R 1.4: Vienna West	514,058	19.2%	565	18.1%
R 1.5: Vienna Northeast	396,553	14.8%	247	7.9%
R 2.1: Wiener Umland/Nordteil (NUTS3)	342,995	12.8%	375	(12%)
R 2.2 – Wiener Umland/Südteil (NUTS3)	353,998	13.2%	484	(15.5%)

*cumulative percentage refers to population ≥ 16 years (*N* = 2,262,924).

The sample consisted of 50.8% female, 48.9% male, and 0.3% diverse respondents. Participation was open to respondents aged 16 years or older. A total of 1.8% of the participants were 16–18 years of age, 19.6% were 19–30 years of age, 18.0% were between 31 and 40 years of age, 17.1% were between 41 and 50 years of age, 27.7% were between 51 and 65 years of age, and 19.7% were over 65 years of age (Table 1).

Regional quotas aligned with the Viennese district layout and surrounding NUTS3 areas. The coefficient of determination between respondent count and population data was 0.72 for grid squares and 0.95 for districts. Nine regional clusters were used, corresponding to the administrative and spatial distinctions shown in Fig. 2.: R1.1 Wien Innen = Vienna Centre (11.9%); R1.2 Wien Innen Ost = Vienna Centre East (6.2%); R1.3 Wien Süd = Vienna South (28.4%); R1.4 Wien West = Vienna West (18.1%); R1.5 Wien Nordost = Vienna Northeast (7.9%); R2.1 Wien Umland-Nordteil-Wien = Vienna Surrounding Communities North (12%); R2.2 Wien Umland-Südteil-Wien = Vienna Surrounding Communities South (15.5%). Participation in the survey was voluntary, and participants were informed about the study’s purpose and procedures. No personal information, such as home addresses, was collected. Participants younger than 16 years were excluded from the study.

Further methodological details, including the full sampling framework, are presented in Supplementary Information S1 (Tables S1–S2).

### Survey content

The questionnaire presented in this study was designed ad hoc to investigate citizens’ perspectives on outdoor recreation in urban forests in the Vienna metropolitan area. The survey was conducted via the Maptionnaire application, an online PPGIS tool that allows participants to map their answers with an intuitive interface. The flexibility of the questionnaire supports various question types and interactions, including mapping, drawing, evaluating, and selecting, making it suitable for public participation in urban planning^61,74^. The online standardised questionnaire aimed to capture sociodemographic characteristics, psychographic data, and digital behaviour patterns related to urban forest areas in Vienna. It included both closed- and open-ended questions for the quantitative analysis of categorical data and qualitative insights. The respondents provided detailed information on their outdoor activities and frequency of forest visits, and used outdoor planning and navigation tools.

The respondents provided detailed insights via digital maps, providing information on their outdoor activities, frequency of visits to forest areas, associated values, and barriers faced. A significant component of the analysis involves the examination of “digital traces” left by visitors. This assessment of the digital footprint helps unravel the intricacies of recreational behaviour and its implications for planning and management. Geographic data points (e.g., locations of forest starting points) were collected along with the corresponding attributes (e.g., visitation frequencies).

The survey collected data on demographics, forest visitation frequency and duration, activity types (e.g. walking, jogging, nature observation), motivations (e.g. relaxation, fitness), and digital tool usage (e.g. smartphones, GPS, apps) before, during, and after forest visits. It also captured navigation sources (e.g. digital maps, printed maps, local knowledge) and usage functions (e.g. planning, tracking, sharing).

It also captured respondents’ self-assessed digital skills^75^ and usage patterns of various digital tools used in the outdoor recreation context. Self-assessment instruments are a type of assessment instrument that involves individuals subjectively assessing their skill level by responding to a set of questions. Using self-assessments to determine digital skills offers several advantages. They are resource-efficient, applicable to large samples, and useful for assessing learning and its progression^75,76^. A full list of survey items relevant to the presented results is provided in Supplementary Table S1 (Supplementary Information S1).

### Data analysis

Survey data were exported as comma-separated values (CSV) files and imported into SPSS for cleaning and analysis. Selected variables were recoded to facilitate interpretation. Data were analysed to identify patterns across generations, digital skills, and tool usage behaviours.

#### Categorization of generational differences via established frameworks

The recreational behaviours of different age groups were systematically categorized into distinct generations via established frameworks from Francis & Hoefel (2018)^60^, Neilson (2010)^64^ and Deloitte (2020)^65^ (Fig. 1). This approach enables a nuanced examination of recreational behaviours aligned with specific generational characteristics^63^. Based on this framework, age ranges were recorded in the dataset into generational categories. This categorisation method facilitates a detailed analysis of how each generation engages with digital tools and outdoor recreational activities, ensuring a comprehensive understanding of the generational differences in recreational patterns.

#### Statistical analysis

The survey data were analysed using statistical software tools, IBM SPSS Statistics (version 29.0) and R (version 4.3.1; https://www.r-project.org/) via RStudio. Descriptive statistics were employed for the initial data exploration to summarise the main features of the dataset. Chi-square tests, Fisher’s exact tests, and nonparametric tests were performed in R to investigate differences between age groups and examine relationships between variables^77^.

To recognise categories that differ most, we included mosaic plots using residual-based shading (Pearson residuals). These were generated in R (v4.3.1) by the first author (Stefan) using the vcd package, with data handling supported by haven and dplyr. The colours represent the level of the residual for that cell/combination of levels. The legend is always presented at the plot’s right. Blue shading means there are more observations in that cell than would be expected under the null model (independence). Red means there are fewer observations than would have been expected. Grey cells indicate that the corresponding Pearson residual is within a 95% confidence interval around zero (independence). The mosaic plots visually represent the key trends related to the relationships between different categorical variables, providing insights into how various factors, such as age groups, digital tool usage, and information source preferences, interact with each other and help to identify patterns and interactions.

Furthermore, a hotspot analysis using the Getis-Ord Gi* statistic was employed to identify statistically significant clusters of high and low values (hot and cold spots) in the spatial data and provide insights into the forest utilization patterns This method is widely used in geographic and environmental research for detecting spatial patterns of activity^78–80^. In this study, it was applied to identify clusters of forest visit starting points across generations and digital competence levels, highlighting areas of concentrated recreational use and digital engagement. Using Getis-Ord Gi* tool in ArcGIS Pro (version 3.1.0; ESRI, Redlands, CA, USA; https://www.esri.com/arcgis), nine distinct maps were generated (Supplementary Information S4), revealing significant clusters of forest visitation starting points within a 500–1000-meter distance band. This range was chosen to balance the identification of localized clusters with broader regional trends, thereby ensuring the detection of statistically significant hot spots without losing the granularity required to understand specific patterns of recreational behaviour.

The results are presented in Supplementary Information S4 (Figures S46–S54 and Tables S5, S6). Additional supporting data, including statistical outputs and visualisations, are available in Supplementary Information S2–S4.

#### Self-Organizing maps (SOM) methodology

In this study, a type of artificial neural network known as the Self-Organizing Maps (SOM)^81,82^ was employed to explore visitor characteristics^83^. SOMs are a type of artificial neural network used for clustering and visualising high-dimensional data^81,82,84,85^.

Since its introduction by Teuvo Kohonen in 1982^81^, the SOM and other unsupervised learning algorithms have been widely and effectively used across various scientific fields, including tourism research^86,87^.

SOMs are competitive, unsupervised neural networks that transform high-dimensional data into a two-dimensional map, preserving the topological relationships of the data^81,88,89^. This method is particularly beneficial for high-dimensional and non-linear data structures and is effective for classifying relationships between variables and observing data patterns over time^84^. Unlike standard clustering techniques, SOM ensures that similar data points remain spatially close in the two-dimensional representation^88,90^. This means that the relationships between respondents’ navigation habits, digital competence, and generational attributes can be visualised intuitively.

Unlike traditional methods such as PCA or k-means clustering, which assume linear separability in data, SOM effectively maps complex, non-linear patterns by dynamically adjusting the structure of the map to reflect underlying data distributions^91,92^. Instead of reducing data to principal components (as in PCA), SOM retains the richness of the input variables while projecting them into an interpretable 2D grid, making it a powerful tool for detecting hidden clusters and relationships^93^.

The SOM analysis can provide fresh insights into visitor profiling challenges. One of the key strengths of SOM in this study is its ability to group respondents based on shared characteristics, which enabled us to identify generational trends in digital tool adoption, helping to determine which users are more inclined towards technology-driven navigation and which respondents rely on traditional knowledge sources. We could uncover attitudinal and behavioural clusters that extend beyond age groups, revealing how different cohorts engage with technology for forest visits and visualise behavioural shifts through the clustered 2D projection, offering insights into how digital competence and tool use evolve across groups.

The SOM analysis followed the standard four-phase learning approach^89^:

1. *Initialisation*: The algorithm starts by assigning random weight vectors to each neuron in the SOM grid.
2. *Competitive Learning*: For each input (respondent), the algorithm identifies the Best Matching Unit (BMU)—the neuron whose weight vector is closest to the input vector (based on Euclidean distance).
3. *Neighbourhood Updating*: Once the BMU is found, its weight vector and those of its neighbouring neurons are adjusted to better represent the input data, ensuring that topologically similar respondents remain close together.
4. *Convergence*: This iterative process continues until the map stabilises, forming meaningful clusters of visitor behaviour.

For this analysis, we applied the SOM algorithm using the R Kohonen package, configuring a hexagonal toroidal grid^90,94^.This configuration was chosen to eliminate edge effects, ensuring that all neurons have equal connectivity and neighbours on all sides. All SOM visualisations were created in R (version 4.3.1; https://www.r-project.org/).

**Fig. 3:**
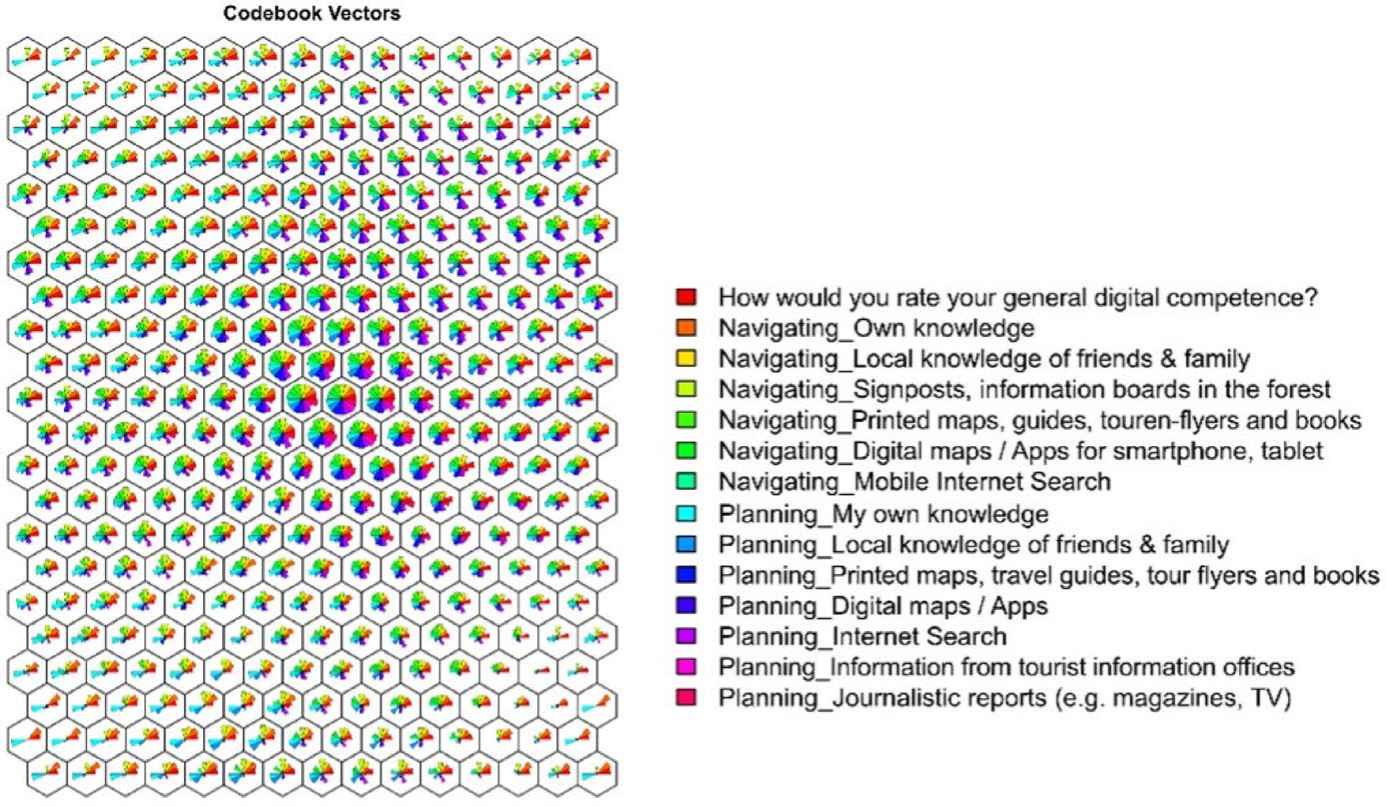
Fan diagram showing the spatial distribution of the codebook vectors in two dimensions (2D). Each node represents the prototype position of one of nine variables related to digital competence, navigation behaviour, and planning preferences for forest visits. Colour-coded vectors indicate the relative association of each node with individual variables.

The SOM was configured with a 15 × 22 grid, resulting in a map size of 330 units. Determining the optimal SOM size involves calculating the appropriate grid dimensions to ensure that the map is large enough to capture the complexity of the data while still being computationally feasible^88^. A commonly used heuristic suggests setting the map size to approximately 5 times the square root of the number of samples in the dataset^84^. Following this approach, the configuration accommodated the full dataset, which included 3,121 respondents with 14 variables related to the use of digital and analog tools for forest recreation (Fig. 3).

The SOM was initialized via principal component analysis (PCA). The learning ratio (alpha), which determines the magnitude of weight changes, started at 0.03 and gradually decreased to 0.01. The training process was repeated over multiple iterations (rlen = 12,000) to account for random variations in start and input order, resulting in multiple SOMs.

The SOM process involves constructing a neural network with a Gaussian neighbourhood function initialised with values from the input data matrix. The training process used sequential training, where each neuron in the grid competed to win each input vector based on the Euclidean distance between the neuron’s weight vector and the input vector. The weights of the winning neuron and its neighbouring neurons are adjusted to resemble the input vector more closely, influenced by the Gaussian neighbourhood function.

**Fig. 4:**
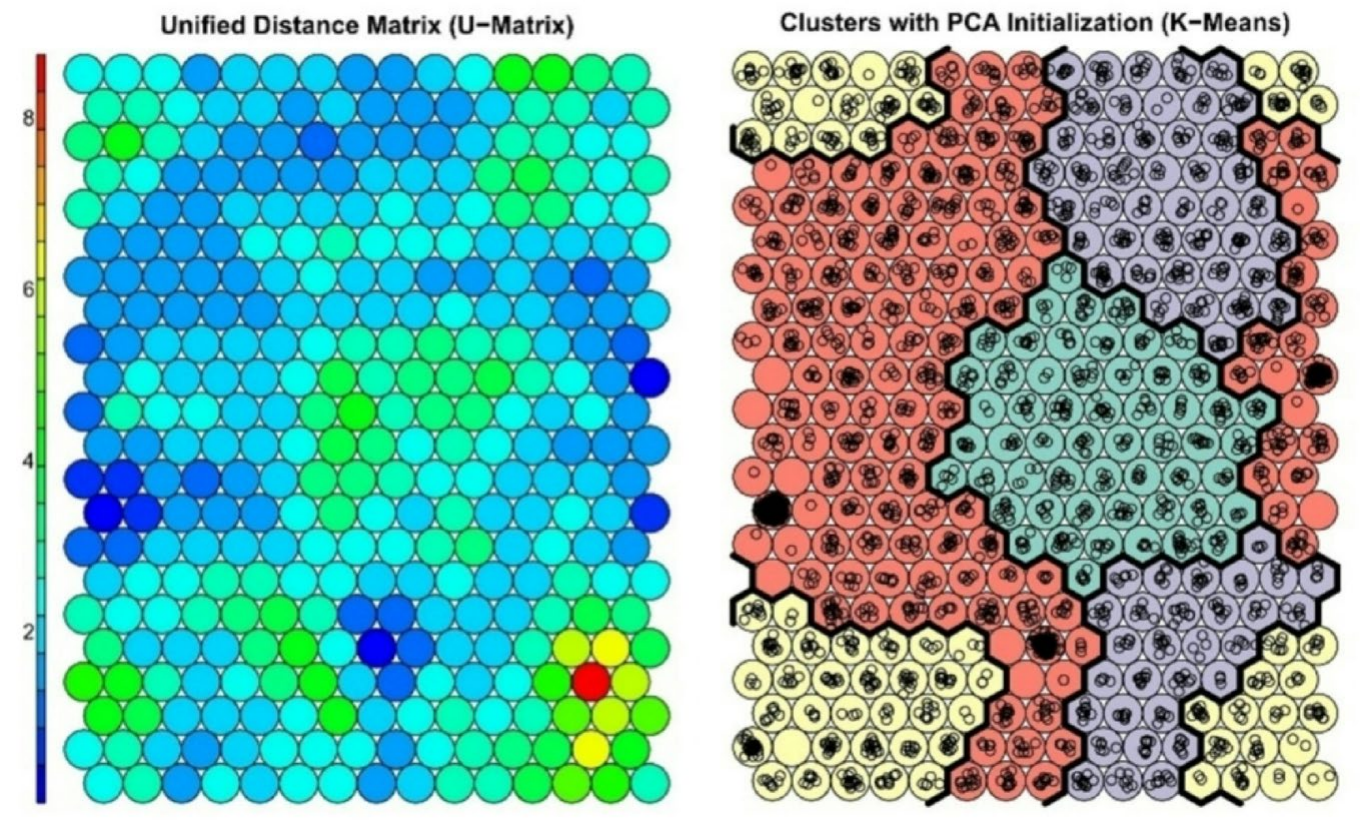
Visualisation of the Self-Organizing Map (SOM) output: the Unified Distance Matrix (U-matrix, left) and K-means clustering with PCA initialisation (right). The U-matrix represents the Euclidean distances between neighbouring neurons, indicating similarity or dissimilarity in the respondents’ mapped profiles. Blue and green shades show areas where neighbouring nodes are similar, suggesting that respondents mapped there share comparable characteristics. In contrast, the yellow, orange, and red areas reflect greater distances, representing more distinct respondent profiles. The right panel shows the clustering results derived from K-means clustering applied to the SOM, with PCA used for initialisation. Each cluster is represented by a unique colour, while black boundaries highlight the separations between clusters (Cluster 1 = yellow, Cluster 2 = red, Cluster 3 = purple, Cluster 4 = green). This clustering illustrates how respondents with similar behavioural and digital characteristics group together while more distinct profiles are mapped into separate clusters. The PCA initialisation enhances interpretability by improving group separation, offering clear insights into the distribution and structure of behavioural patterns across the respondent groups.

Both the quantization error and the topographic error were monitored to ensure accuracy and topological preservation. The mean quantization error represents the average distance between data points and their corresponding best-matching unit (BMU). The SOM was run multiple times to minimise quantisation errors and to select the optimal SOM. The lowest quantisation error achieved was 3.5, indicating a strong accuracy in mapping the input data. The topographic error, which measures the preservation of the topological structure, ensured that the spatial relationships of the data were maintained throughout the analysis. The SOM clustering was optimised via k-means clustering on the trained SOM model. These visualisations display how different neurons (representing clusters of respondents) are associated with the frequency of digital tool usage. High values in certain areas indicate that respondents within those clusters use digital tools frequently. The SOM analysis revealed four distinct clusters, each representing groups of respondents with similar digital competence and tool usage patterns (Fig. 4).

Cluster 1 (yellow) represents the largest group in the dataset and is primarily located at the edges of the SOM map, indicating a widespread and diverse cluster. Cluster 2 (red) is concentrated in the central and top-left areas of the map. The central positioning indicates a strong presence in the data. Cluster 3 (purple) occupies the right-central part of the map and is clearly defined, suggesting a more specialised subset of respondents. Finally, Cluster 4 (green) is located in the centre region of the SOM, forming a well-separated cluster that, while slightly smaller than Cluster 3, remains well-structured and distinct (Fig. 4).

Once the clusters were identified, each respondent in the dataset was mapped to one of these four clusters based on the SOM analysis. The final dataset, containing the respondent IDs along with their corresponding cluster allocations and cluster assignments of the variables, including generations, was saved to a csv file. Additionally, a breakdown of the number and percentage of respondents in each cluster was calculated and exported for reporting.

## Results

### Digital skills and information sources used for planning and navigation during forest visits

#### Overview of digital skills and information sources

**Table 2:** Frequency of use of different information sources for planning and navigating outdoor forest visits. Values indicate how often each source was used, from “very often/always” to “never”, based on respondents’ self-reported usage.

	Number of Respondents	Very often/always (%)	Often (%)	Sometimes (%)	Rarely (%)	Never (%)
**Information Source for Planning**						
Own knowledge	2,362	1,034 (43.8)	703 (29.8)	395 (16.7)	124 (5.2)	106 (4.5)
Local knowledge of friends, family, etc.	2,359	191 (8.1)	563 (23.9)	920 (39.0)	470 (19.9)	215 (9.1)
Printed maps, travel guides, tour flyers and books	2,351	67 (2.8)	178 (7.6)	435 (18.5)	676 (28.8)	995 (42.3)
Digital maps for PC / Apps for smartphone, tablet, and smartwatch	2,360	210 (8.9)	434 (18.4)	647 (27.4)	494 (20.9)	575 (24.4)
Internet Search	2,356	245 (10.4)	507 (21.5)	732 (31.1)	446 (18.9)	426 (18.1)
Information from tourist information offices (not via the internet)	2,325	59 (2.5)	93 (4.0)	220 (9.5)	428 (18.4)	1,525 (65.6)
Journalistic reports (e.g., magazines, TV)	2,333	51 (2.2)	106 (4.5)	461 (19.8)	665 (28.5)	1,050 (45.0)
**Information Source for Navigation**						
Own knowledge	2,311	978 (42.3)	830 (35.9)	377 (16.3)	93 (4.0)	33 (1.4)
Local knowledge of friends, family, etc.	2,274	170 (7.5)	512 (22.5)	832 (36.6)	460 (20.2)	300 (13.2)
Signposts and information boards in the forest	2,293	706 (30.8)	818 (35.7)	557 (24.3)	127 (5.5)	85 (3.7)
Printed maps, guides, tour flyers, and books	2,285	97 (4.2)	210 (9.2)	496 (21.7)	615 (26.9)	867 (37.9)
Digital maps / Apps for smartphone, tablet, and smartwatch	2,289	283 (12.4)	502 (21.9)	657 (28.7)	415 (18.1)	432 (18.9)
Mobile Internet Search	2,274	190 (8.4)	434 (19.1)	674 (29.6)	457 (20.1)	519 (22.8)
A GPS device (not GPS via smartphone)	2,262	103 (4.6)	192 (8.5)	296 (13.1)	276 (12.2)	1,395 (61.7)
Printouts from the Internet, digital maps	2,246	61 (2.7)	129 (5.7)	382 (17.0)	404 (18.0)	1,270 (56.5)

Table 2 presents the frequency of information source use for planning and navigation during forest visits. The PPGIS survey reveals distinct preferences in how visitors plan and navigate trips. Personal experience (43.8%) and local knowledge (8.1%) are the most frequently used sources during planning, while printed materials (42.3% never) and tourist information offices (65.6% never) are rarely consulted.

Digital tools play a more prominent role during navigation than during planning. While only 8.9% use digital maps regularly for planning, 12.4% use them frequently for on-site navigation. Visitors rely heavily on signposts (30.8%) and personal memory (42.3%) while navigating, with standalone GPS devices (61.7% never) and printed digital maps (56.5% never) showing low relevance.

These findings point to a strong reliance on experiential knowledge and on-site cues, with digital tools supplementing rather than replacing traditional strategies, particularly in real-time decision-making during forest visits.

#### Visitor profiles

In this study, the Self-Organizing Map (SOM) analysis identified four distinct visitor clusters based on self-reported digital skills and tool usage for planning and navigation in the context of forest recreation (Figs. 4, 5 and 6; Supplementary Figures S32–S45; Table S4 in Supplementary Information S3).

*Cluster 1*,* “All-rounders”* (49.6%), represents the largest and most heterogeneous group. These visitors exhibited moderate to high digital competence (Figure S45) and used a mix of digital and traditional tools. While they frequently consult internet-based sources during the planning phase, such as general web searches and online information platforms (Figures S35, S36), they rely less on digital tools during navigation. Instead, they preferred more familiar, analogue-based methods, including personal memory and local knowledge (Figures S32, S33, S39, S40), printed maps (Figures S34, S42), and verbal recommendations from friends or family. Their relatively low use of mobile internet, navigation apps, and digital maps on-site (Figures S43, S44) suggests a flexible but experience-driven approach to forest recreation.

*Cluster 2*,* “Traditional Planners and Navigators”* (17.2%) is characterised by users with moderate digital competence (Figure S45), who rely predominantly on their knowledge and prior experience for both planning and navigation (S32, S39). This group has low reliance on digital information sources, internet searches and mobile apps (Figures S35, S36). Likewise, reliance on physical signposts during navigation is lower compared to other clusters (Figures S41–S44), suggesting a high degree of familiarity with their destinations. Overall, their behaviour reflects a routine-based and self-reliant approach, shaped by habitual visitation patterns and a preference for autonomous, low-tech decision-making.

*Cluster 3*,* “Tech-Savvy Navigators”* (14.5%) consists of digitally confident users. They report the highest levels of digital competence (Figure S45) and rely primarily on smartphones, GPS, and online platforms for both trip planning (Figures S35, S36) and real-time navigation (Figures S41–S44). Engagement with printed materials (Figure S34), tourist offices (Figure S38), and social knowledge sources such as friends, family, or personal memory (Figures S32, S37, S39; Table S4) is minimal, reflecting a fully digital and independent navigation style.

*Cluster 4*,* “Balanced Adapters”* (18.7%), demonstrates a mixed-use strategy. This group demonstrated moderate digital skills (Figure S45) and combined digital planning (Figures S35-S36) with continued use of printed resources and personal recommendations (Figures S33, S34, S37). They frequently use mobile internet during navigation (Figure S44) but remain open to traditional tools as backups. This group exemplifies situational adaptability, switching between digital and analog tools depending on context, familiarity, and availability.

These clusters reflect nuanced differences in how visitors engage with digital and traditional tools. They underscore the importance of a flexible, multi-modal infrastructure to support various recreational behaviors and user preferences in forest environments.

**Fig. 5:**
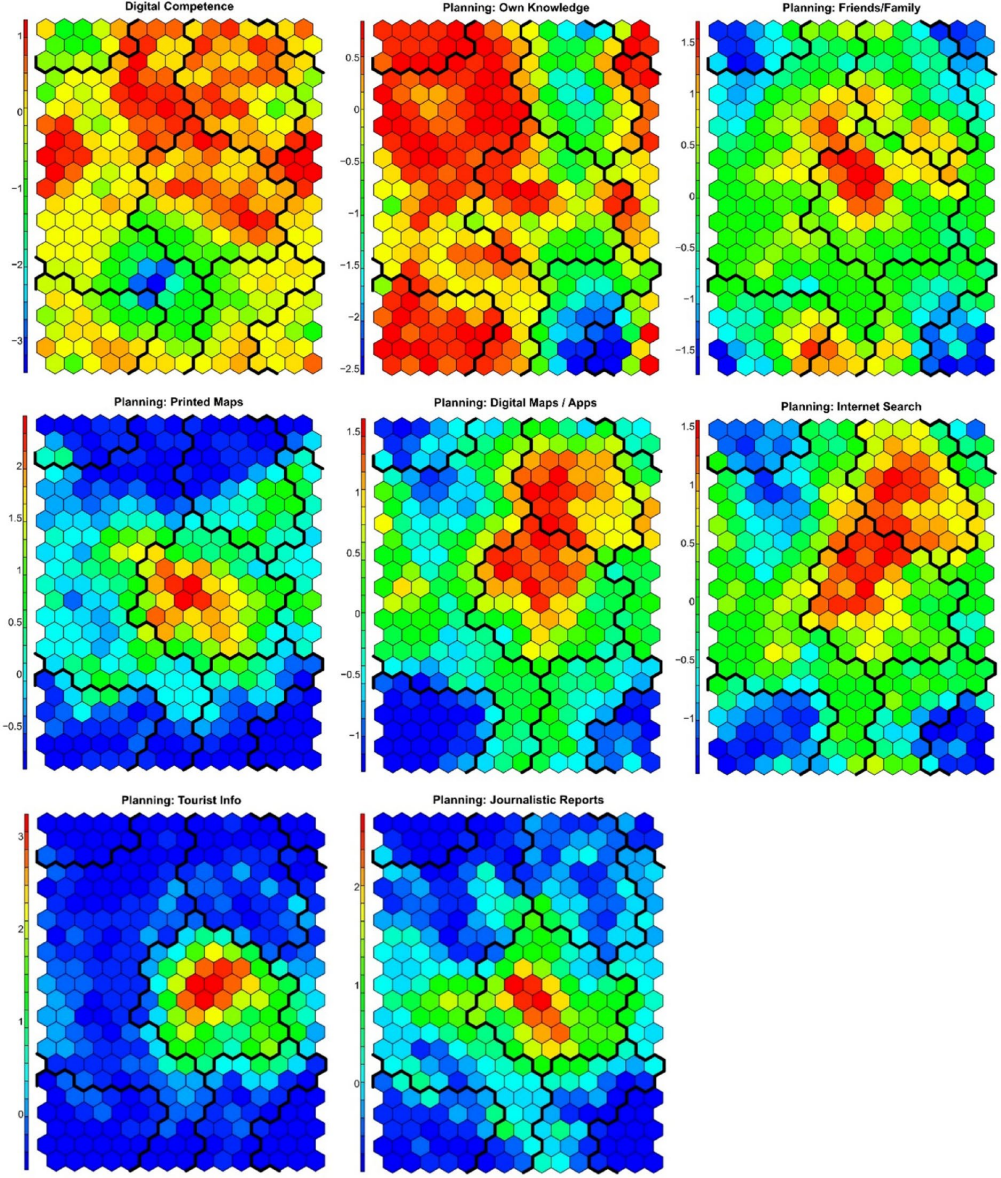
Component planes showing the distribution of self-reported digital competence and reliance on different information sources used before forest visits. Variables included: personal knowledge; local knowledge from friends or family; printed maps, tour flyers, and guides; digital maps and apps for smartphones, tablets, and smartwatches; internet search; tourist information offices (excluding online); and journalistic reports (e.g. magazines, TV). Warmer tones (red, orange) indicate higher reliance on a given source; cooler tones (blue, green) indicate lower reliance.

**Fig. 6:**
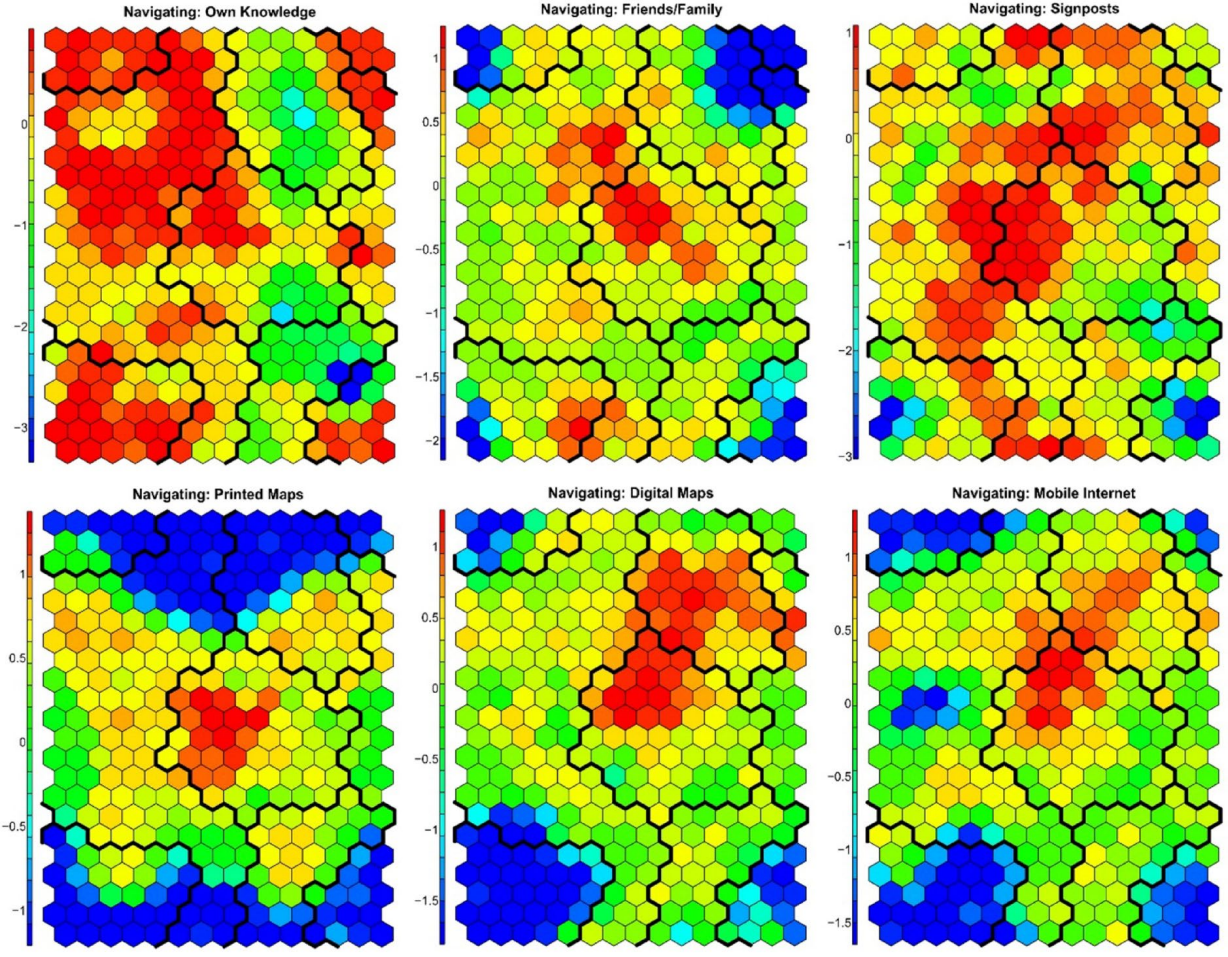
Component planes showing the distribution of self-reported reliance on navigation tools during forest visits. Variables included: personal knowledge; local knowledge from friends or family; signposts and information boards in the forest; printed maps, guides, and tour flyers; digital maps or apps for smartphones, tablets, and smartwatches; and mobile internet search. Warmer colours (red, orange) indicate higher reliance on each navigation source, while cooler tones (blue, green) indicate lower reliance.

### Generational differences

#### Relationship between visitor profiles and generational cohorts

**Fig. 7:**
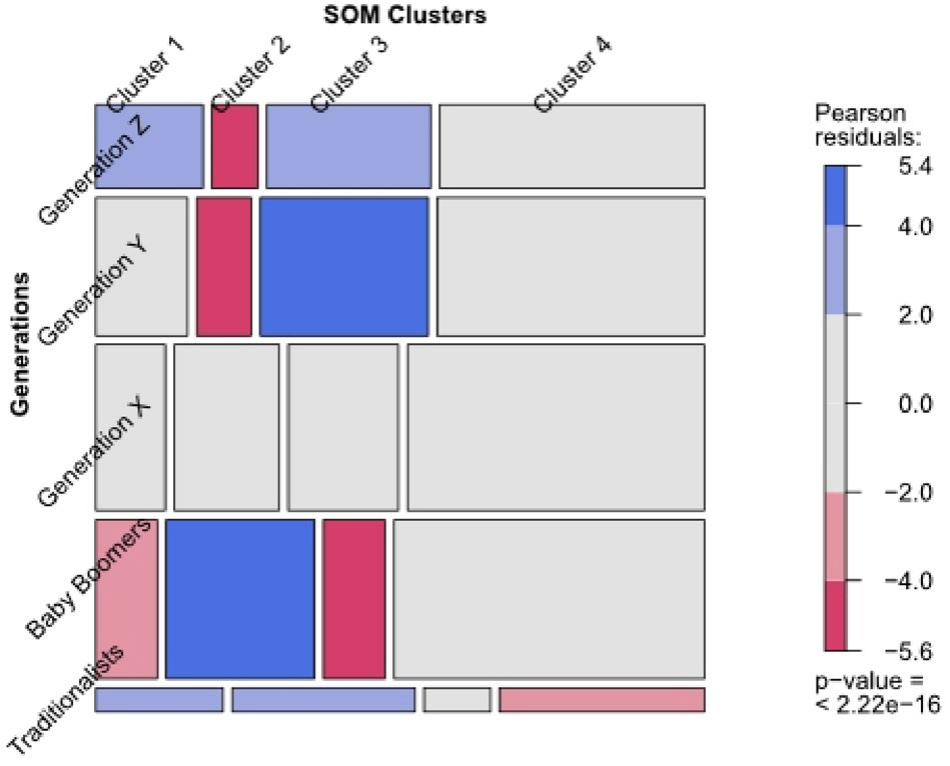
Mosaic plot of the generational distribution across SOM clusters (*n* = 2,507). Blue areas indicate overrepresentation of a generation within a given cluster, while red areas indicate underrepresentation. The colour gradient reflects the Pearson residuals, with darker tones denoting a greater deviation from the expected frequencies under the assumption of independence.

To explore age-based patterns in recreational behaviour, each survey respondent was assigned to one of the four SOM clusters and matched with their generational cohort (Table 3; Supplementary Information S3). A Chi-square test confirmed a strong and statistically significant association between generation and cluster assignment (Χ²(12) = 181.05, *p* < 2.2e-16), indicating that generational identity plays a substantial role in shaping the use of digital tools and planning strategies. The generational distribution within the clusters is visually represented in the mosaic plot (Fig. 7), where the blue areas indicate overrepresentation and the red areas indicate underrepresentation.

**Table 3:** Generational composition of Self-Organizing map (SOM) clusters (*n* = 2,507). The table presents the distribution of the five generational cohorts across the four SOM-derived clusters, alongside Chi-square test results. Percentages indicate each generation’s relative share within each cluster.

Generations	Cluster 1 *n* (%)	Cluster 2 *n* (%)	Cluster 3 *n* (%)	Cluster 4 *n* (%)	Statistical test ^a^
Generation Z	68 (19.5)	29 (6.8)	104 (20.7)	168 (13.7)	Χ² (12) = 181.05
Generation Y	96 (27.6)	57 (13.3)	175 (34.8)	279 (22.7)	p-value < 2.2e-16
Generation X	87 (25.0)	131 (30.6)	138 (27.4)	371 (30.2)	*n* = 2,507
Baby Boomers	74 (21.3)	178 (41.6)	74 (14.7)	373 (30.4)	
Traditionalists	23 (6.6)	33 (7.7)	12 (2.4)	37 3.0)	

^a^In case the number of cases in the cross-table is greater than 5: Chi-square test of independence.

Cluster 1, *“All-rounders”*, displays a generational mix, but is dominated by Generation X and Baby Boomers (Table 3; Fig. 7). These respondents reported moderate digital competence (S45) and rely on a combination of personal knowledge, printed maps and social recommendations (Figures S39, S34 and S40). While technology plays a minor role in their trip planning (Figures S34, S39, S40), younger members of this cluster tend to use digital tools more often, whereas older ones remain tied to traditional sources (Figures S39, S40), and technology plays only a minor role in trip planning. This cluster represents a transitional group, bridging analog and digital habits.

Cluster 2, “Traditional Planners and Navigators”, is composed primarily of Baby Boomers and Traditionalists, who report low digital competence (Figure S45 in Supplementary Information S3). The low presence of younger generations suggests that this type of planning behaviour is gradually declining. This group showed the strongest preference for printed materials and non-digital planning resources, physical landmarks and minimal reliance on mobile tools is (Figures S34, S37, S41–S44). Their structured, analog approach reflects familiarity and a desire for predictability in trip planning. They plan visits well in advance.

Cluster 3, *“Tech-Savvy Navigators”* is primarily composed of Generation Z and Millennials, the most digitally fluent age groups. Most rate themselves as “Advanced” or “Very Advanced” in digital skills (S45) and prefer digital tools for planning and navigation (Figures S35, S36, S44). Personal knowledge, printed tools, and social input played minimal roles for this group (Figures S34, S39, S40), reflecting a digital-native approach to outdoor recreation. Older generations are nearly absent from this cluster, reflecting a strong generational divide in technology adoption. The near-total absence of older generations in this cluster underscores the generational divide in technology adoption.

Cluster 4, *“Balanced Adapters”* includes a mix of Generation X, Millennials, and younger Baby Boomers (Table 3, Fig. 7). Unlike Cluster 3, this group represents a generational transition: younger members are fully comfortable with digital tools, while older individuals continue to draw on traditional resources. They engage in digital planning (Figures S35–S36) and frequently use mobile internet during navigation (Figure S44), yet remain open to analogue tools as situational backups (Figures S33–S34, S37). This hybrid strategy reflects a flexible, context-dependent approach.

Overall, Clusters 3 and 4 reflect a generational shift toward digital-first recreation, especially among younger cohorts. In contrast, Clusters 1 and 2 illustrate continued reliance on non-digital tools, particularly among older generations. These patterns highlight the importance of designing inclusive infrastructure and communication strategies that address diverse digital readiness levels and planning styles.

#### Relationship between generations, digital skills, and digital tool usage

**Fig. 8:**
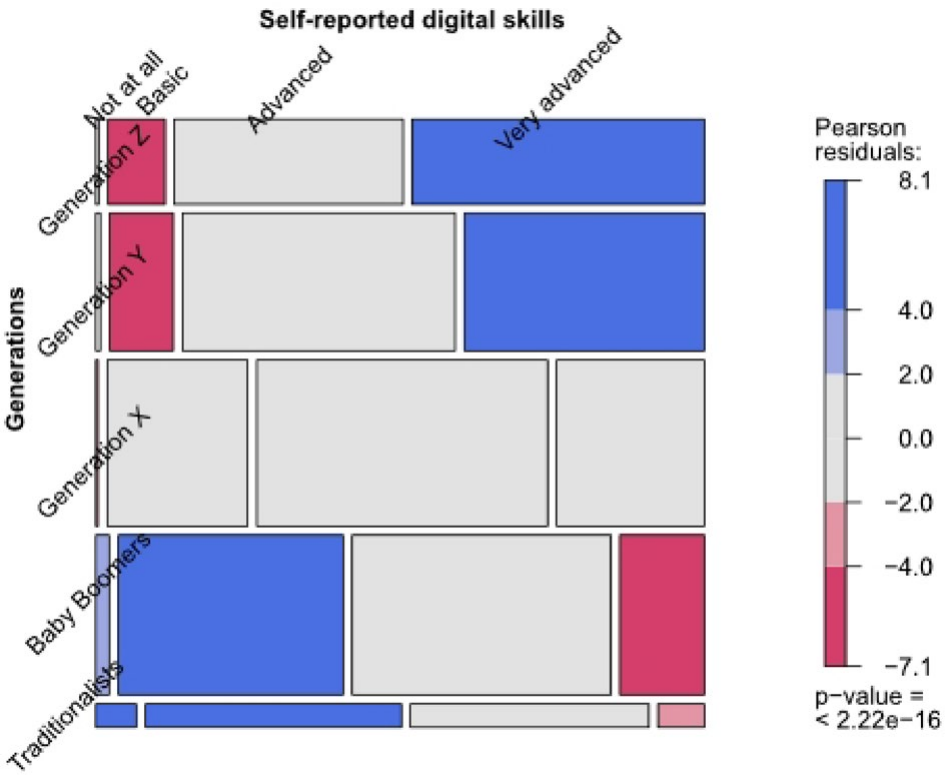
Mosaic plot showing self-reported digital skills across generations (*n* = 2,463). Blue areas indicate overrepresentation within a specific digital skills level, while red areas indicate underrepresentation. The colour gradient reflects the Pearson residuals, with darker tones denoting a greater deviation from the expected frequencies under the assumption of independence.

Self-reported digital competence varied significantly across generations (Table 4; Fig. 8). A Fisher’s Exact Test confirmed this association was statistically significant (*p* < 1e-7), with a clear generational gradient.

**Table 4:** Self-reported digital competence across generational groups (*n* = 2,463). The table shows the distribution of self-assessed digital skill levels by generation, ranging from “not at all” to “very advanced”.

Self-reported digital competence	Generation Z *n* (%)	Generation Y *n* (%)	Generation X *n* (%)	Baby Boomers *n* (%)	Traditionalists *n* (%)	Statistical test ^a^
Not at all	2 (0.5)	5 (0.8)	3 (0.4)	16 (2.3)	7 (7.0)	p-value < 2.22e − 16
Basic	36 (9.8)	65 (11.0)	173 (24.2)	266 (38.7)	44 (44.0)	*n* = 2,463
Advanced	144 (39.3)	278 (46.9)	358 (50.0)	306 (44.5)	41 (41.0)	
Very advanced	184 (50.3)	245 (41.3)	182 (25.4)	100 (14.5)	8 (8.0)	

^a^In case number of cases in the cross-table is smaller than 5: Fisher’s Exact Test (with simulated p-value based on 1e + 07 replicates; two-sided).

Generation Z showed the highest digital proficiency, with nearly 90% rating themselves as “Advanced” or “Very Advanced.” Millennials (Gen Y) followed closely, with a strong majority also reporting high competence. Generation X showed a more mixed profile, although “Advanced” was the most frequent rating. The Baby Boomers and Traditionalists had the lowest levels of self-assessed digital skills. Over 40% of Traditionalists rated their competence as “Basic” or “Not at all,” and fewer than 10% as “Very Advanced.”

These findings reveal a generational shift in digital skills. A clear transition from the older to the younger generations can be observed as each successive generation rates their competence higher.

### Digital competence and GPS/GNNS usage

**Fig. 9:**
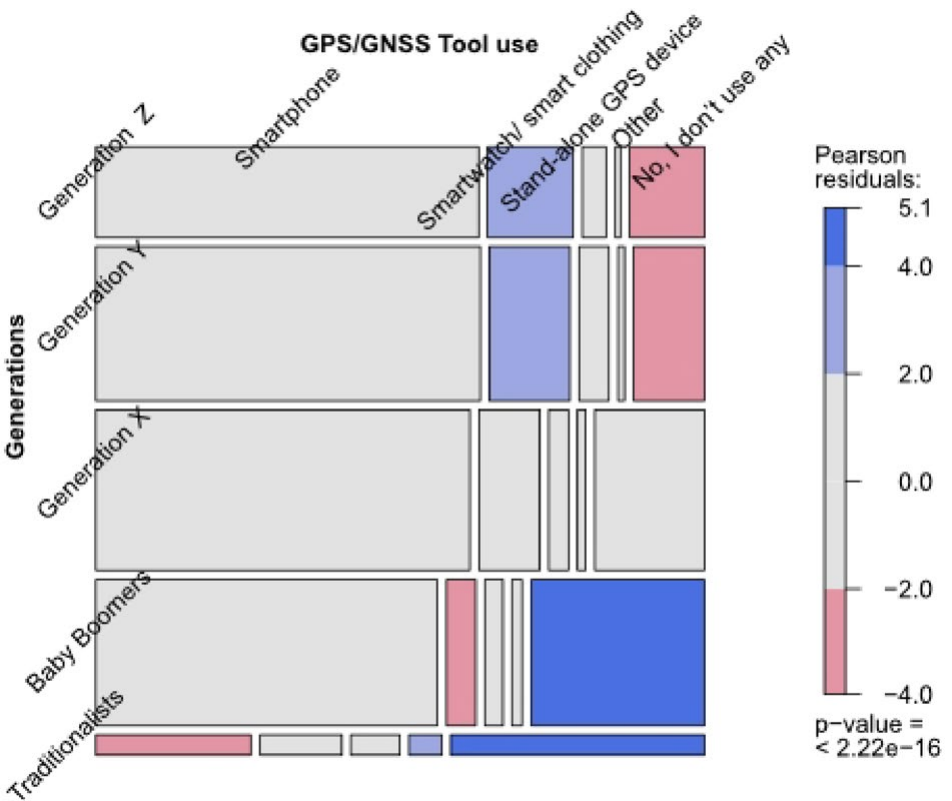
Mosaic plot showing GPS/GNSS navigation tool use by generation during forest visits (*n* = 1,977). Blue areas indicate overrepresentation and red areas indicate underrepresentation of each generation across device types. The colour scale reflects Pearson residuals; darker shades indicate stronger deviations from the expected values under the assumption of independence.

The analysis of GPS/GNSS tool use (Table 5; Fig. 9) confirms these generational patterns. Generation Z reports the highest frequency of digital tool use, with 89% reporting frequent or occasional use and only 11% reporting no use (Figure S14; Supplementary Table S3, Supplementary Information S2). Millennials (Generation Y) showed a similar pattern, with the majority using digital tools either sometimes or frequently. Generation X presented a more balanced profile, though a substantial share also reported regular use.

**Table 5:** GPS/GNSS-based navigation tool use during forest visits (*n* = 1,977). The table shows the generational differences in the reported use of smartphones, smartwatches or smart clothing, stand-alone GPS devices, and other tools. A Fisher’s exact test indicates a statistically significant association between generation and device use (*p* < 2.22e − 16).

Use of GPS/GNSS-based navigation tools during your tour	Generation Z *n* (%)	Generation Y *n* (%)	Generation X *n* (%)	Baby Boomers *n* (%)	Traditionalists *n* (%)	Statistical test^a^
Smartphone	210 (66.9)	358 (67.2)	362 (65.3)	302 (59.7)	19 (27.1)	*p* < 2.22e − 16
Smartwatch/smart clothing	47 (15.0)	75 (14.1)	59 (10.6)	26 (5.1)	10 (14.3)	*n* = 1,977
Stand-alone GPS device	13 (4.1)	28 (5.3)	19 (3.4)	16 (3.2)	6 (8.6)	
Other	3 (1.0)	6 (1.1)	8 (1.4)	9 (1.8)	4 (5.7	
No, I don’t use any	41 (13.1)	66 (12.4)	106 (19.1)	153 (30.2)	31 (44.3)	

^a^In case the number of cases in the cross-table is smaller than 5: Fisher’s Exact Test (with simulated p-value based on 1e + 07 replicates; two-sided).

In contrast, Baby Boomers and especially Traditionalists reported significantly lower levels of digital tool use. Approximately one-third of Traditionalists reported never using digital tools during forest visits, compared to just 11% in Generation Z. A Fisher’s Exact Test confirmed that these differences were statistically significant (*p* < 2.22e − 16, *n* = 1,977). The mosaic plot (Fig. 9) visually emphasises this trend: younger generations are overrepresented in the use of smartphones and wearables, while older adults are more likely to navigate without digital support. These results underscore that digital navigation behaviours are generationally patterned: younger users rely on mobile and wearable technology, while older visitors stick to traditional methods.

Patterns of post-visit digital engagement differed sharply between generations. As shown in the mosaic plots (Figures S23–S32), Generation Z and Millennials were significantly more active in photo sharing (Figure S23), tour rating (Figure S24), route sharing (Figure S28), and performance comparisons (Figure S30). These patterns underscore their strong preference for digital interaction and community feedback. In contrast, the Baby Boomers and Traditionalists exhibited notably lower levels of post-visit digital engagement, suggesting a continued preference for more offline or solitary experiences (Table S3, Supplementary Information S2).

#### Relationship between generations and forest visitation

**Fig. 10:**
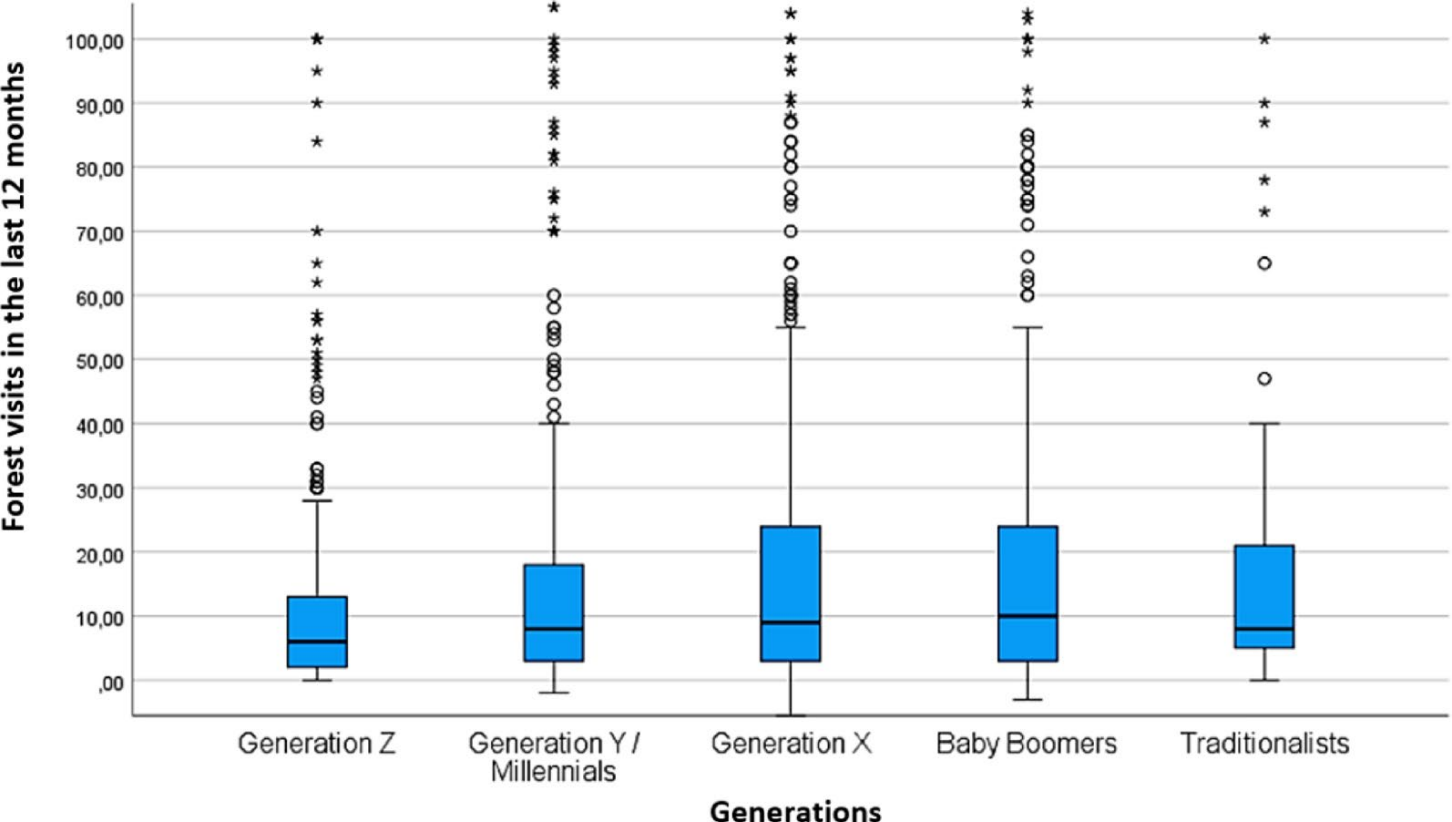
Self-reported forest visitation frequency across generations (*n* = 2,507). Boxplots show the distribution of forest visits within the past 12 months, by generation. The central line represents the median; boxes indicate the interquartile range (IQR), and whiskers extend to 1.5 × IQR. Outliers are shown as individual points. Figure created by the authors using IBM SPSS Statistics (version 28.0.1.1, IBM Corp., Armonk, NY, USA).

Generational differences in forest visitation frequency were statistically significant (χ² = 17.475, df = 4, *p* = 0.0016; see Supplementary Table S8 in S4). Compared with Generation X, Generation Z reported significantly fewer forest visits. Baby Boomers and Traditionalists exhibited more evenly distributed visit patterns throughout the year, with Traditionalists reporting the highest median number of visits in the past 12 months (Fig. 10).

Significant generational differences also emerged in route planning preferences (χ² = 25.208, df = 8, *p* = 0.0014). Gen Z and Millennials most often used pre-prepared digital route suggestions, while Boomers preferred to plan their routes. Traditionalists showed low engagement with preplanned routes, suggesting reliance on familiar paths or in-person guidance.

The planning time of the forest visits also varied by generation (Figure S4, Table S3). Generations Z and Millennials prefer planning tours close to the visit date, either the day before or on the day of the trip. Generation X has a more balanced approach but tends to plan the day before. Baby Boomers generally plan several days to a week before. Traditionalists, the oldest group, often vary their planning times depending on the trip, with a significant number planning several days in advance (Table S3, Figure S4).

The spatial analysis using the Getis-Ord Gi* tool (Figures S46–S54, Supplementary Information S4) revealed forest visitation patterns. Notably, younger generations have more dispersed hotspots, reflecting diverse recreational preferences and wider exploration. (Figures S346 and S44). In contrast, older generations preferred familiar or nearby areas, resulting in more concentrated hotspots, particularly in well-established green spaces like the Wienerwald Biosphere Reserve and Donauauen National Park (Figures S48–S50).

#### Relationship between digital skills and forest visitation

Our analysis revealed that individuals with higher digital competence tended to report slightly more frequent forest visits than those with lower skill levels. This pattern suggests that digital proficiency may support forest recreation by making it easier to access planning tools, navigate on-site, and discover new destinations (Figures S53–S54 in Supplementary Information S4).

A Kruskal–Wallis test confirmed statistically significant differences in annual forest visit frequency across digital competence categories (χ² = 18.63, df = 4, *p* = 0.0009), though the effect size was small. This indicates that digital skills alone are not strong predictors of how often people engage in forest recreation (Table S8, S4.2.2 in Supplementary Information S4).

To investigate the group-level differences, Dunn’s post hoc test with Bonferroni correction was applied. Results showed that respondents with Advanced digital skills reported significantly more visits than those with Basic skills (*p* = 0.044). Differences between Highly Advanced and Basic users were only marginally significant (*p* = 0.062), and no other pairwise comparisons reached significance (Table S9–S10 in Supplementary Information S4). A Spearman correlation further confirmed a weak but statistically significant positive relationship between digital competence and forest visit frequency (ρ = 0.066, *p* = 0.0009). While this indicates a slight trend, the strength of the association is too limited to be of practical importance.

In summary, although higher digital skills may offer some facilitative benefits, such as easier access to navigation tools or better discovery of forest areas, they do not constitute a major driver of forest recreation frequency. Other factors, including time availability, mobility, and proximity to green spaces, likely exert a stronger influence on recreational behaviour.

## Discussion

### Generational patterns in digital competence and outdoor engagement

This study addresses generational differences in digital competence and tool use in forest recreation, highlighting key implications for inclusive visitor planning and digital infrastructure development. As expected, younger generations reported higher digital skills^63,95,96^. However, digital engagement is shaped not only by individual competence but also by situational factors such as context, motivation, social setting, and familiarity with the environment, supporting the concept of situational engagement, where technology use is adaptive rather than fixed^97^.

Our findings align with international typologies of digital behaviour^46,98,99^, such as “Tech-Savvy Explorers”^100,101^, “Traditional Planners”^31,102^, “Hybrid Adopters”^46,98^ and “Situational Switchers”, who are defined by context-based decision-making and use of technology influenced by experience level, social setting (eg, group composition) or familiarity with the environment^37,99^ and adaptive information behaviour in recreation contexts. These categories were reflected empirically in our Self-Organizing Map (SOM) clustering, which segmented forest visitors into four groups: All-rounders, Traditional Planners and Navigators, Tech-Savvy Navigators, Balanced Adapters and Hybrid Users.

Digitalisation is fundamentally transforming outdoor recreation. Apps, GPS/GNSS devices, and social media shape how visitors plan, navigate, and share their forest experiences^40,103,104^. This shift spans the entire visitor journey, from planning to navigation and sharing, and reflects changing expectations, especially among younger users in Clusters 3 and 4^31,105^, who show strong engagement with digital tools for planning and navigation.

Tech-Savvy Navigators (Cluster 3)—typically Gen Z and Millennials—integrated mobile apps, GPS, and online platforms throughout their forest visits. Traditional Planners and Navigators (Cluster 2), often older adults, preferred printed maps, structured offline planning, and interpersonal advice. Balanced Adopters (Cluster 4) blended digital and analog tools, depending on the context. All-rounders (Cluster 1) showed flexible, experience-based behaviours influenced by group setting, familiarity and task.

Importantly, our findings suggest that high digital affinity does not automatically translate into greater outdoor engagement. Despite their technological fluency, younger generations, especially Gen Z, often report less frequent forest visits than older cohorts, a trend mirrored in prior research^69,106^. While some younger people increased their outdoor activity during the COVID-19 pandemic, the broader pattern suggests that digital competence does not guarantee higher nature interaction. While digital natives are characterised by high connectivity^63,101,107^, their outdoor interaction appears more fragmented. These findings highlight the need to embed digital touchpoints within outdoor experiences while still promoting deeper, more sustained engagement with nature^108,109^.

Moreover, even the most digitally skilled users continue to rely on experiential knowledge. Across all clusters, visitors combined digital tools with personal memory, spatial awareness, or advice from others. This suggests that digital skills complement rather than replace traditional navigation strategies, reinforcing the enduring value of analog orientation.

### Digital visitor segments and digital engagement strategies

#### Managing diverse digital visitor profiles

The SOM identified four distinct visitor types, each reflecting a different stage of digital engagement, offering a practical framework for tailoring recreation planning and communication strategies (Table 6).

**Table 6:** Digital visitor segments and corresponding recommendations for recreation management. The four Self-Organizing map (SOM) clusters represent distinct visitor profiles based on digital engagement. Descriptions summarise behavioural traits, while the management focus outlines targeted strategies to support inclusive and adaptive communication and planning.

Cluster	Label	Description	Management Focus
1	All-rounders	Situationally adaptive, flexible users who switch tools based on experience and context	Multi-channel access; support analog-digital flexibility
2	Traditional Planners and Navigators	Older users relying on printed maps and structured planning	Maintain printed materials and signage; introduce simple digital tools (e.g., QR codes)
3	Tech-Savvy Navigators	Digital-First Users. Predominantly younger users who integrate apps, GPS, and real-time data	Provide robust digital services, including mobile apps, interactive maps, and real-time alerts
4	Balanced Adapters	Transitioning gradually from analog to digital formats	Offer hybrid solutions; support onboarding into digital platforms

*Tech-Savvy Navigators (Cluster 3)*, primarily Gen Z and Millennials, who fully integrated mobile apps, GPS, and online content throughout their forest visits (Table 5; Fig. 9; Figures S8–S10, S14–S20 in Supplementary Information S2)^100,101,110^. These users benefit from dynamic, mobile-optimised tools supporting both pre-trip planning and real-time decision-making. Features such as live geospatial data, safety alerts, and regulation updates are essential. Parks and recreation professionals can leverage this generation’s affinity for digital tools by designing tech-supported outdoor activities that bridge digital engagement and natural experiences.

*Balanced Adapters (Cluster 4)* are transitioning to digital formats while still using printed materials. This group responds best to user-friendly hybrid systems that combine analog and digital resources, like downloadable maps, QR-code information points, and simplified interfaces that are available alongside traditional materials.

*Traditional Planners and Navigators (Cluster 2)* favour analog navigation, interpersonal advice, and structured planning. Although digital tools are increasingly available, this group values predictability and familiarity. Support should prioritise high-quality analog infrastructure without pressuring digital adoption^31,98^.

*All-rounders* are situationally adaptive *(Cluster 1) and* adjust tool use based on group composition, familiarity, or task. They blend digital tools with memory and interpersonal recommendations. This segment requires both mobile coverage and analog systems, such as printed guides. Tools integrating formal geospatial data with community-sourced knowledge can enhance context-aware decision-making^37,99,111^.

Across all groups, user-generated content (UGC)—such as trail reviews or safety updates—emerges as a key layer of value. Encouraging participatory contributions can foster community learning and build dynamic, user-centered information ecosystems.

#### Inclusive infrastructure for digital recreation

Recreation management must account for generational disparities in digital competence, as revealed by the SOM clusters. This calls for differentiated infrastructure strategies that ensure equitable access across digital and analog systems.

Digital tools should provide real-time, location-based information (e.g., trail conditions, closures), function offline in low-connectivity areas, and feature intuitive, multilingual interfaces. For users in Clusters 2 and 4, simplified onboarding, such as downloadable maps or QR-code access, can ease transitions into digital platforms and reduce barriers for less confident users. Analog infrastructure remains essential. Many visitors, especially older cohorts, continue to rely on printed maps, signage, and local knowledge. Maintaining analog systems alongside digital offerings supports inclusive and flexible navigation.

Beyond wayfinding, digital platforms can enhance environmental education. Apps with interactive content on biodiversity, conservation, or wellness activities (e.g., forest bathing) are well suited to digital-native audiences, especially when gamified or personalised^112,113^. Data-driven visitor management (DVM) systems using GPS, behavioural analytics, and user input can support real-time monitoring, congestion management, and adaptive planning. However, such tools must adhere to ethical standards, ensuring transparency, privacy, and inclusivity, particularly in sensitive environments^114^.

Digitalisation carries risks, such as overcrowding and misinformation via social media^32^. Maintaining digital systems requires ongoing investment^40,110^. To address these challenges, managers should adopt standardised, cross-park frameworks rooted in ethical and sustainable design. A hybrid infrastructure balancing analog and digital access supports resilient, inclusive recreation management.

### Strengths and limitations of the methodology

#### Data collection and sample

One of the main strengths of this work is the large sample of respondents and its distribution, which corresponds to the population composition of the studied region, considering various demographic characteristics such as age, gender, and living location (Section “Online panel survey details” and Supplementary Information S1, Table S2). However, a detailed inspection and comparison of the generational distribution between the census data and the collected sample revealed a slight bias related to the oldest generations. Assigning respondents’ dates of birth to generational classifications and comparing the sample distribution with census data (Table 1 and Table S7) shows that Traditionalists are slightly underrepresented in the collected sample, while Baby Boomers are somewhat overrepresented compared to census data^115^. Nevertheless, the underrepresentation of older participants is a well-known issue in socio-empirical research based on online panels^116,117^. Table 7 provides an overview of generational shares according to the census and collected data.

**Table 7:** Overview of the population composition in the study area (Statistik Austria, 2024) and the collected sample grouped by generations. Census data are based on the population aged over 15 years (Statistik Austria, 2024). The table compares generational shares in the total population with those in the survey sample.

Generation	Population*	Population %	Sample	Sample %
Generation Z	350,804	16%	36,900	15%
Generation Y	516,619	24%	60,700	24%
Generation X	598,674	28%	727	29%
Baby Boomers	506,758	24%	699	28%
Traditionalists	176,613	8%	105	4%
	2,149,468	100%	2,507	100%

*Population > 15 years.

Another point of discussion concerns the mode of data collection. Online panels involve more digitally skilled respondents, which can be considered a notable limitation, especially in the case of forest visitors with limited technology access and low digital skills. The issue of data collection modes and associated biases is widely discussed in the context of tourism and outdoor recreation^106,118^ as well as in the field of digital competence measurement^119,120^. Therefore, we acknowledge that the digital skills reported via online panels might be slightly overestimated compared to field studies that capture less technology-affine visitors.

#### Assessment of digital skills and recreational behaviour

In our study, an online panel survey was used to capture recreational behaviour and visitor characteristics. We relied on visitor-reported data regarding information sources and technology use in the context of forest recreation. Digital competence was measured through self-assessment, a widely used approach in large-scale studies^75,121^. While efficient and scalable, the survey-based self-assessment approach may lead to overestimations, as respondents tend to rate their skills higher than they are^120^ and this can skew the results. There is an ongoing scholarly discussion highlighting the potential limitations of this approach^76,122^.

Beyond surveys, alternative methods for measuring digital skills in society, such as standardised tests, framework-based assessments^76,122^, mixed-method approaches^123^, specialised tools^124^, ethnographic methods^119^, and gamification^125^ could provide more objective or behavioural insights. Future research could benefit from mixed methods that combine self-reports with behavioral assessments or digital trace data to more accurately capture usage patterns.

While the SOM methodology provided clear insights into digital tool usage and recreational behaviour, revealing patterns that traditional clustering might overlook^126^, a few limitations need to be acknowledged. The interpretation of cluster boundaries involves a degree of subjectivity, requiring a deep understanding of the data and context. Without qualitative validation, such as interviews or direct feedback, there remains potential variability in how the clusters are characterised. Although the SOM excels in quantitative clustering, it lacks the qualitative depth needed to fully confirm the behaviours and preferences within each group.

To address this, future studies should incorporate participatory mapping and community-based data collection methods, which have been shown to capture a broader spectrum of user needs and behaviours, particularly among populations typically underrepresented in digital datasets^62^. Tailoring outreach based on sociodemographic profiles, such as age or tech familiarity, can help ensure more inclusive and context-sensitive data collection^98^.

## Conclusions and outlook for future research

This study demonstrates the value of understanding generational differences in digital skills and recreation behaviour to inform the planning and management of forest destinations. The findings reveal a clear need for inclusive strategies that reflect the diverse ways visitors access, plan, and experience outdoor spaces in a digital age. By aligning digital innovation with ethical design and accessibility, forest managers can better serve all generations, enhancing both user experience and long-term sustainability.

As technology continues to evolve, the implications for outdoor recreation will shift accordingly. Future research should adopt longitudinal perspectives to monitor how digital behaviours change over time, particularly as younger generations age and new digital norms emerge. Tracking these developments is essential for anticipating changes in tool use, engagement patterns, and infrastructure demands.

To support this transition, urban green space management must prioritise digital tools that are intuitive and accessible across skill levels, apply transparent and responsible data practices, and involve diverse stakeholders in both platform development and spatial planning. Participatory approaches are especially critical to ensure that digital transformation does not reinforce existing divides but instead promotes more equitable and inclusive access to natural spaces.

Beyond the local scale, this research supports broader efforts to integrate ecosystem benefits into global public policy. By embedding forest recreation and its digital dimension into sustainable urban development strategies, planners can enhance the livability of cities while supporting ecological resilience. Aligning technology with participation and stewardship is the key to future-ready forest landscapes.

While grounded in the Vienna Metropolitan Area, the findings may apply to other digitalised urban contexts; future research should test their relevance in rural, cross-cultural, and less-connected settings.

## Electronic supplementary material

Below is the link to the electronic supplementary material.

Supplementary Material 1

Supplementary Material 2

Supplementary Material 3

Supplementary Material 4

## Data Availability

Relevant data supporting the findings of this study are included within the manuscript and supplementary information files. The full datasets generated and/or analyzed during the study, as well as the R code used for the Self-Organizing Map (SOM) analysis, are not publicly available due to the necessity for additional data cleaning to ensure the dataset meets quality and ethical standards for broader research applications. However, these datasets are available from the corresponding author upon reasonable request.
