# Supplementary material for: Understanding generational differences in digital skills and recreational behaviour for effective visitor management in forest destinations
Source: Sci Rep. 2025 May 23;15:17887. doi: 10.1038/s41598-025-02036-5 (PMC12098668; doi:10.1038/s41598-025-02036-5)
Supplement: Supplementary file 1 — Supplementary Material 1 [file 41598_2025_2036_MOESM1_ESM.docx]

**Supplementary Information (S1)**

**Survey content and regional population distribution used for sample design**

***Table S1.*** *Survey questions on forest visitation, planning, navigation behaviour, and digital skills in the Vienna metropolitan area.*

This table presents selected survey questions used to investigate forest visitation patterns, planning and navigation behaviours, and digital skills among residents in the Vienna metropolitan area. The questions are designed to capture the key aspects of cultural ecosystem services (CES), particularly recreation, relaxation, and engagement with urban and peri-urban forests. Topics include information source usage, changes in visitation patterns pre- and post-COVID-19, digital tool usage, and general digital skills. The data provide insights into digital engagement in forest contexts, informing forest management and sustainable recreation planning.

| Survey question number | Survey question | Answer options |
| --- | --- | --- |
| F002 | How often do you visit forest areas in the metropolitan area of Vienna? |  |
|  |  | almost daily (more than 4 times a week) / every day if possible |
|  |  | 3-4 times a week |
|  |  | 1-2 times a week |
|  |  | around 2-3 times a month |
|  |  | once in a month |
|  |  | several times a year |
|  |  | once a year |
|  |  | less than once per year |
|  |  | one-time visit |
|  |  | never |
| F004 | How many times did you visit forest areas in the last 12 months? |  |
|  | Please enter a number (if none, then enter 0) |  |
|  |  | this/last Autumn? (Sept., Oct., Nov.) |
|  |  | in Summer (June, July, Aug.)? |
|  |  | in Spring (Mar., Apr., May) |
|  |  | in Winter? (Dec., Jan., Feb.) |
| F005 | How has your actual frequency of visits to the forest changed compared to BEFORE the COVID-19 pandemic? |  |
|  |  | More frequently visited than before the pandemic |
|  |  | Remained unchanged |
|  |  | Less frequently visited than before the pandemic |
|  |  | Never visited, although visited before the pandemic |
|  |  | Never visited, never visited before the pandemic |
|  |  | More frequently visited than before the pandemic |
|  |  | Remained unchanged |
| F006 | How has your frequency of visits to the forest changed DURING the COVID-19 pandemic (2020-2021) compared to BEFORE the pandemic? |  |
|  |  | Visited more often during pandemic than before |
|  |  | Remained unchanged during pandemic |
|  |  | During pandemic visited less frequently than before |
|  |  | Never visited during pandemic, although previously visited |
|  |  | During pandemic and previously never visited |
|  |  |  |
|  |  | Visited more often during pandemic than before |
|  |  | Remained unchanged during pandemic |
| F015 | How far in advance do you usually plan your tours? |  |
|  |  | on the day of the tour |
|  |  | the day before |
|  |  | several days before |
|  |  | about a week in advance |
|  |  | always different, depending on the tour |
|  |  | not at all |
|  |  | on the day of the tour |
|  |  | the day before |
|  |  | several days before |
|  |  |  |
| F016 | How often do you use the following information sources to PLAN your visit to urban forests? |  |
| F016a | My own knowledge |  |
| F016b | Local knowledge of friends, family, etc. |  |
| F016c | Printed maps, travel guides, tour flyers and books |  |
| F016d | Digital maps PC / Apps for Smartphone, Tablet, Smartwatch and digital maps for PC |  |
| F016e | Internet Search |  |
| F016f | Information from tourist information offices (not via the internet) |  |
| F016g | Journalistic reports (e.g. magazines, TV) |  |
|  |  | very often |
|  |  | often |
|  |  | sometimes |
|  |  | rarely |
|  |  | never |
| F019 | How often do you use the following information sources DURING your visit in urban forests? |  |
| F019a | Own knowledge |  |
| F019b | Local knowledge of friends, family, etc. |  |
| F019c | Signposts and information boards in the forest |  |
| F019d | Printed maps, guides, tour flyers and books |  |
| F019e | Digital maps / Apps for smartphones, tablet, and smartwatches |  |
| F019f | Mobile Internet Search |  |
| F019g | GPS device (not GPS via smartphone) |  |
| F019h | Printouts from the internet, digital maps |  |
|  |  | very often |
|  |  | often |
|  |  | sometimes |
|  |  | rarely |
|  |  | never |
| F020 | Do you carry your mobile phone during your outdoor recreational activities? |  |
|  |  | yes, often |
|  |  | yes, sometimes |
|  |  | no, never |
| F022 | Do you use any digital tools during your trip to / in the forest? (e.g. smart phone, mobile phone, GPS) |  |
|  |  | yes, often |
|  |  | yes, sometimes |
|  |  | no, never |
| F023 | Do you use any GPS / GNSS based navigation tools during your tour? |  |
|  |  | yes, in my smartphone |
|  |  | yes, in smart watch, smart cloth, shoes, etc. |
|  |  | yes, in stand-alone GPS device |
|  |  | no, I don't use any |
|  |  | I don´t know |
|  |  | yes, other |
| F030 | What kind of information do you share after your forest visit? What digital community features do you use? |  |
| F030a | Photos |  |
| F030b | Sharing route (GPS / GNSS tracks) or finished tours |  |
| F030c | Text messages, chat |  |
| F030d | Rating of tours |  |
| F030e | Forums, giving feedback |  |
| F030f | Reports and summaries |  |
| F030j | Sharing activity parameters |  |
| F030k | Comparison of performance |  |
| F030l | Share tips, read comments |  |
|  |  | yes, publicly visible |
|  |  | yes, visible to friends/family only |
|  |  | yes, I keep it private |
|  |  | no |
| F053 | How would you rate your general digital competence? |  |
|  |  | very advanced |
|  |  | advanced |
|  |  | basic |
|  |  | not at all |
|  |  | very advanced |
| F054 | How often do you use digital technologies and devices in your daily life? |  |
|  |  | several times a day |
|  |  | daily |
|  |  | several times a week |
|  |  | rarely |
|  |  | never |

***Table S2.*** *Regional population distribution and quota framework used for survey sampling.*

This table outlines the regional population distribution used to develop the survey’s quota sampling framework. Data are based on official 2023 statistics by postal code, district, and municipality. To ensure proportional representation, each region’s population share informed the allocation of survey respondents. The coding system was developed to facilitate data processing across Vienna’s urban, suburban, and surrounding rural zones. The quota-based approach enhances the demographic and spatial representativeness of the sample, supporting robust analysis of forest visitation and digital behaviours across the metropolitan area.

| **Postal Code** | **District** | **Municipality** | **Population 2023** | **Quotas (%)** | **Coding** |
| --- | --- | --- | --- | --- | --- |
| **TOTAL** |  |  | **2 679 090** | **100,0%** |  |
| **Wien** |  |  | **1 982 097** | **74,0%** |  |
| **R 1.1 - Wien Innen** | **= Vienna Centre** |  | **331 911** | **12,4%** | **11** |
| 1010 | Innere Stadt |  | 16 620 |  |  |
| 1030 | Landstraße |  | 96 756 |  |  |
| 1040 | Wieden |  | 33 633 |  |  |
| 1050 | Margareten |  | 55 018 |  |  |
| 1060 | Mariahilf |  | 31 423 |  |  |
| 1070 | Neubau |  | 31 581 |  |  |
| 1080 | Josefstadt |  | 24 674 |  |  |
| 1090 | Alsergrund |  | 42 206 |  |  |
| **R 1.2 - Wien Innen-Ost** | **= Vienna Centre East** |  | **193 959** | **7,2%** | **12** |
| 1020 | Leopoldstadt |  | 108 269 |  |  |
| 1200 | Brigittenau |  | 85 690 |  |  |
| **R 1.3 - Wien Süd** | **= Vienna South** |  | **545 616** | **20,4%** | **13** |
| 1100 | Favoriten |  | 218 415 |  |  |
| 1110 | Simmering |  | 109 038 |  |  |
| 1120 | Meidling |  | 100 281 |  |  |
| 1230 | Liesing |  | 117 882 |  |  |
| **R 1.4 - Wien West** | **= Vienna West** |  | **514 058** | **19,2%** | **14** |
| 1130 | Hietzing |  | 55 568 |  |  |
| 1140 | Penzing |  | 96 828 |  |  |
| 1150 | Fünfhaus |  | 76 109 |  |  |
| 1160 | Ottakring |  | 102 444 |  |  |
| 1170 | Hernals |  | 56 033 |  |  |
| 1180 | Währing |  | 51 559 |  |  |
| 1190 | Döbling |  | 75 517 |  |  |
| **R 1.5 - Wien Nordost = Vienna Northeast** | |  | **396 553** | **14,8%** | **15** |
| 1210 | Floridsdorf |  | 183 895 |  |  |
| 1220 | Donaustadt |  | 212 658 |  |  |
| **Niederösterreich** |  |  | **696 993** | **26,0%** |  |
| **R.2.1 - Wiener Umland-Nordteil <AT126> = Vienna Surrounding Communities North** | |  | **342 995** | **12,8%** | **21** |
| **R2.2 - Wiener Umland-Südteil <AT127> = Vienna Surrounding Communities South** | |  | **353 998** | **13,2%** | **22** |
