# Supplementary material for: Understanding generational differences in digital skills and recreational behaviour for effective visitor management in forest destinations
Source: Sci Rep. 2025 May 23;15:17887. doi: 10.1038/s41598-025-02036-5 (PMC12098668; doi:10.1038/s41598-025-02036-5)
Supplement: Supplementary file 2 — Supplementary Material 2 [file 41598_2025_2036_MOESM2_ESM.docx]

**Supplementary Information (S3)**

**Trends Across SOM Clusters: Digital Competence and Tool Usage Before and During Forest Visits**

***Table S4.*** *Comparative analysis of tool usage trends across Self-Organising Map (SOM) clusters in the Vienna Metropolitan Area.*

This table presents the distribution of digital tool usage, planning preferences, navigation behaviours, and self-reported digital competence across four SOM clusters. Clusters were derived from respondents’ digital behaviour and skills patterns, as identified in the main analysis. Percentages reflect the proportion of respondents within each cluster who selected a given response. Variable names (e.g. F016a, F019c) correspond to the original survey items (see Table S1 in Supplementary Information S1). Mosaic plots for each variable (Figures S32–S45) provide visual representations using Pearson residual-based shading to highlight deviations from statistical independence.

The mosaic plots illustrate dependencies between the SOM clusters and various characteristics related to tool use. Forest visit frequency characteristics include tools used for planning forest visits (Figures S32–S38). Navigation tools include those used for navigation during forest visits (Figures S39–S44). The digital competence is illustrated by self-reported digital competence by generation (Figure S45).

Table S4 provides information on whether there are significant differences between groups or not (Chi-square test of independence). To recognize categories that differ most, we **include** **MOSAIC PLOTS using residual-based shadings (Pearson residuals).** The colours represent the level of the residual for that cell/combination of levels. The legend is presented on the right. Blue shading means there are more observations in that cell than would be expected under the null model (independence). Red means there are fewer observations than would have been expected. Grey cells indicate that the corresponding Pearson residual is within a 95% confidence interval around zero (independence). Note that p-values in the tables have not been corrected for multiple testing since most significant p-values are much smaller than any of the usually used significance levels (0.05, 0.01, etc.).

| Cluster | Cluster 1  n (%) | Cluster 2  n (%) | Cluster 3  n (%) | Cluster 4  n (%) | Statistical test ^a,b^ |
| --- | --- | --- | --- | --- | --- |
| **32** (F016a). Own knowledge usage before forest visits n (%) |  |  |  |  |  |
| Never | 5 (1.3) | 37 (7.8) | 64 (11.6) | 0 (0.0) | p-value = 1e-07 |
| Rarely | 19 (4.9) | 14 (2.9) | 90 (16.3) | 1 (0.1) | n = 2362 |
| Sometimes | 92 (23.7) | 28 (5.9) | 215 (38.9) | 60 (6.4) |  |
| Often | 139 (3.7) | 80 (16.8) | 157 (28.4) | 327 (34.7) |  |
| Very often | 134 (34.4) | 318 (66.7) | 27 (4.9) | 555 (58.9) |  |
| **33** (F016b). Recommendations for Friends, Family before forest visits: n (%) |  |  |  |  |  |
| Never | 5 (1.3) | 143 (29.8) | 34 (6.2) | 33 (3.5) | Χ² (12) = 532.3026 |
| Rarely | 33 (8.5) | 146 (30.4) | 102 (18.6) | 189 (20) | p-value  < 2.2e-16 |
| Sometimes | 137 (35.2) | 126 (26.2) | 239 (43.7) | 418 (44.3) | n = 2359 |
| Often | 131 (33.7) | 50 (10.4) | 137 (25.0) | 245 (26.0) |  |
| Very often / always | 83 (21.3) | 15 (3.1) | 35 (6.4) | 58 (6.2) |  |
| **34** (F016c). Printed map usage before forest visits: n (%) |  |  |  |  |  |
| Never | 17 (4.4) | 359 (74.8) | 265 (48.4) | 354 (37.8) |  |
| Rarely | 55 (14.2) | 87 (18.1) | 177 (32.3) | 357 (38.1) | p-value = 9.99e-08 |
| Sometimes | 141 (36.5) | 29 (6.0) | 81 (14.8) | 184 (19.6) | n = 2351 |
| Often | 113 (29.3) | 4 (0.8) | 22 (4.0) | 39 (4.2) |  |
| Very often / Always | 60 (15.5) | 1 (0.2) | 3 (0.5) | 3 (0.3) |  |
| **35** (F016d). Digital Maps Usage before forest visits: n (%) |  |  |  |  |  |
| Never | 19 (4.9) | 353 (73.4) | 50 (9.1) | 153 (16.3) | p-value = 9.99e-08 |
| Rarely | 44 (11.3) | 79 (16.4) | 80 (14.5) | 291 (31.0) | n = 2360 |
| Sometimes | 107 (27.5) | 44 (9.1) | 173 (31.3) | 323 (34.3) |  |
| Often | 128 (32.9) | 4 (0.8) | 160 (29.0) | 142 (15.1) |  |
| Very often / Always | 91 (23.4) | 1 (0.2) | 89 (16.1) | 29 (3.1) |  |
| **36** (F016e). Internet Research Usage before forest visits n (%) |  |  |  |  |  |
| Never | 6 (1.5) | 288 (59.8) | 36 (6.5) | 96 (10.3) | p-value = 9.99e-08 |
| Rarely | 30 (7.7) | 118 (24.5) | 53 (9.6) | 245 (26.2) | n = 2356 |
| Sometimes | 118 (30.3) | 59 (12.2) | 162 (29.4) | 393 (42.1) |  |
| Often | 130 (33.4) | 13 (2.7) | 204 (37.0) | 160 (17.1) |  |
| Very often / Always | 105 (27.0) | 4 (0.8) | 96 (17.4) | 40 (4.3) |  |
| **37** (F016f). Tourist Info Usage before forest visits n (%) |  |  |  |  |  |
| Never | 27 (6.9) | 445 (92.9) | 385 (71.6) | 668 (72.7) | p-value = 9.99e-08 |
| Rarely | 75 (19.3) | 25 (5.2) | 124 (23.0) | 204 (22.2) | n = 2325 |
| Sometimes | 143 (36.8) | 7 (1.5) | 28 (5.2) | 42 (4.6) |  |
| Often | 85 (21.9) | 2 (0.4) | 1 (0.2) | 5 (0.5) |  |
| Very often / Always | 59 (15.2) | 0 (0.0) | 0 (0.0) | 0 (0.0) |  |
| **38** (F016g): Journalistic reports before forest visits n (%) |  |  |  |  |  |
| Never | 21 (5.5) | 393 (81.7) | 258 (47.6) | 378 (40.9) |  |
| Rarely | 68 (17.7) | 58 (12.1) | 205 (37.8) | 334 (36.1) | p-value = 9.99e-08 |
| Sometimes | 167 (43.4) | 30 (6.2) | 73 (13.5) | 191 (20.6) | n = 2333 |
| Often | 83 (21.6) | 0 (0.0) | 5 (0.9) | 18 (1.9) |  |
| Very often/Always | 46 (11.9) | 0 (0.0) | 1 (0.2) | 4 (0.4) |  |
|  |  |  |  |  |  |
|  |  |  |  |  |  |
| **39** (F019a). Own knowledge usage during forest visits |  |  |  |  |  |
| Never | 6 (1.6) | 14 (3.0) | 13 (2.4) | 0 (0.0) | p-value = 9.99e-08 |
| Rarely | 14 (4.3) | 11 (2.3) | 46 (8.4) | 20 (2.2) | n = 2311 |
| Sometimes | 82 (21.8) | 26 (5.5) | 239 (43.7) | 30 (3.3) |  |
| Often | 156 (41.5) | 109 (23.0) | 213 (38.9) | 352 (38.5) |  |
| Very often/Always | 116 (30.9) | 314 (66.2) | 36 (6.6) | 512 (56.0) |  |
|  |  |  |  |  |  |
| **40** (F019b). Recommendations from friends and family during forest visits |  |  |  |  |  |
| Never | 8 (21) | 167 (35.5) | 83 (15.5) | 42 (4.7) | Χ² (12) = 430.104 |
| Rarely | 43 (11.5) | 102 (21.7) | 107 (19.9) | 208 (23.3) | p-value < 2.2e-16 |
| Sometimes | 129 (34.5) | 133 (28.2) | 229 (42.6%) | 341 (38.2) | n = 2274 |
| Often | 128 (34.2) | 48 (10.2) | 97 (18.1%) | 239 (26.8) |  |
| Very often/Always | 66 (17.6) | 21 (4.5) | 21 (3.9%) | 62 (7.0) |  |
|  |  |  |  |  |  |
| **41** (F019c). Signage usage during forest visits |  |  |  |  |  |
| Never | 5 (1.3) | 68 (14.4) | 10 (1.8) | 2 (0.2) | p-value = 1e-07 |
| Rarely | 16 (4.3) | 53 (11.3) | 24 (4.4) | 34 (3.8) | n = 2293 |
| Sometimes | 102 (27.3) | 142 (30.1) | 117 (21.4) | 196 (21.7) |  |
| Often | 118 (31.6) | 135 (28.7) | 228 (41.8) | 337 (37.3) |  |
| Very often/Always | 132 (35.4) | 73 (15.5) | 167 (30.6) | 334 (37.0) |  |
|  |  |  |  |  |  |
| **42** (F019d) Printed map usage during forest visits |  |  |  |  |  |
| Never | 10 (2.7) | 367 (78.1) | 234 (42.9) | 256 (28.4) | p-value = 1e-07 |
| Rarely | 64 (17.4) | 56 (11.9) | 156 (28.6) | 339 (37.6) | n = 2285 |
| Sometimes | 124 (33.7) | 42 (8.9) | 114 (20.9) | 216 (24.0) |  |
| Often | 99 (26.9) | 4 (0.9) | 32 (5.9) | 75 (8.3) |  |
| Very often/Always | 71 (19.3) | 1 (0.2) | 10 (1.8) | 15 (1.7) |  |
|  |  |  |  |  |  |
| **43** (F019e). Digital maps / Apps for smartphone, tablet, and smartwatch usage during forest visits |  |  |  |  |  |
| Never | 14 (3.8) | 349 (73.6) | 16 (3.0) | 53 (5.9) | p-value = 1e-07 |
| Rarely | 36 (9.7) | 51 (10.8) | 74 (13.7) | 254 (28.2) | n = 2289 |
| Sometimes | 100 (27.0) | 65 (13.7) | 148 (27.3) | 344 (38.1) |  |
| Often | 119 (32.1) | 7 (1.5) | 183 (33.8) | 193 (21.4) |  |
| Very often/Always | 102 (27.5) | 2 (0.4) | 121 (22.3) | 58 (6.4) |  |
|  |  |  |  |  |  |
| **44** (F019f). Mobile Internet Search during forest visits |  |  |  |  |  |
| Never | 13 (3.5) | 381 (80.7) | 20 (3.7) | 105 (11.8) | p-value = 1e-07 |
| Rarely | 36 (9.8) | 41 (8.7) | 96 (17.8) | 284 (31.8) | n = 2274 |
| Sometimes | 135 (36.6) | 47 (10.0) | 180 (33.3) | 312 (34.9) |  |
| Often | 111 (30.1) | 1 (0.2) | 180 (33.3) | 142 (15.9) |  |
| Very often/Always | 74 (20.1) | 2 (0.4) | 64 (11.9) | 50 (5.6) |  |
|  |  |  |  |  |  |
|  |  |  |  |  |  |
| **45** (F053). Self-reported digital competence |  |  |  |  |  |
| Not at all | 5 (1.4) | 9 (2.1) | 0 (0.0) | 20 (1.7) | p-value = 1e-07 |
| Basic | 74 (21.1) | 151 (35.7) | 95 (19.1) | 265 (22.1) | n = 2472 |
| Advanced | 144 (41.0) | 180 (42.6) | 238 (47.9) | 565 (47.0) |  |
| Very advanced | 128 (36.5) | 83 (19.6) | 164 (33.0) | 351 (29.2) |  |

1. *In case number of cases in the cross-table is larger than 5: Chi-square test of independence.*
2. *In case number of cases in the cross-table is smaller than 5: Fisher’s Exact Test (with simulated p-value based on 1e+07 replicates; two-sided).*

| 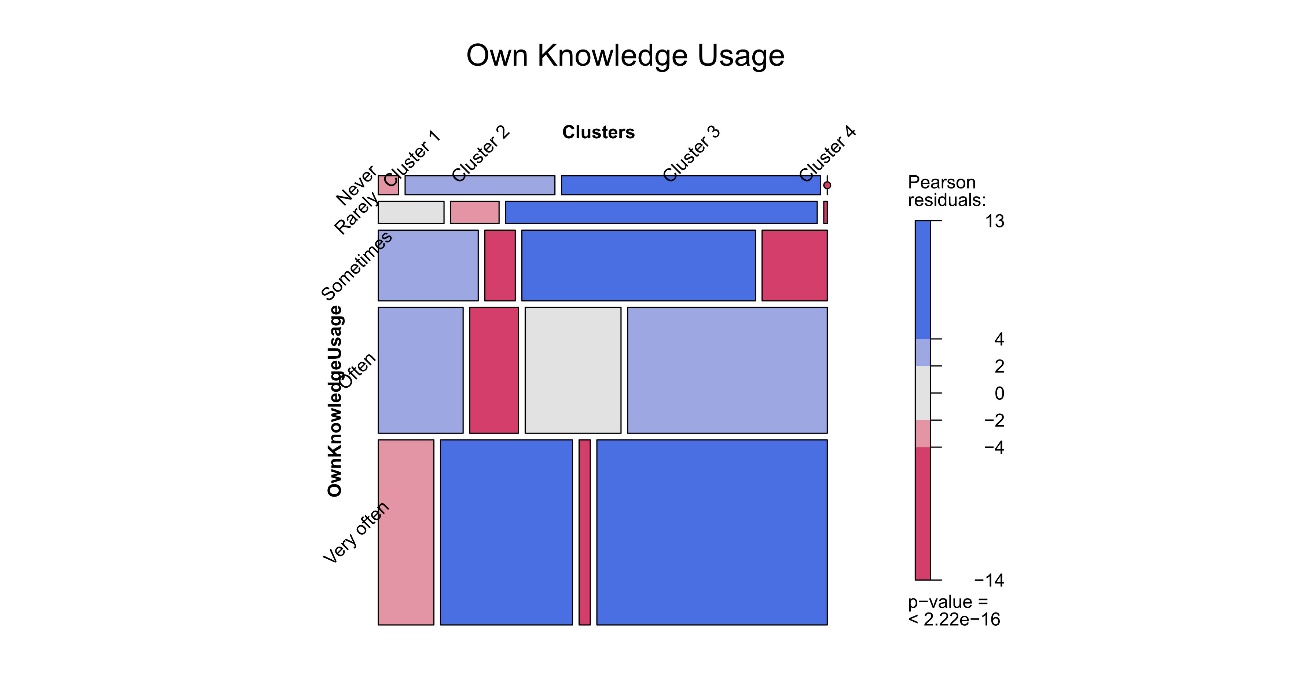 | 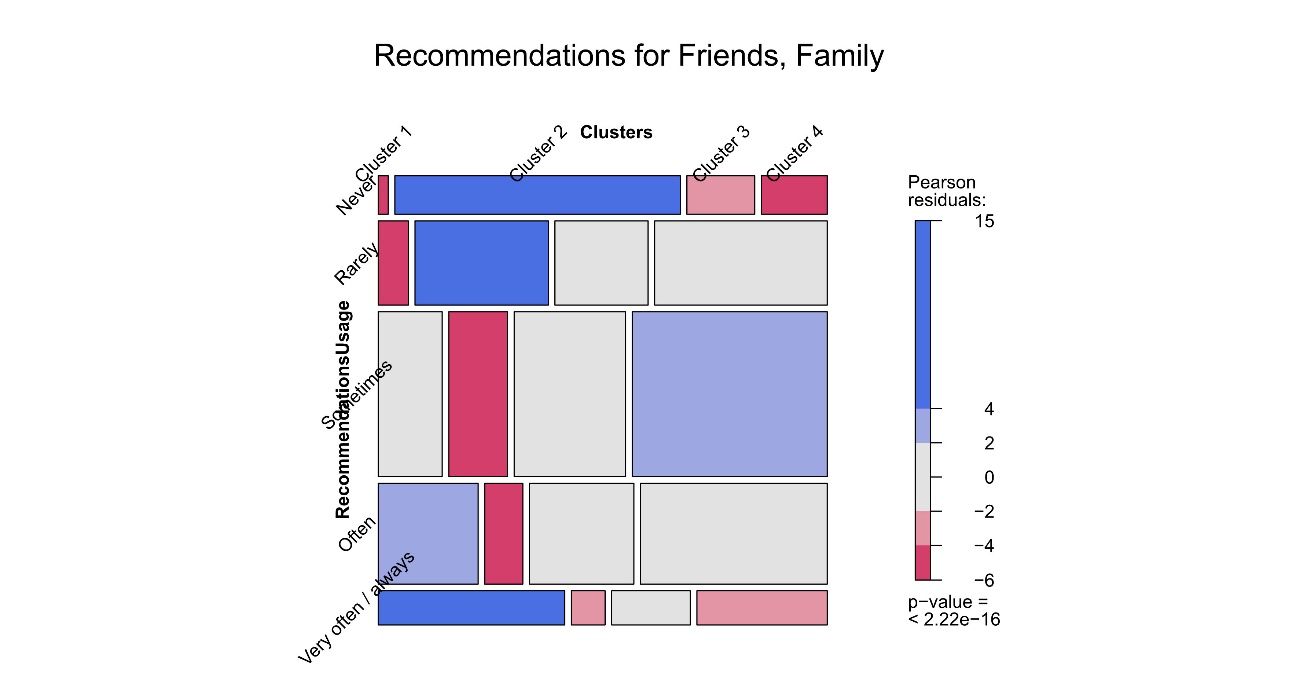 | |
| --- | --- | --- |
| **Figure S32**. Own knowledge use before forest visits (F016a)  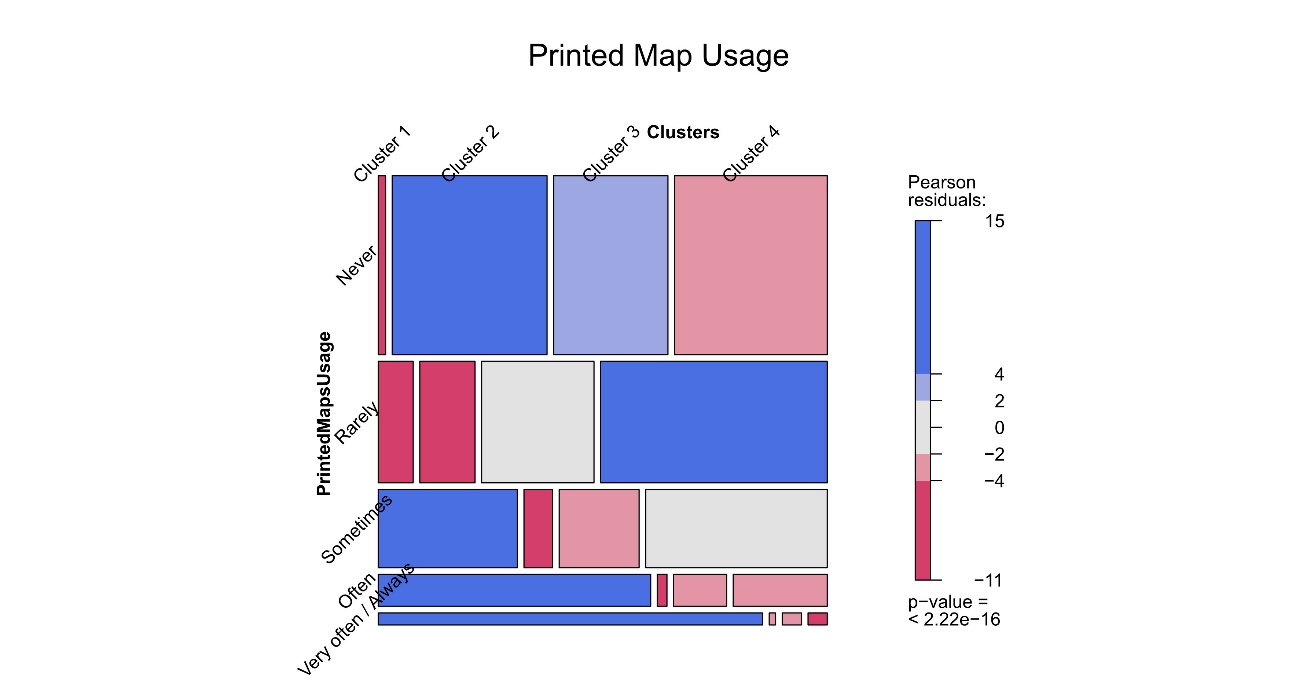  **Figure S34**. Printed Maps (F016c)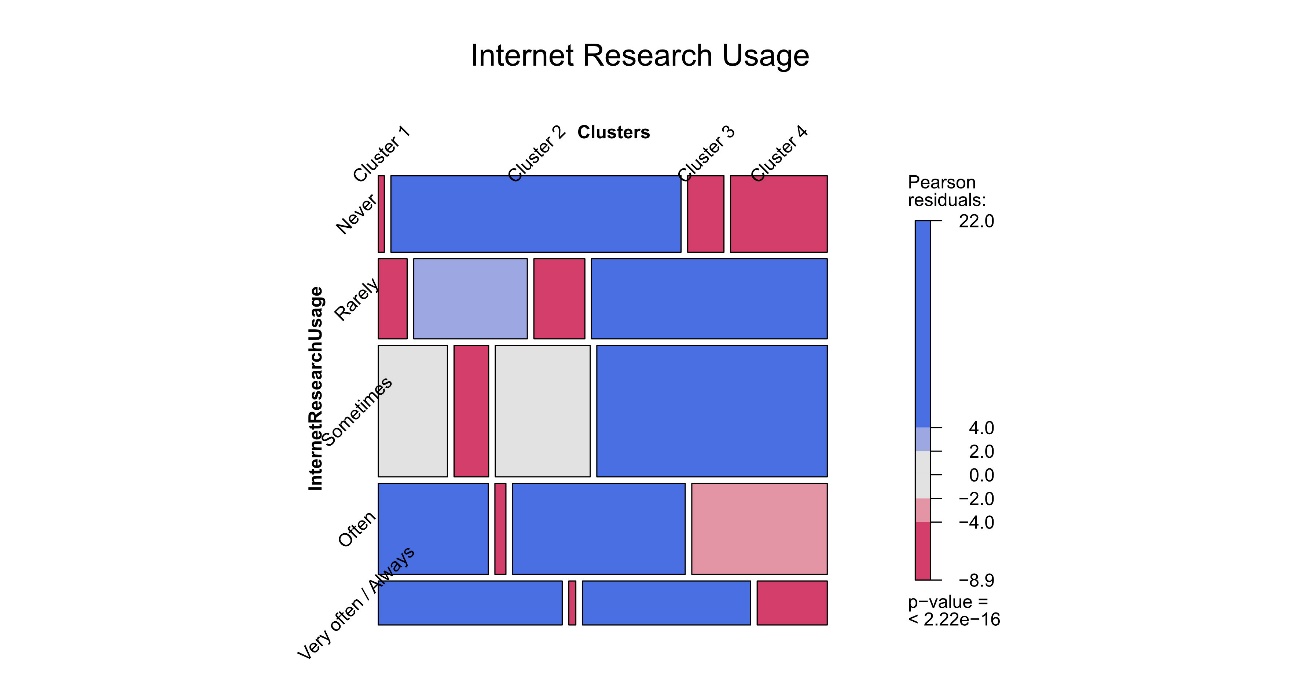  **Figure S36**. Internet Search Usage (F016e) | **Figure S33**. Recommendations from Friends/Family (F016b)  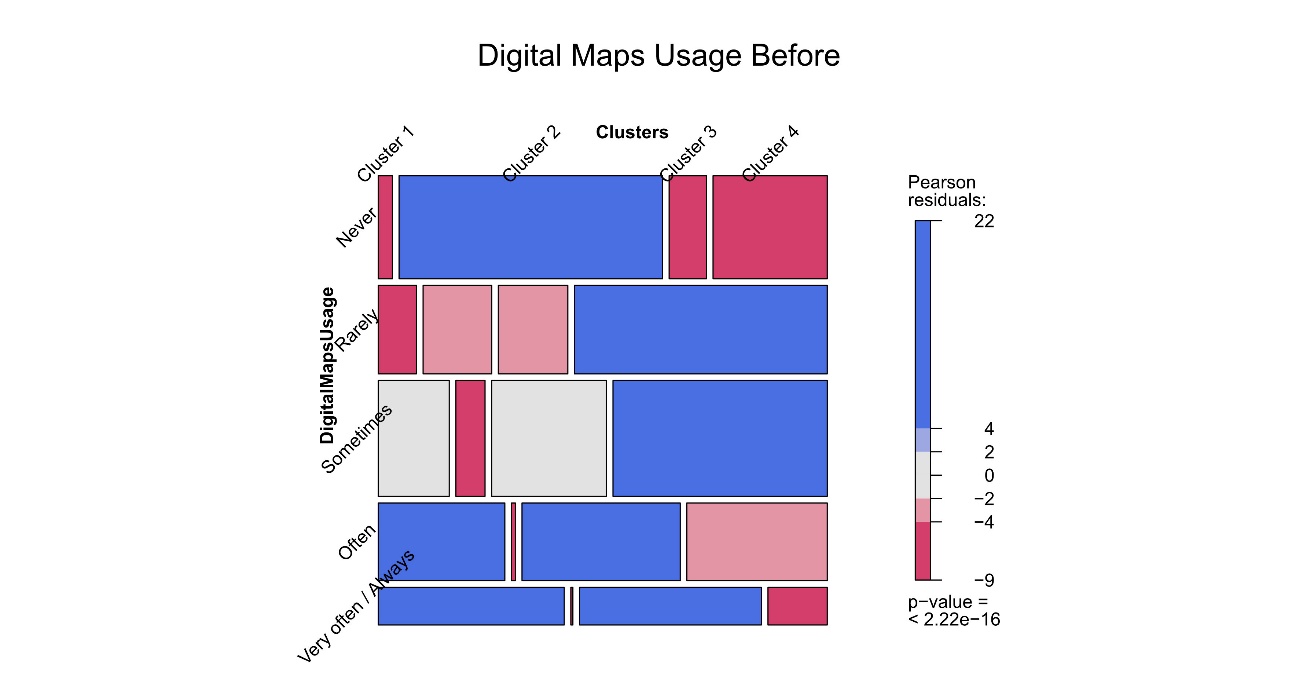  **Figure S35**. Digital Maps Usage (F016d)  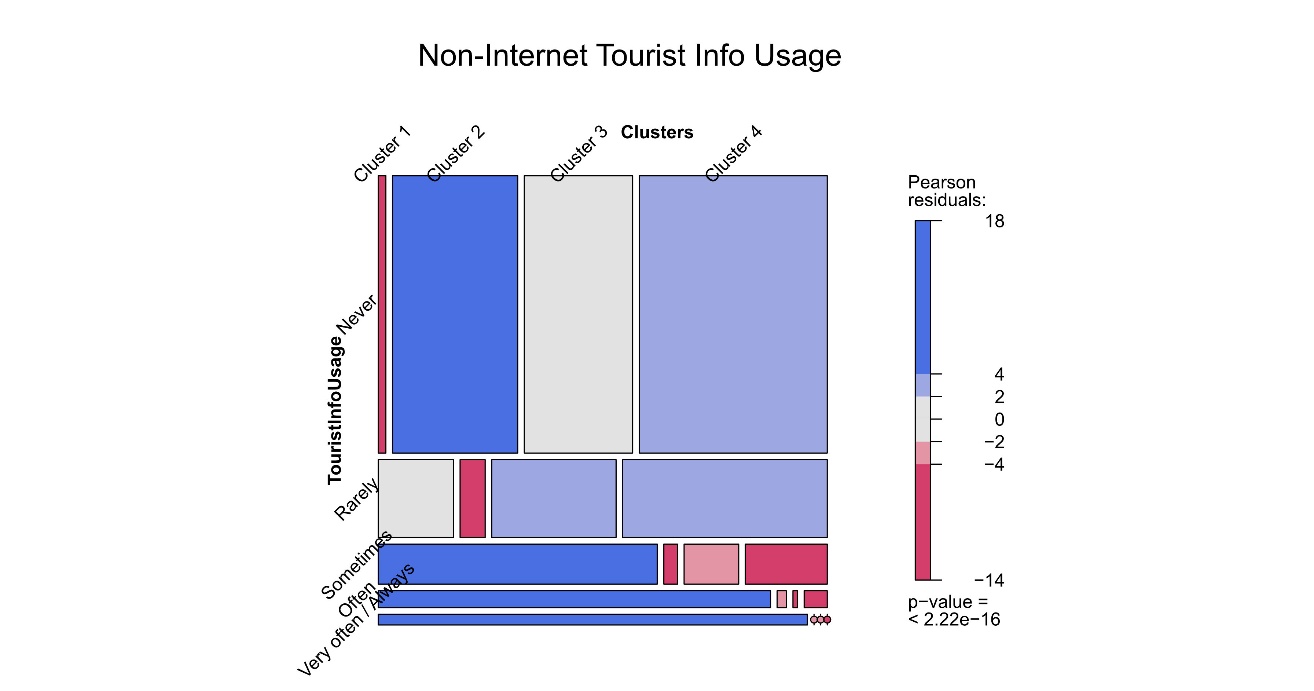  **Figure S37**. Non-Internet Tourist Info Usage (F016f) | |
| 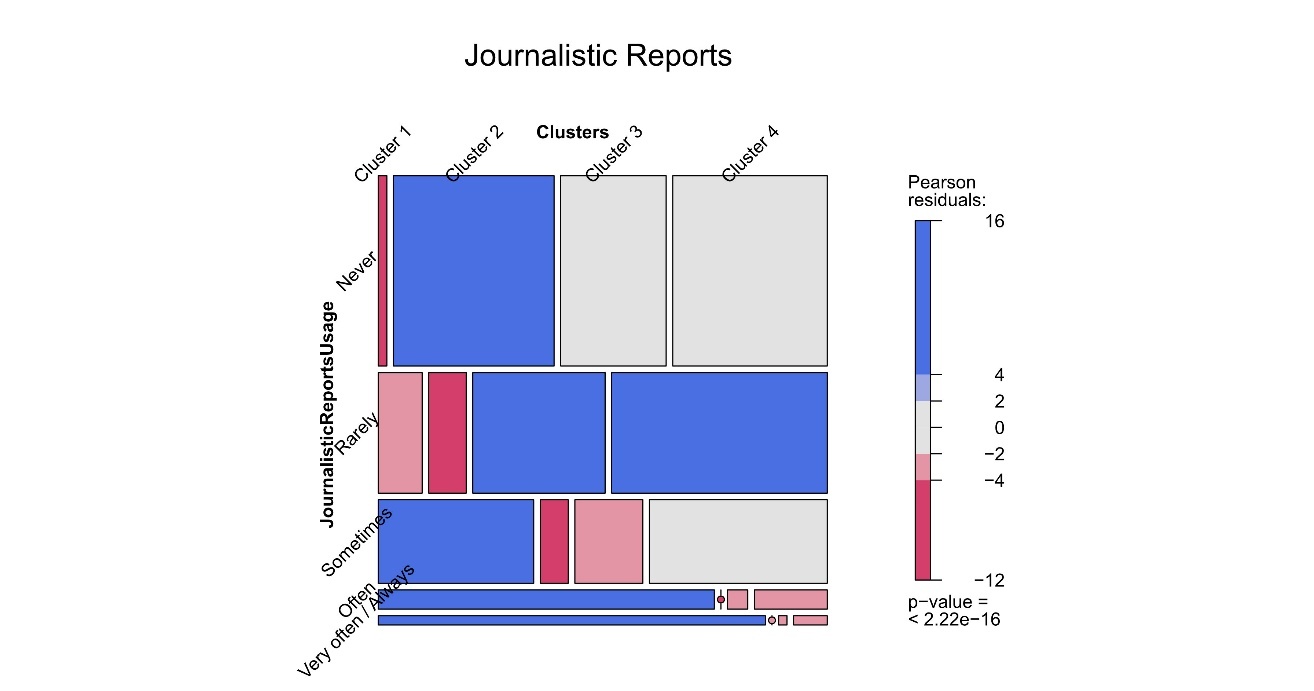 | 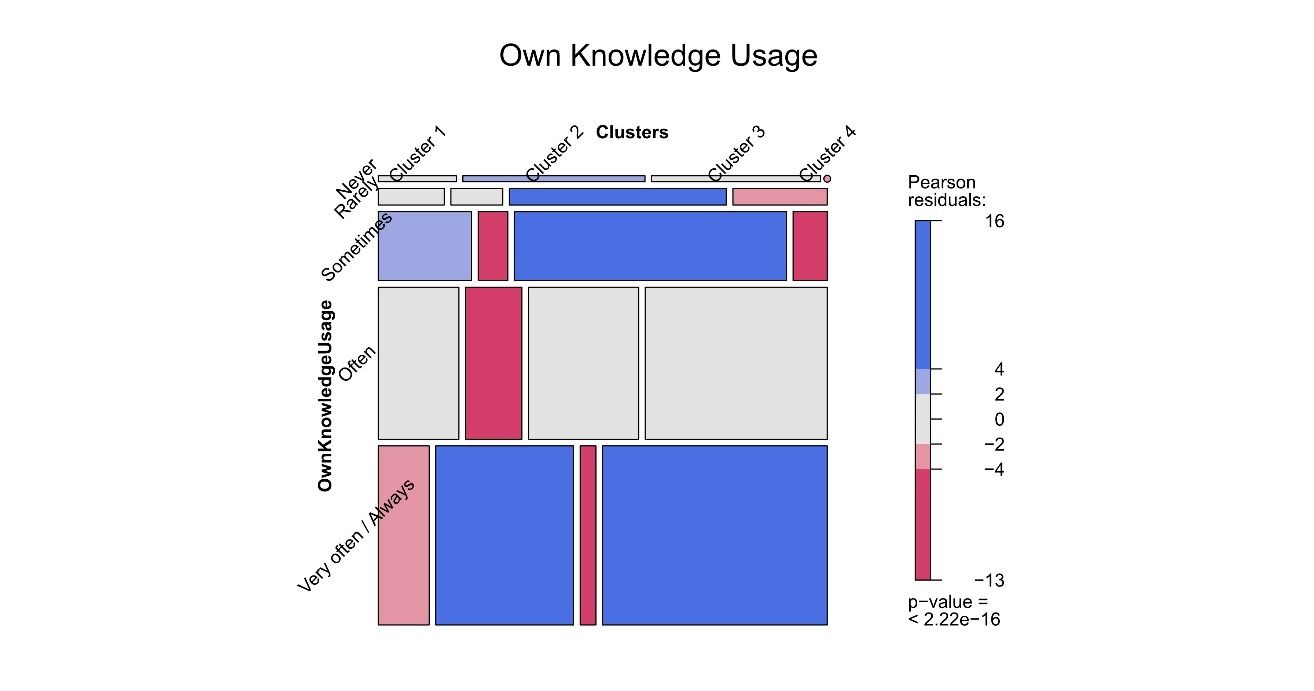 | |
| **Figure S38**. Journalistic Reports Usage (F016g) *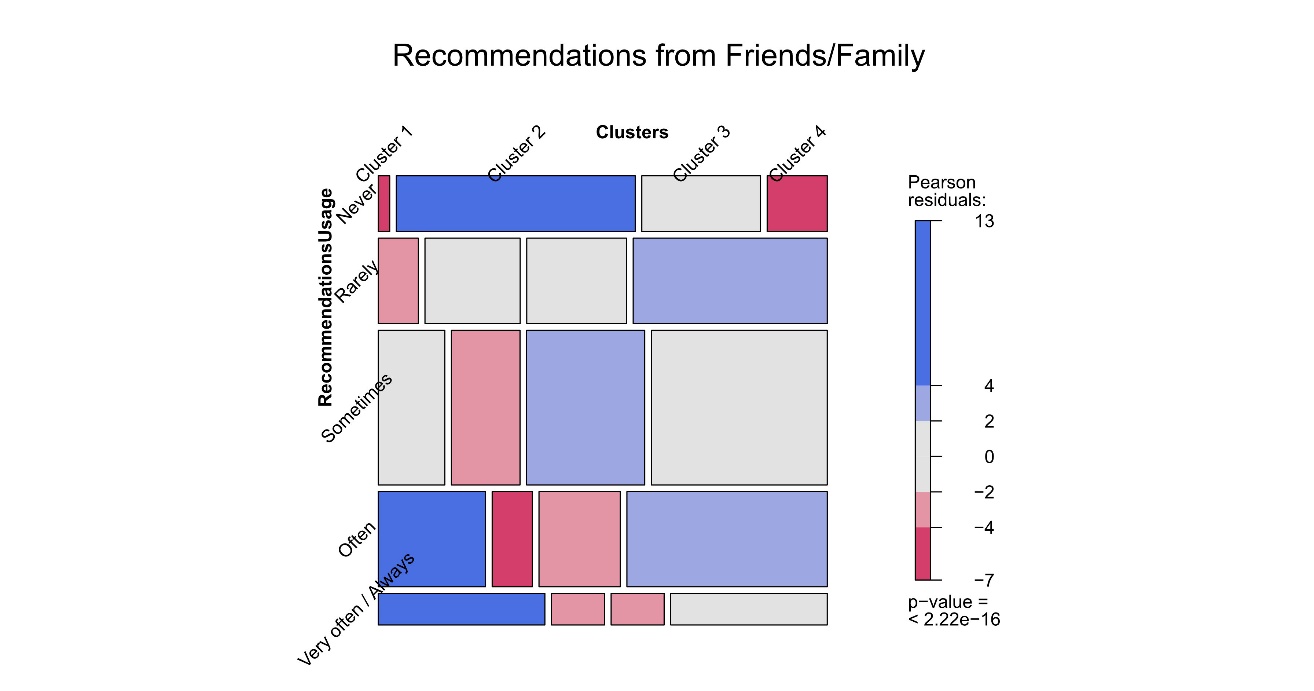* | **Figure S39**. Own Knowledge Usage during forest visits (F019a)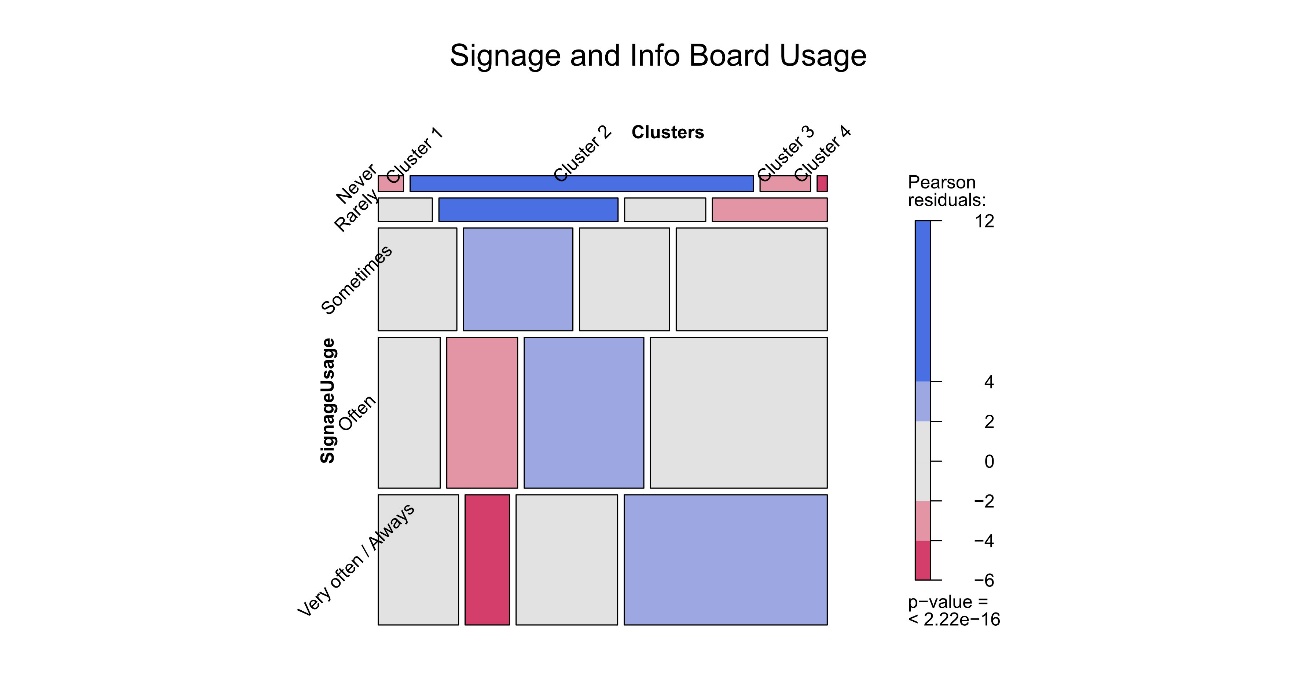 | |
| **Figure S40**. Recommendations from Friends/Family Usage (F019b) **Figure S41**. Signage Usage (F019c) | | |
| 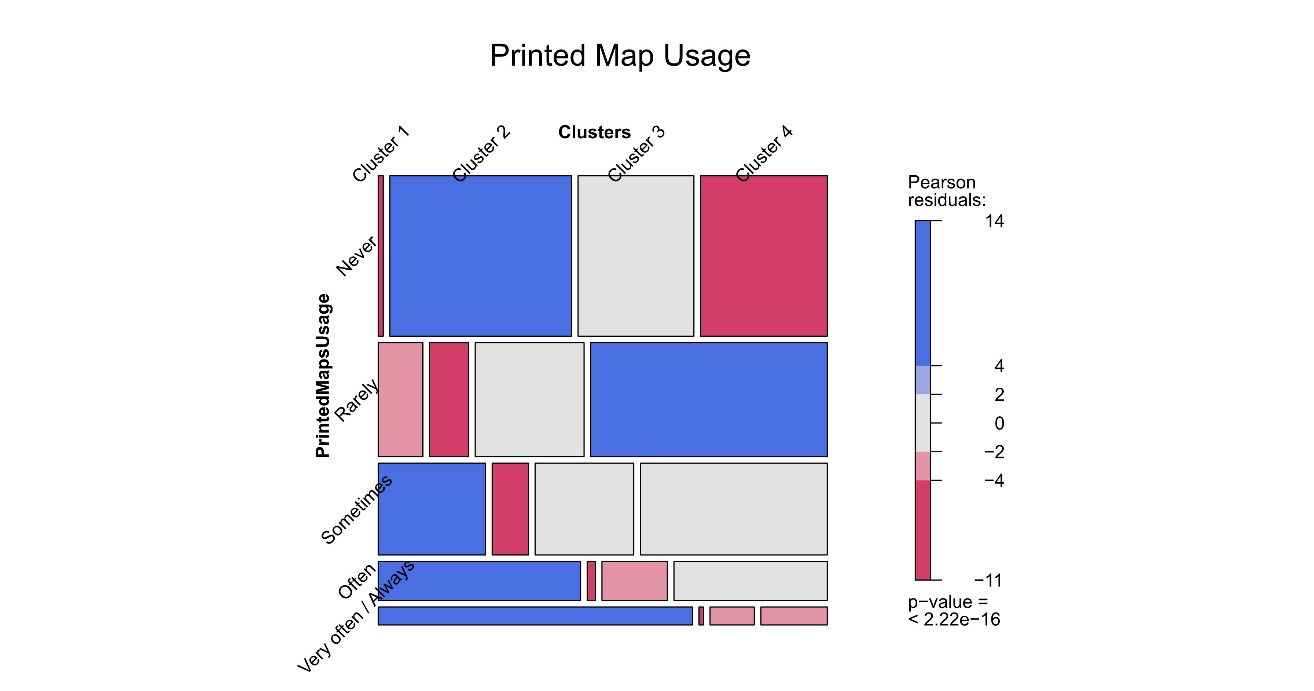 | | 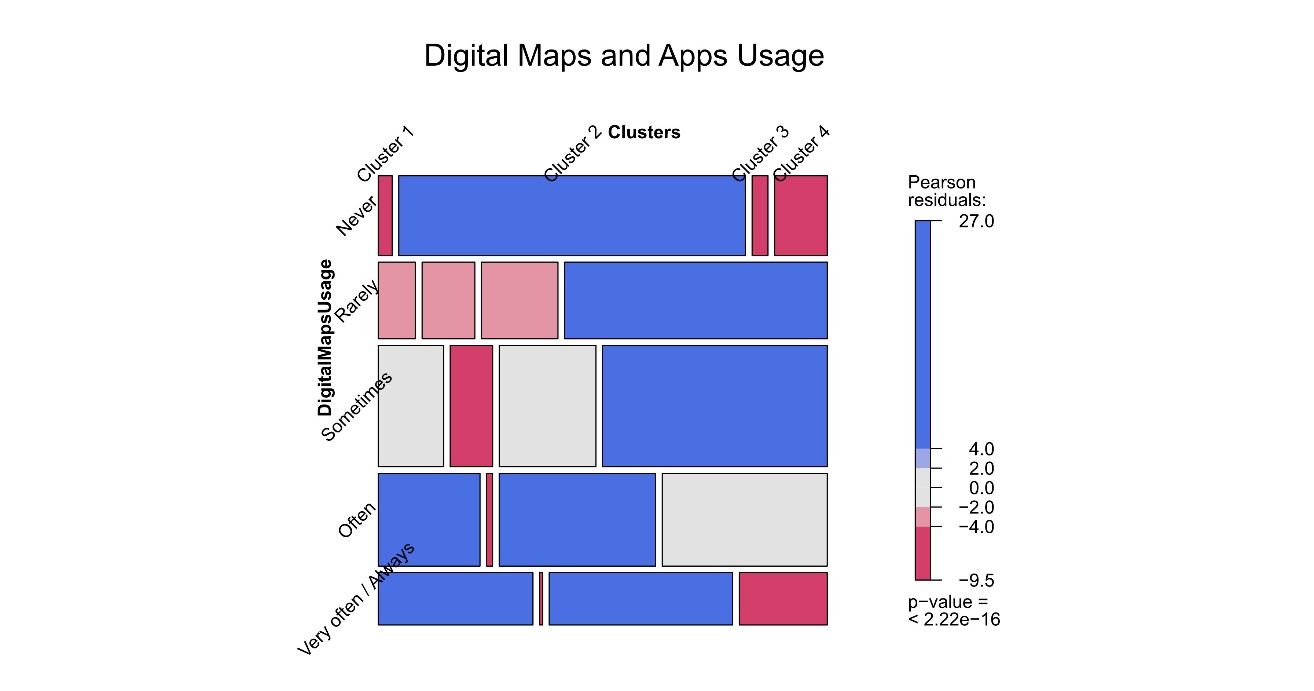 |
| **Figure S42**. Printed Maps Usage (F019d) | | **Figure S43**. Digital Maps and Apps Usage (F019e) |
|  | |  |
| 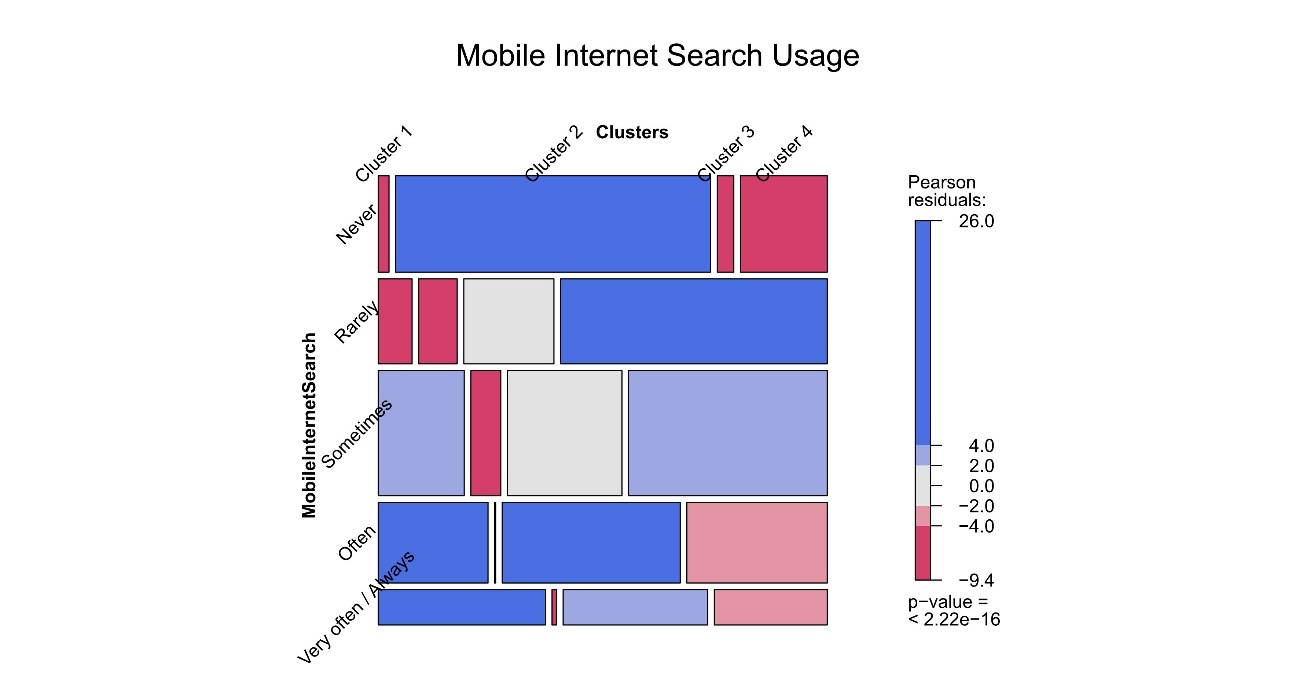  **Figure S44**. Mobile Internet Research Usage (F019f) | | 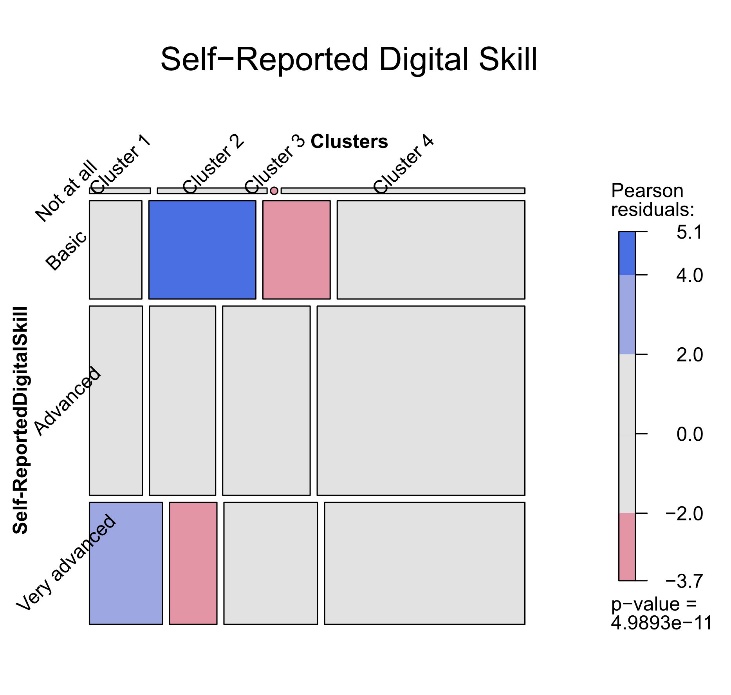  **Figure S45**. Self-reported digital skill (F053) |
