# Supplementary material for: Understanding generational differences in digital skills and recreational behaviour for effective visitor management in forest destinations
Source: Sci Rep. 2025 May 23;15:17887. doi: 10.1038/s41598-025-02036-5 (PMC12098668; doi:10.1038/s41598-025-02036-5)
Supplement: Supplementary file 3 — Supplementary Material 3 [file 41598_2025_2036_MOESM3_ESM.docx]

**Supplementary Information (S2)**

**Generational Trends in Forest Visitation, Planning Behaviour, and Digital Tool Use**

***Table S3****. Comparative analysis of generational trends in forest visitation, changes in visit frequency before and during COVID-19, and* digital tool use in the Vienna Metropolitan Area.

Each row presents frequencies and percentages by generation (Gen Z, Millennials, Gen X, Baby Boomers, Traditionalists) for survey items related to visitation, planning behaviour, digital competence, and tool use before, during, and after forest visits. SPSS variable names (e.g. F002) are shown in parentheses for reference to the original questionnaire (Table S1, Supplementary Information S1). For each row, the corresponding mosaic plot figure is numbered consistently (e.g. Figure S1–S31).

Figures S1–S31 provide a visual summary of generational patterns in forest visitation, planning behaviour, digital competence, navigation tool use, and information sharing. These mosaic plots illustrate statistically significant relationships across age groups, covering topics such as visit frequency (Figures S1–S3), planning strategies and tools (Figures S4–S12), digital tool use (Figures S13–S14), navigation behaviour (Figures S15–S22), and post-visit digital engagement (Figures S23–S31).

Table S3 provides information on whether there are significant differences between groups or not. To recognize categories that differ most, we **include** **MOSAIC PLOTS using residual-based shadings (Pearson residuals).** The colours represent the level of the residual for that cell/combination of levels. The legend is presented on the right. Blue shading means there are more observations in that cell than would be expected under the null model (independence). Red means there are fewer observations than would have been expected. Grey cells indicate that the corresponding Pearson residual is within a 95% confidence interval around zero (independence). Note that p-values in the tables have not been corrected for multiple testing since most significant p-values are much smaller than any of the usually used significance levels (0.05, 0.01, etc.).

|  | Generation Z  n (%) | Generation Y  n (%) | | Generation X  n (%) | Baby Boomers  n (%) | Traditionalists  n (%) | | Statistical test ^a,b^ | |
| --- | --- | --- | --- | --- | --- | --- | --- | --- | --- |
| **1** (F002). Frequency of forest visits n (%) |  |  | |  |  |  | |  | |
| Rarely | 93 (49) | 158 (84) | | 172 (91) | 180 (95) | 20 (11) | | p-value = 0.08258 | |
| Monthly | 26 (14) | 38 (20) | | 45 (24) | 30 (16) | 4 (2) | | n = 989 | |
| Weekly | 26 (14) | 38 (20) | | 61 (32) | 47 (25) | 5 (3) | |  | |
| Several times a week | 10 (5) | 8 (4) | | 7 (4) | 5 (3) | 1 (1) | |  | |
| Daily | 19 (10) | 27 (14) | | 54 (28) | 56 (29) | 7 (4) | |  | |
|  |  |  | |  |  |  | |  | |
| **2** (F005). Change of frequency of the forest visit change compared to BEFORE the COVID-19 pandemic n (%) |  | |  | | |  |  | |  |
| Never visited before the pandemic | 17 (10.2) | 12 (4.8) | | 32 (10.1) | 37 (12.3) | 3 (9.7) | | p = 9.99e-08 | |
| Never visited during the pandemic, but visited before | 6 (3.7) | 2 (0.5) | | 7 (1.2) | 10 (2.1) | 2 (2.0) | | n = 1062 | |
| Visited less often during the pandemic | 18 (10.8) | 19 (7.6) | | 33 (10.4) | 42 (14.0) | 8 (25.8) | |  | |
| Unchanged during the pandemic | 98 (59.0) | 166 (66.7) | | 218 (69.0) | 196 (65.3) | 18 (58.1) | |  | |
| Visited more often during the pandemic | 27 (16.3) | 50 (20.1) | | 26 (8.2) | 15 (5.0) | 0 (0.0) | |  | |
|  |  |  | |  |  |  | |  | |
| **3** (F006). Change of frequency of visits to the forests DURING the COVID-19 pandemic (2020-2021) compared to BEFORE the pandemic n (%) |  |  | |  |  |  | |  | |
| Never visited before the pandemic | 24 (6.9) | 20 (3.5) | | 44 (6.4) | 38 (5.7) | 3 (3.1) | | p-value = 9.99e-08 | |
| Never visited during the pandemic, but visited before | 32 (9.1) | 21 (3.7) | | 24 (3.5) | 32 (4.8) | 16 (16.5) | | n = 2371 | |
| Visited less often during the pandemic | 57 (16.3) | 81 (14.2) | | 90 (13.1) | 97 (14.6) | 20 (20.6) | |  | |
| Unchanged during the pandemic | 114 (32.6) | 224 (39.2) | | 340 (49.5) | 348 (52.3) | 43 (44.3) | |  | |
| Visited more often during the pandemic | 123 (35.1) | 226 (39.5) | | 189 (27.5) | 150 (22.6) | 15 (15.5) | |  | |
|  |  |  | |  |  |  | |  | |
| **4** (F015). Planning timing before forest visits n (%) |  |  | |  |  |  | |  | |
| Not at all | 22 (10.1) | 46 (10.9) | | 91 (18.3) | 145 (27.6) | 21 (33.0) | | Χ^2^ (20) = 118.955 | |
| Varies by trip | 37 (17.0) | 60 (14.6) | | 58 (11.3) | 69 (13.1) | 6 (10.0) | | p = 4.450066e-16 | |
| About a week in advance | 26 (12.2) | 23 (5.4) | | 18 (3.4) | 11 (2.1) | 5 (9.6) | | n = 2139 | |
| Several days in advance | 74 (34.9) | 109 (26.3) | | 107 (21.1) | 75 (14.3) | 13 (21.0) | |  | |
| The day before | 89 (17.1) | 155 (36.6) | | 183 (36.6) | 140 (27.6) | 25 (39.0) | |  | |
| The day of the trip | 66 (25.2) | 142 (34.5) | | 149 (28.6) | 152 (29.2) | 22 (38.0) | |  | |
|  |  |  | |  |  |  | |  | |
| **5** (F016a). Own knowledge usage before forest visits n (%) |  |  | |  |  |  | |  | |
| Never | 16 (5.5) | 29 (5.0) | | 22 (3.2) | 19 (2.9) | 3 (3.4) | | p-value = 3.7e-06 | |
| Rarely | 30 (10.3) | 28 (4.8) | | 22 (3.2) | 17 (2.6) | 5 (5.6) | | n = 2110 | |
| Sometimes | 68 (23.3) | 100 (17.3) | | 93 (13.4) | 80 (12.0) | 13 (14.6) | |  | |
| Often | 84 (28.8) | 169 (29.2) | | 170 (24.4) | 193 (28.9) | 26 (29.2) | |  | |
| Very often | 109 (37.4) | 198 (34.2) | | 294 (42.2) | 279 (41.8) | 43 (48.3) | |  | |
| **6** (F016b). Recommendations for Friends, Family before forest visits: n (%) |  |  | |  |  |  | |  | |
| Never | 16 (5.3) | 19 (3.0) | | 57 (8.2) | 81 (12.1) | 13 (14.4) | |  | |
| Rarely | 39 (12.8) | 76 (11.9) | | 122 (17.5) | 154 (23.0) | 32 (35.6) | | p-value < 2.2e-16 | |
| Sometimes | 94 (30.8) | 231 (36.2) | | 243 (34.9) | 234 (35.0) | 21 (23.3) | | n = 2111 | |
| Often | 113 (37.0) | 147 (23.0) | | 140 (20.1) | 94 (14.1) | 19 (21.1) | |  | |
| Very often / always | 46 (15.1) | 55 (8.6) | | 35 (5.0) | 23 (3.4) | 7 (7.8) | |  | |
| **7** (F016c). Printed map usage before forest visits: n (%) |  |  | |  |  |  | |  | |
| Never | 161 (52.5) | 235 (39.6) | | 245 (35.5) | 217 (32.7) | 36 (37.9) | |  | |
| Rarely | 76 (24.8) | 149 (25.1) | | 182 (26.4) | 186 (28.0) | 19 (20.0) | | p = 0.0001576 | |
| Sometimes | 40 (13.0) | 84 (14.1) | | 117 (17.0) | 126 (19.0) | 17 (17.9) | | n = 2105 | |
| Often | 25 (8.1) | 34 (5.7) | | 40 (5.8) | 48 (7.2) | 12 (12.6) | |  | |
| Very often / Always | 7 (2.3) | 22 (3.7) | | 10 (1.4) | 12 (1.8) | 5 (5.3) | |  | |
| **8** (F016d). Digital Maps Usage before forest visits: n (%) |  |  | |  |  |  | |  | |
| Never | 38 (12.5) | 82 (13.4) | | 139 (20.0) | 200 (30.1) | 45 (47.4) | | p = 6.048557e-30 | |
| Rarely | 64 (21.1) | 92 (15.1) | | 118 (17.0) | 146 (22.0) | 20 (21.1) | | n = 2111 | |
| Sometimes | 88 (29.0) | 149 (24.3) | | 190 (27.4) | 140 (21.0) | 11 (11.6) | |  | |
| Often | 68 (22.4) | 139 (22.7) | | 107 (15.4) | 77 (11.6) | 12 (12.6) | |  | |
| Very often / Always | 51 (16.8) | 63 (10.3) | | 45 (6.5) | 23 (3.5) | 4 (4.2) | |  | |
| **9** (F016e). Internet Research Usage before forest visits n (%) |  |  | |  |  |  | |  | |
| Never | 32 (10.5) | 45 (7.1) | | 96 (13.4) | 158 (23.7) | 35 (36.8) | | p-value = 9.99e-08 | |
| Rarely | 43 (14.1) | 72 (11.4) | | 116 (16.1) | 138 (20.7) | 22 (23.2) | | n = 2110 | |
| Sometimes | 86 (28.2) | 174 (27.5) | | 202 (28.0) | 185 (27.7) | 18 (18.9) | |  | |
| Often | 86 (28.2) | 159 (25.2) | | 134 (18.6) | 76 (11.4) | 13 (13.7) | |  | |
| Very often / Always | 61 (20.0) | 78 (12.3) | | 50 (6.9) | 27 (4.0) | 4 (4.2) | |  | |
| **10** (F016). Tourist Info Usage before forest visits n (%) |  |  | |  |  |  | |  | |
| Never | 193 (63.0) | 321 (53.5) | | 400 (57.2) | 404 (61.2) | 52 (55.3) | | p-value = 0.0001304 | |
| Rarely | 60 (19.6) | 98 (16.3) | | 100 (14.3) | 106 (16.0) | 17 (18.1) | | n = 2082 | |
| Sometimes | 33 (10.8) | 50 (8.3) | | 59 (8.4) | 50 (7.6) | 8 (8.5) | |  | |
| Often | 16 (5.2) | 26 (4.3) | | 21 (3.0) | 12 (1.8) | 8 (8.5) | |  | |
| Very often / Always | 6 (2.0) | 26 (4.3) | | 7 (1.0) | 5 (0.8) | 4 (4.3) | |  | |
| **11** (F016g): Journalistic reports before forest visits n (%) |  |  | |  |  |  | |  | |
| Never | 164 (53.0) | 242 (40.3) | | 260 (37.2) | 236 (35.7) | 30 (31.6) | |  | |
| Rarely | 78 (25.2) | 155 (25.8) | | 178 (25.5) | 174 (26.4) | 21 (22.1) | | p = 0.0001218 | |
| Sometimes | 41 (13.3) | 82 (13.6) | | 121 (17.3) | 149 (22.5) | 30 (31.6) | | n = 2111 | |
| Often | 18 (5.8) | 27 (4.5) | | 23 (3.3) | 19 (2.9) | 6 (6.3) | |  | |
| Very often/Always | 7 (2.3) | 13 (2.2) | | 9 (1.3) | 6 (0.9) | 4 (4.2) | |  | |
|  |  |  | |  |  |  | |  | |
| **12** (F028). Route Planning Preferences n (%) |  |  | |  |  |  | |  | |
| Both | 63 (28.4) | 105 (24.6) | | 125 (23.8) | 121 (22.8) | 7 (10.6) | | Χ^2^ (8) = 25.208 | |
| Plan myself | 74 (33.5) | 144 (33.2) | | 143 (27.2) | 137 (24.8) | 24 (34.0) | | p = 0.001432764 | |
| Use suggestions | 94 (38.1) | 163 (42.2) | | 146 (49.1) | 106 (46.8) | 8 (55.4) | | n = 1460 | |
|  |  |  | |  |  |  | |  | |
| **13** (F020). Carrying Mobile Phone during forest visits n (%) |  |  | |  |  |  | |  | |
| Never | 4 (1.3) | 3 (0.5) | | 2 (0.3) | 1 (0.2) | 1 (1.1) | |  | |
| Rarely | 0 (0.0) | 2 (0.3) | | 2 (0.3) | 2 (0.3) | 3 (3.3) | | p-value = 7.669e-05 | |
| Sometimes | 51 (16.0) | 70 (11.5) | | 74 (10.7) | 49 (7.5) | 18 (19.6) | | n = 1460 | |
| Always | 259 (82.7) | 460 (75.6) | | 528 (76.4) | 540 (82.0) | 70 (76.1) | |  | |
|  |  |  | |  |  |  | |  | |
| **14** (F022). Digital tool usage in the forest (e.g. smart phone, mobile phone, GPS) n (%) |  |  | |  |  |  | |  | |
| No, never | 24 (11.0) | 53 (20.8) | | 94 (29.6) | 108 (34.6) | 24 (34.0) | | χ² (8) = 111.269 | |
| Yes, sometimes | 120 (55.0) | 227 (55.5) | | 266 (49.1) | 324 (46.8) | 47 (47.2) | | p = 2.087405e-20 | |
| Yes, frequently | 163 (34.0) | 247 (23.7) | | 239 (21.3) | 150 (18.6) | 16 (18.8) | | n = 2102 | |
|  |  |  | |  |  |  | |  | |
| **15** (F019a). Own knowledge usage during forest visits |  |  | |  |  |  | |  | |
| Never | 4 (1.3) | 9 (1.5) | | 5 (0.8) | 9 (1.4) | 2 (2.1) | | p-value = 3.8e-06 | |
| Rarely | 22 (7.1) | 23 (3.9) | | 18 (2.9) | 13 (2.0) | 7 (7.4) | | n = 2118 | |
| Sometimes | 70 (22.6) | 96 (16.1) | | 96 (15.2) | \| 72 (10.9) \| \| --- \|  \|  \| \| --- \| | 7 (7.4) | |  | |
| Often | 105 (33.9) | 209 (35.0) | | 209 (33.1) | 224 (33.9) | 36 (38.3) | |  | |
| Very often/Always | 108 (34.9) | 191 (32.0) | | 272 (43.1) | 271 (41.0) | 40 (42.6) | |  | |
|  |  |  | |  |  |  | |  | |
| **16** (F019b). Recommendations from friends and family during forest visits |  |  | |  |  |  | |  | |
| Never | 19 (6.1) | 50 (8.2) | | 101 (14.5) | 86 (13.1) | 11 (11.6) | | χ² (16) = 120.0797 | |
| Rarely | 51 (16.3) | 80 (13.1) | | 108 (15.5) | 153 (23.4) | 26 (27.4) | | p = 5.302399e-18 | |
| Sometimes | 98 (31.3) | 196 (32.1) | | 220 (31.7) | 228 (34.6) | 30 (31.6) | | n = 2087 | |
| Often | 98 (31.3) | 137 (22.4) | | 129 (18.6) | 94 (14.3) | 18 (18.9) | |  | |
| Very often/Always | 40 (12.8) | 59 (9.7) | | 31 (4.5) | 18 (2.7) | 6 (6.3) | |  | |
|  |  |  | |  |  |  | |  | |
| **17** (F019c). Signage usage during forest visits |  |  | |  |  |  | |  | |
| Never | 11 (3.5) | 19 (3.1) | | 20 (2.9) | 20 (3.0) | 2 (2.1) | | p-value = 0.2162 | |
| Rarely | 13 (4.2) | 21 (3.6) | | 37 (5.3) | 36 (5.4) | 8 (8.4) | | n = 2108 | |
| Sometimes | 70 (22.4) | 141 (24.3) | | \| 134 (19.0) \| \| --- \|  \|  \| \| --- \| | 142 (21.3) | 26 (27.4) | |  | |
| Often | 99 (31.6) | 198 (34.1) | | 216 (30.7) | 211 (31.7) | 34 (35.8) | |  | |
| Very often/Always | 117 (37.3) | 144 (24.8) | | 192 (27.3) | 175 (26.3) | 22 (23.2) | |  | |
|  |  |  | |  |  |  | |  | |
| **18** (F019d) Printed map usage during forest visits |  |  | |  |  |  | |  | |
| Never | 133 (42.9) | 219 (35.1) | | 230 (33.3) | 188 (28.5) | 25 (26.3) | | χ² (16) = 35.22407 | |
| Rarely | 82 (26.5) | 141 (22.6) | | 156 (22.6) | 166 (25.7) | 24 (25.3) | | p = 0.003702967 | |
| Sometimes | 50 (16.1) | 94 (15.1) | | 145 (21.0) | 152 (23.0) | 25 (26.3) | | n = 2103 | |
| Often | 30 (9.7) | 47 (7.5) | | 42 (6.1) | 56 (8.5) | 12 (12.6) | |  | |
| Very often/Always | 13 (4.2) | 26 (4.2) | | 20 (2.9) | 22 (3.4) | 5 (5.3) | |  | |
|  |  |  | |  |  |  | |  | |
| **19** (F019e). Digital maps / Apps for smartphone, tablet, and smartwatch usage during forest visits |  |  | |  |  |  | |  | |
| Never | 29 (9.4) | 49 (8.1) | | 100 (14.5) | 170 (26.2) | 33 (34.7) | | χ² (16) = 202.4417 | |
| Rarely | 46 (14.9) | 85 (14.1) | | 90 (13.1) | 129 (19.6) | 25 (26.3) | | p = 2.544478e-34 | |
| Sometimes | 80 (25.8) | 157 (26.1) | | 211 (30.6) | 155 (23.5) | 11 (11.6) | | n = 2102 | |
| Often | 98 (31.6) | 143 (23.7) | | 118 (17.1) | 98 (14.7) | 13 (13.7) | |  | |
| Very often/Always | 55 (17.7) | 95 (15.7) | | 73 (10.6) | 31 (4.8) | 8 (8.4) | |  | |
|  |  |  | |  |  |  | |  | |
| **20** (F019f). Mobile Internet Search during forest visits |  |  | |  |  |  | | χ² (16) = 199.642 | |
| Never | 43 (15.0) | 60 (15.0) | | 127 (25.0) | 199 (27.3) | 32 (47.2) | | p = 9.368575e-34 | |
| Rarely | 54 (18.1) | 86 (20.1) | | 112 (18.3) | 138 (14.2) | 24 (22.1) | | n = 2090 | |
| Sometimes | 88 (31.0) | 166 (28.1) | | 205 (27.3) | 153 (15.6) | 16 (25.1) | |  | |
| Often | 72 (19.3) | 154 (23.6) | | 108 (14.1) | 70 (9.3) | 11 (11.3) | |  | |
| Very often/Always | 47 (16.5) | 62 (19.9) | | 37 (20.6) | 19 (20.2) | 7 (14.5) | |  | |
|  |  |  | |  |  |  | |  | |
| **21** (F019g). A GPS device (not GPS via smartphone) use during forest visits |  |  | |  |  |  | |  | |
| Never | 172 (76.0) | 296 (69.6) | | 370 (73.5) | 392 (78.2) | 54 (79.4) | | χ² (16) = 52.790 | |
| Rarely | 39 (17.0) | 61 (14.3) | | 66 (13.1) | 80 (14.3) | 10 (14.4) | | p = 8.161019e-06 | |
| Sometimes | 45 (19.3) | 72 (17.1) | | 82 (17.3) | 62 (15.6) | 11 (21.1) | | n = 2082 | |
| Often | 30 (14.1) | 70 (14.3) | | 44 (11.1) | 26 (8.3) | 7 (5.3) | |  | |
| Very often/Always | 19 (8.0) | 26 (6.2) | | 31 (4.8) | 11 (3.2) | 6 (4.8) | |  | |
|  |  |  | |  |  |  | |  | |
| **22** (F019h). Printouts from the internet, digital maps usage during forest visits |  |  | |  |  |  | |  | |
| Never | 177 (57.1) | 298 (46.8) | | 329 (48.2) | 310 (47.0) | 44 (46.3) | |  | |
| Rarely | 48 (15.5) | 76 (11.9) | | 101 (14.8) | 134 (20.3) | 17 (17.9) | |  | |
| Sometimes | 45 (14.5) | 93 (14.6) | | 116 (17.0) | 92 (14.0) | 16 (16.8) | | p-value = 9.99e-07 | |
| Often | 30 (9.7) | 29 (4.6) | | 36 (5.3) | 16 (2.4) | 9 (9.5) | | n = 2065 | |
| Very often/Always | 8 (2.6) | 25 (3.9) | | 8 (1.2) | 6 (0.9) | 2 (2.1) | |  | |
|  |  |  | |  |  |  | |  | |
| **23** (F030a). Photo sharing after forest visits |  |  | |  |  |  | |  | |
| No, I don't use it | 0 (0.0) | 0 (0.0) | | 0 (0.0) | 0 (0.0) | 0 (0.0) | | p-value = 1e-07 | |
| No, I keep it private | 78 (30.1) | 124 (28.3) | | 130 (30.4) | 112 (25.3) | 13 (21.3) | | n = 1340 | |
| Yes, visible to friends/family only | 130 (50.2) | 221 (50.5) | | 249 (58.2) | 290 (65.4) | 33 (54.1) | |  | |
| Yes, publicly visible | 61 (19.7) | 92 (21.0) | | 76 (17.8) | 34 (9.3) | 7 (24.6) | |  | |
|  |  |  | |  |  |  | |  | |
| **24** (F030b). Sharing route (GPS / GNSS tracks) or finished tours after forest visits |  |  | |  |  |  | |  | |
| No, I don't use | 133 (48.6) | 237 (49.7) | | 276 (53.8) | 314 (59.6) | 29 (51.8) | | Χ2 (12) = 50.900 | |
| No, I Keep it private | 82 (30.2) | 135 (28.3) | | 124 (24.2) | 99 (18.8) | 13 (23.2) | | p = 9.700319e-07 | |
| Yes, visible to friends/family only | 60 (22.2) | 75 (15.8) | | 70 (13.6) | 47 (8.9) | 14 (22.8) | | n = 1782 | |
| Yes, publicly visible | 8 (3.5) | 22 (6.2) | | 29 (8.4) | 12 (2.7) | 3 (4.0) | |  | |
|  |  |  | |  |  |  | |  | |
| **25** (F030c). Text, Messaging/Chat Usage after forest visits |  |  | |  |  |  | |  | |
| No, I don't use it | 38 (14.3) | 101 (21.6) | | 154 (23.9) | 165 (31.2) | 20 (28.6) | | Χ2 (12) = 77.21973 | |
| No, I keep it private | 84 (31.6) | 125 (26.8) | | 86 (13.3) | 71 (13.4) | 12 (17.1) | | p = 1.395373e-11 | |
| Yes, visible to friends/family only | 139 (52.3) | 225 (48.2) | | 240 (37.2) | 230 (43.5) | 30 (42.9) | | n = 1773 | |
| Yes, publicly visible | 16 (1.8) | 11 (3.4) | | 13 (1.6) | 10 (1.9) | 3 (11.4) | |  | |
|  |  |  | |  |  |  | |  | |
| **26** (F030d). Tour Rating after forest visits. |  |  | |  |  |  | |  | |
| No, I don't use it | 165 (60.6) | 275 (66.4) | | 345 (70.1) | \| 343 (69.8) \| \| --- \|  \|  \| \| --- \| | 31 (53.4) | | p-value = 2e-07 | |
| No, I keep it private | 56 (20.5) | 72 (17.4) | | 61 (12.4) | 59 (12.0) | 16 (27.6) | | n = 1790 | |
| Yes, visible to friends/family only | 42 (15.4) | 65 (15.7) | | 39 (7.9) | 44 (8.9) | 13 (15.9) | |  | |
| Yes, publicly visible | 19 (3.5) | 57 (4.1) | | 56 (9.6) | 29 (5.3) | 3 (3.1) | |  | |
|  |  |  | |  |  |  | |  | |
| **27** (F030e). Forums, giving feedback after forest visits |  |  | |  |  |  | |  | |
| No, I don't use it | 197 (69.8) | 317 (69.5) | | 372 (72.2) | 383 (75.5) | 34 (56.7) | | p-value = 9.99e-08 | |
| No. I keep it private | 41 (14.5) | 63 (13.8) | | 50 (9.7) | 48 (9.5) | 17 (28.3) | | n = 1795 | |
| Yes, visible to friends/family only | 32 (11.3) | 30 (6.6) | | 35 (6.8) | 25 (4.9) | 11 (18.3) | |  | |
| Yes, publicly visible | 16 (4.4) | 56 (10.1) | | 45 (11.3) | 22 (10.1) | 1 (3.2) | |  | |
|  |  |  | |  |  |  | |  | |
| **28** (F030f). Reports and summaries sharing after forest visits |  |  | |  |  |  | |  | |
| No, I don't use it | 195 (68.6) | 315 (73.4) | | 378 (79.2) | 359 (76.6) | 31 (55.4) | | p-value = 1.26e-05 | |
| No. I keep it private | 44 (15.5) | 58 (13.5) | | 66 (13.8) | 71 (15.1) | 19 (33.9) | | N = 1706 | |
| Yes, visible to friends/family only | 38 (15.9) | 56 (13.1) | | 33 (7.0) | 33 (7.0) | 10 (10.7) | |  | |
| Yes, publicly visible | 0 (0.0) | 0 (0.0) | | 0 (0.0) | 0 (0.0) | 0 (0.0) | |  | |
|  |  |  | |  |  |  | |  | |
| **29** (F030j). Sharing activity parameters after forest visits |  |  | |  |  |  | |  | |
| No, I don't use it | 160 (56.7) | 286 (59.3) | | 344 (64.2) | 354 (71.1) | 32 (52.5) | | p-value = 9.99e-08 | |
| No. I keep it private | 66 (23.4) | 81 (16.8) | | 77 (14.8) | 84 (16.8) | 18 (29.5) | | n = 1790 | |
| Yes, visible to friends/family only | 42 (14.9) | 70 (14.8) | | 55 (10.9) | 32 (6.5) | 10 (16.4) | |  | |
| Yes, publicly visible | 14 (5.0) | 32 (6.8) | | 24 (5.0) | 6 (1.6) | 3 (1.6) | |  | |
|  |  |  | |  |  |  | |  | |
| **30** (F030k). Comparison of performance |  |  | |  |  |  | |  | |
| No, I don't use it | 161 (57.3) | 287 (58.3) | | 349 (64.5) | 356 (74.7) | 39 (62.9) | | p-value = 9.99e-08 | |
| No. I keep it private | 57 (20.3) | 99 (20.8) | | 94 (18.3) | 77 (16.1) | 13 (20.6) | | n = 1773 | |
| Yes, visible to friends/family only | 52 (18.5) | 62 (13.3) | | 41 (10.0) | 22 (4.5) | 9 (12.1%) | |  | |
| Yes, publicly visible | 11 (3.9) | 22 (7.5) | | 13 (7.2) | 6 (4.7) | 3 (4.4%) | |  | |
|  |  |  | |  |  |  | |  | |
| **31** (F030l). Share tips, read comments |  |  | |  |  |  | |  | |
| No, I don't use it | 171 (61.6) | 276 (59.3) | | 321 (64.6) | 292 (61.3) | 29 (51.8) | | χ2(12) =31.485 | |
| No. I keep it private | 49 (17.7) | 67 (14.4) | | 56 (11.3) | 63 (13.2) | 12 (21.4) | | p-value = 0.001661 | |
| Yes, visible to friends/family only | 43 (15.3) | 63 (13.5) | | 58 (11.7) | 71 (14.9) | 10 (17.9) | | n = 1729 | |
| Yes, publicly visible | 15 (5.4) | 57 (12.8) | | 48 (12.4) | 23 (10.6) | 5 (8.9) | |  | |

1. *In case number of cases in the cross-table is larger than 5: Chi-square test of independence*
2. *In case number of cases in the cross-table is smaller than 5: Fisher’s Exact Test (with simulated p-value based on 1e+07 replicates; two-sided)*

| 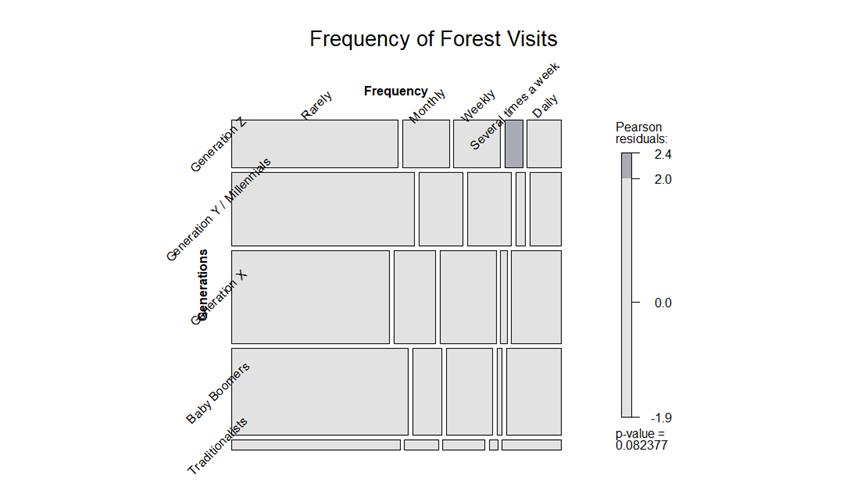 | 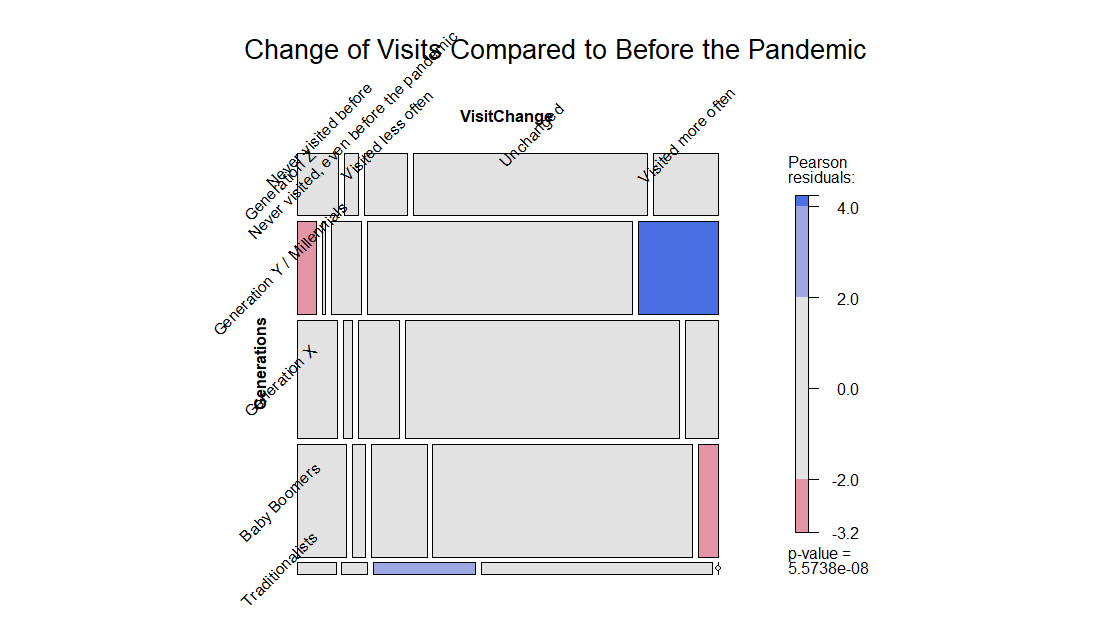 | |
| --- | --- | --- |
| **Figure S1**. Frequency of Forest Visits (F002)  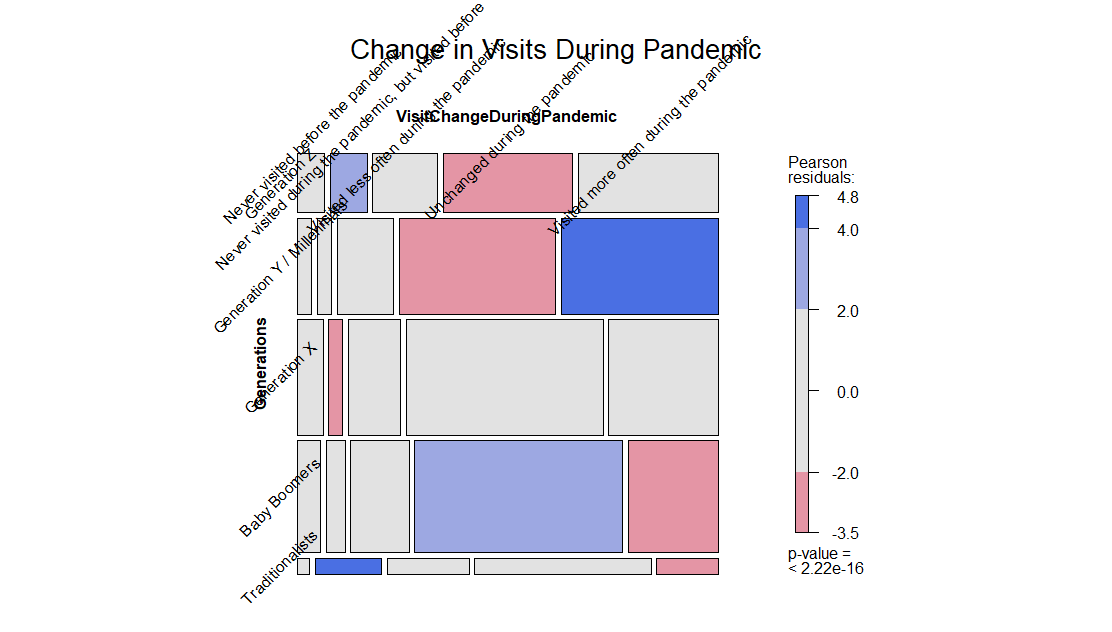  **Figure S3**. Change in Visits During the Pandemic (F006)  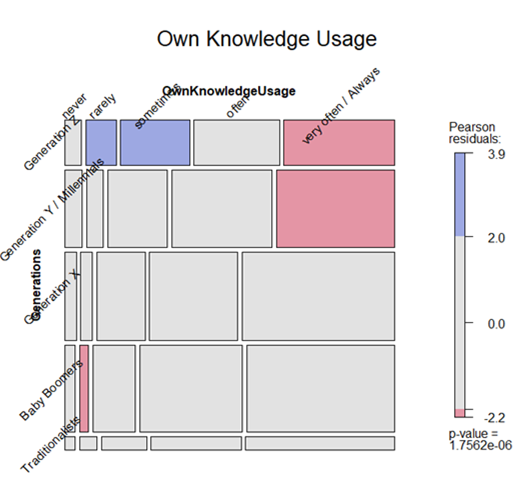  **Figure S5**. Own Knowledge Usage (F016a) | **Figure S2**. Change in Visit Frequency Compared to Before the Pandemic (F005)  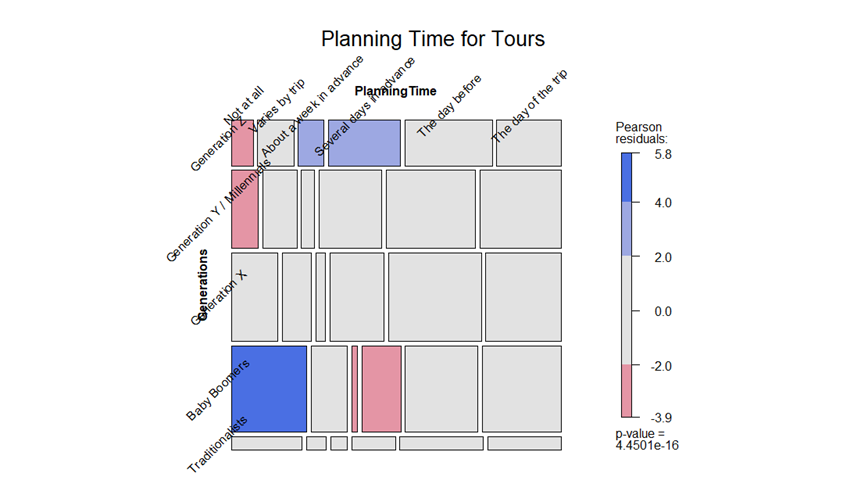  **Figure S4**. Planning Time for Tours (F015)  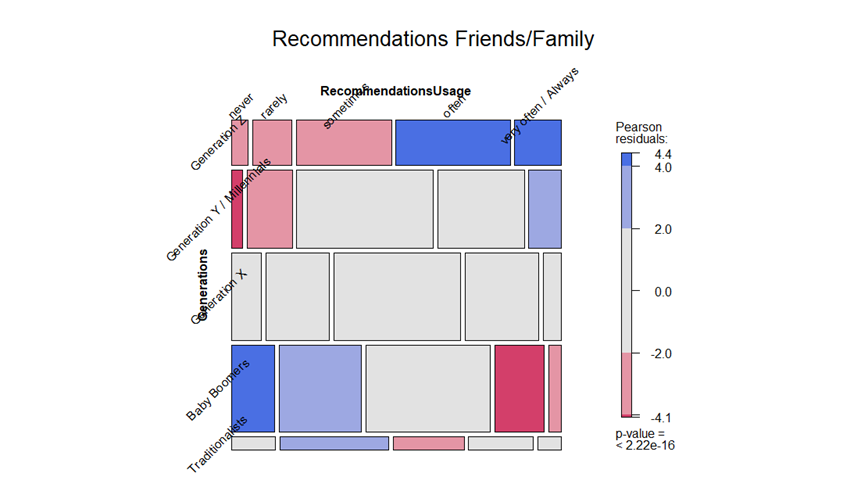  **Figure S6**. Recommendations from Friends/Family (F016b) | |
| 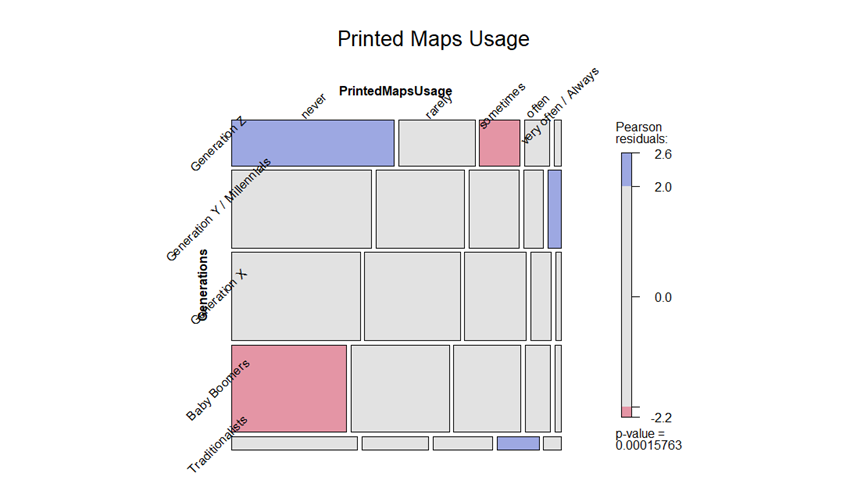 | 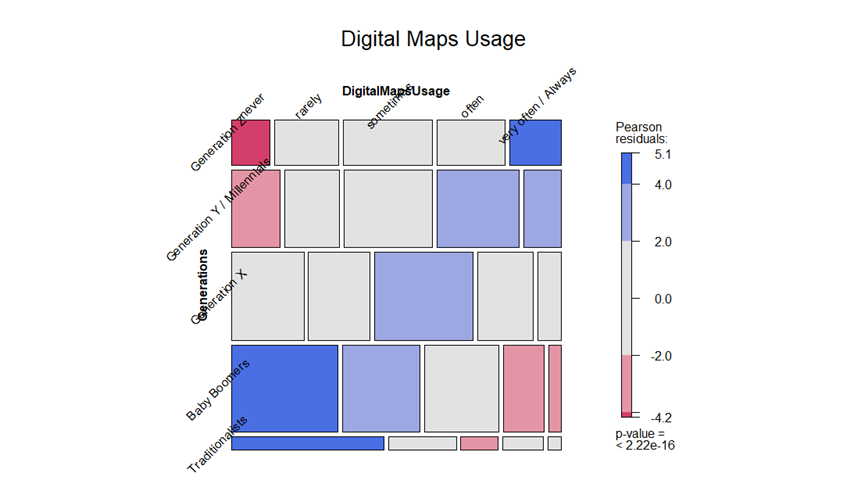 | |
| **Figure S7**. Printed Maps Usage (F016c)  *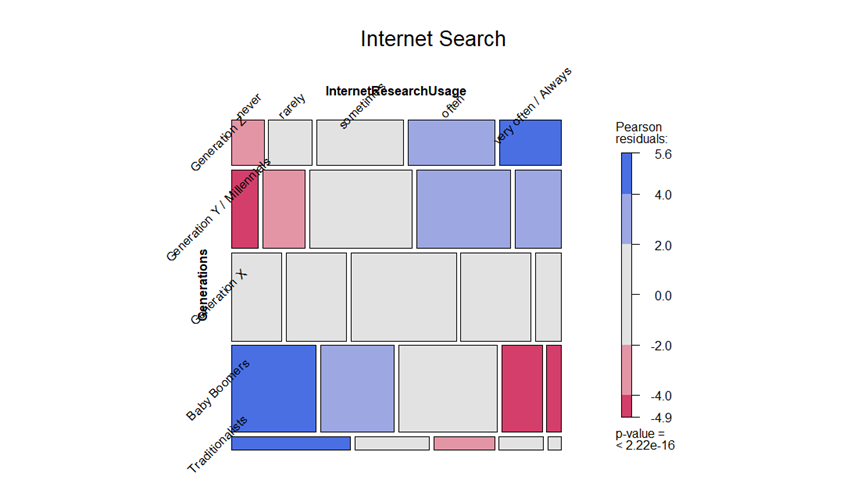* | **Figure S8**. Digital Maps Usage (F016d)  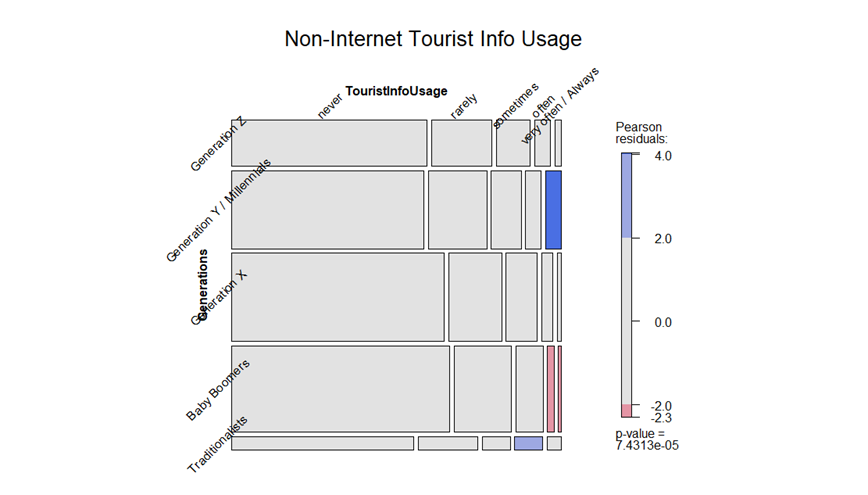 | |
| **Figure S9**. Internet Search (F016e) **Figure S10**. Non-Internet Tourist Info Usage (F016f) | | |
| 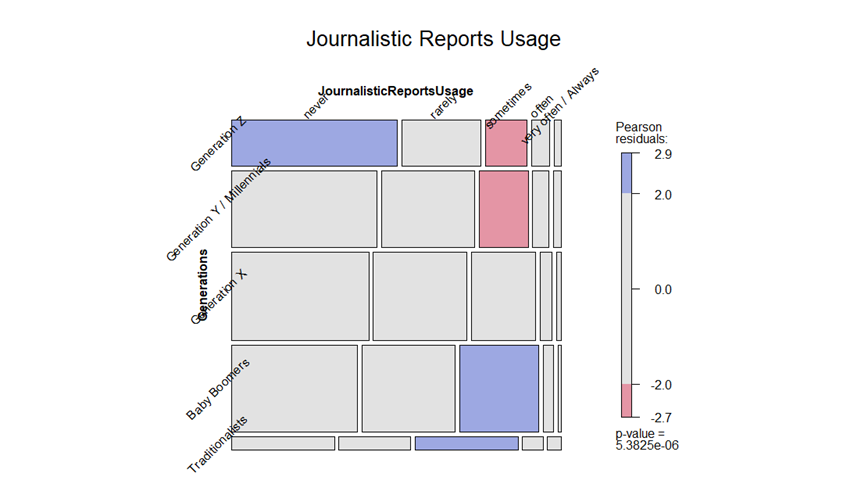 | | 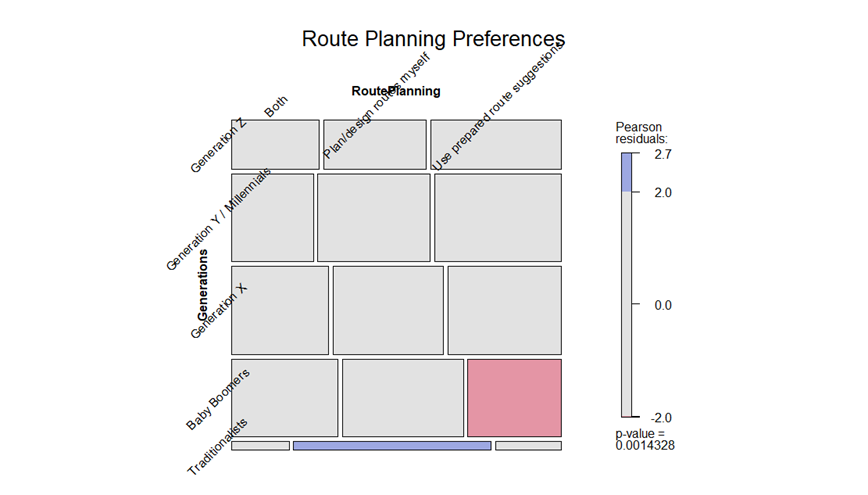 |
|  | |  |
| **Figure S11**. Journalistic Reports Usage (F016g) | | **Figure S12**. Route Planning Preferences (F028) |
| 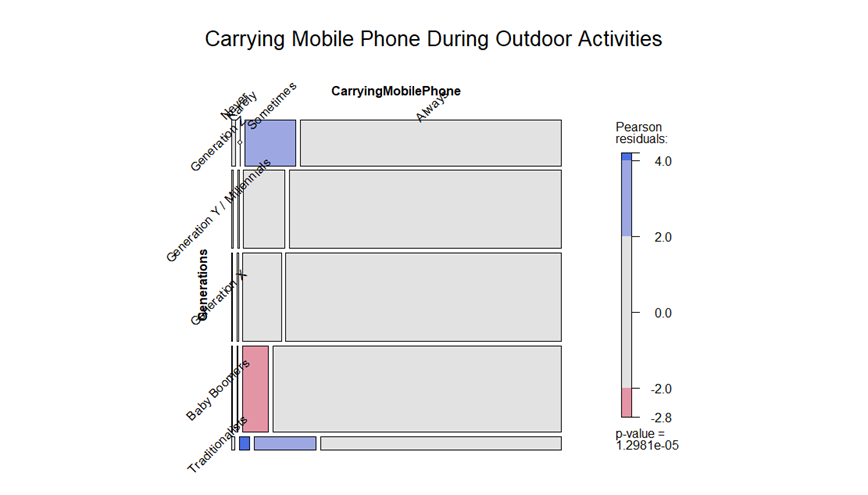  **Figure S13**. Carrying Mobile Phone During Outdoor Activities (F020) | | 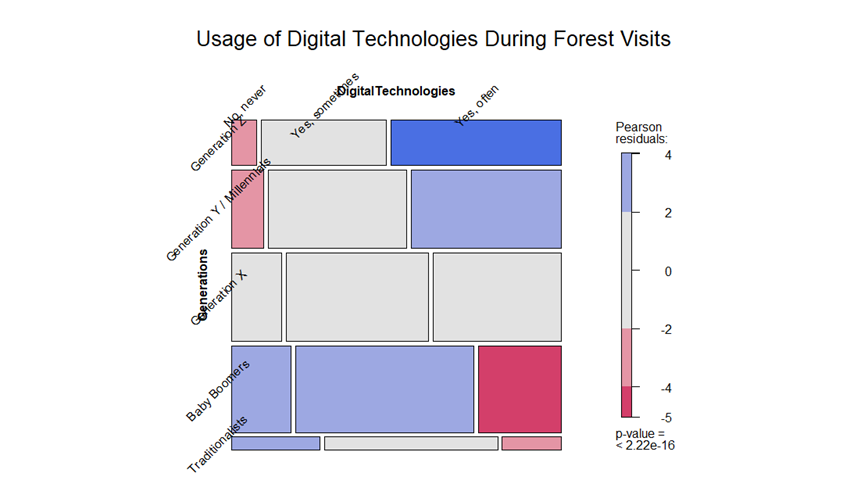  **Figure S14**. Usage of Digital Technologies During Forest Visits (F022) |
| **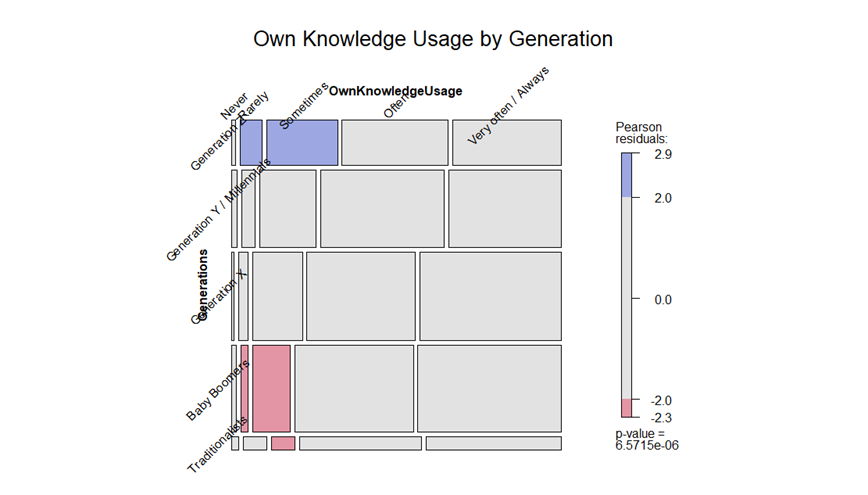**  **Figure S15**. Own Knowledge Usage by Generation (F019a)  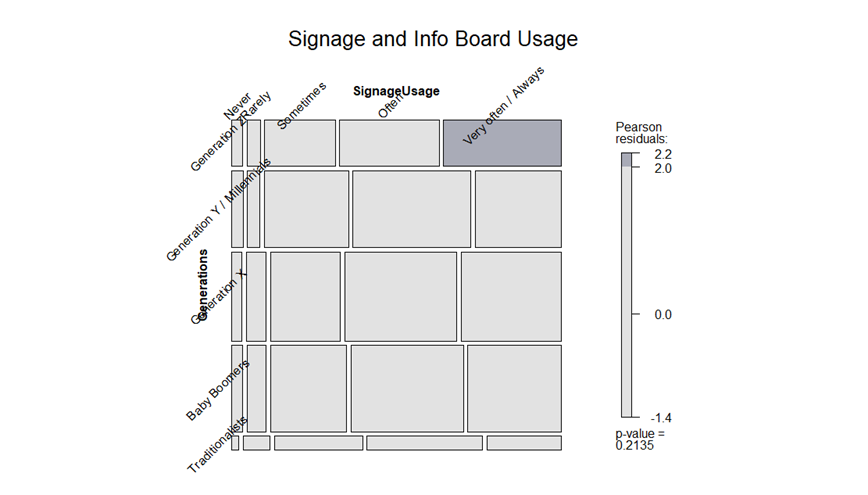  **Figure S17**: Signage and Info Board Usage (F019c) | | **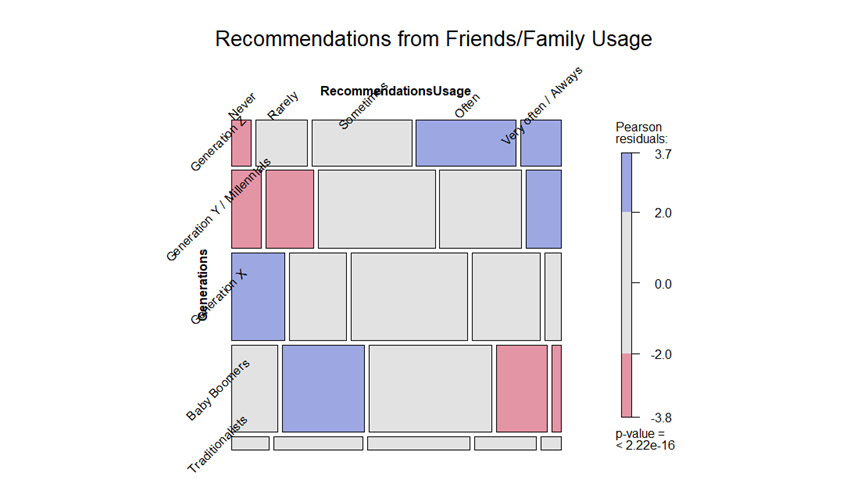Figure S16**. Recommendations from Friends/Family Usage (F019b)  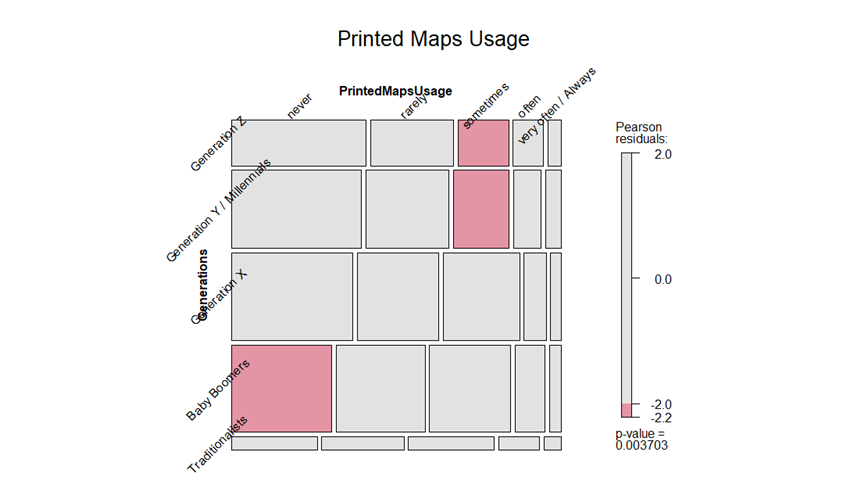  **Figure S18**: Printed Maps Usage (F019d) |
| 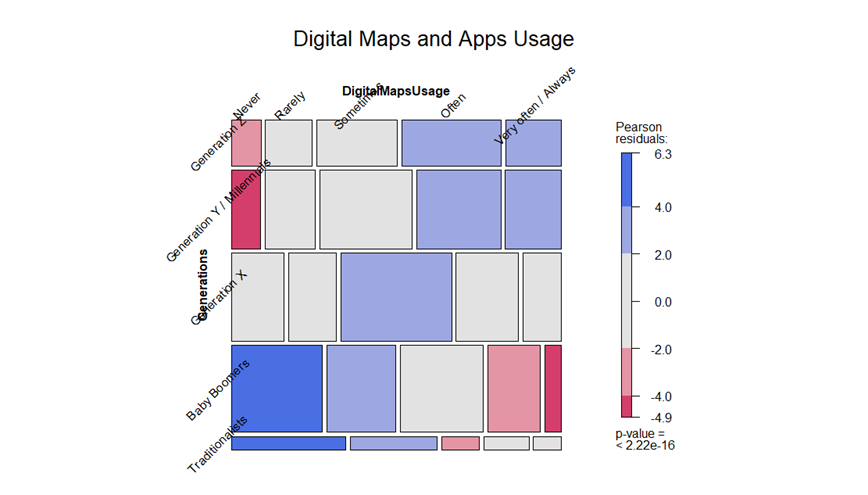 | | 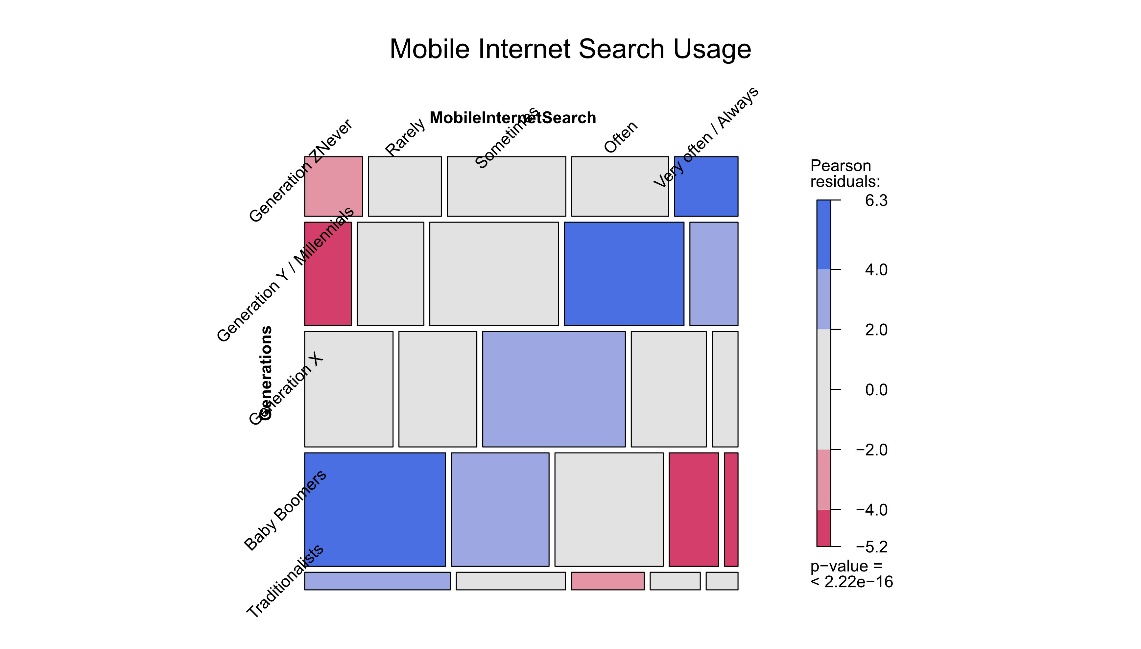 |
| **Figure S19**. Digital Maps and Apps Usage (F019e) | | **Figure S20**. Mobil Internet Search Usage (F019f) |
| 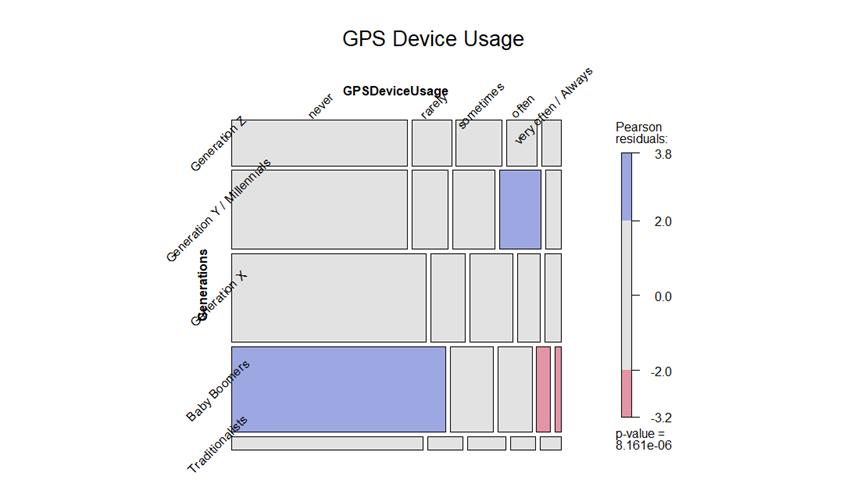 | | 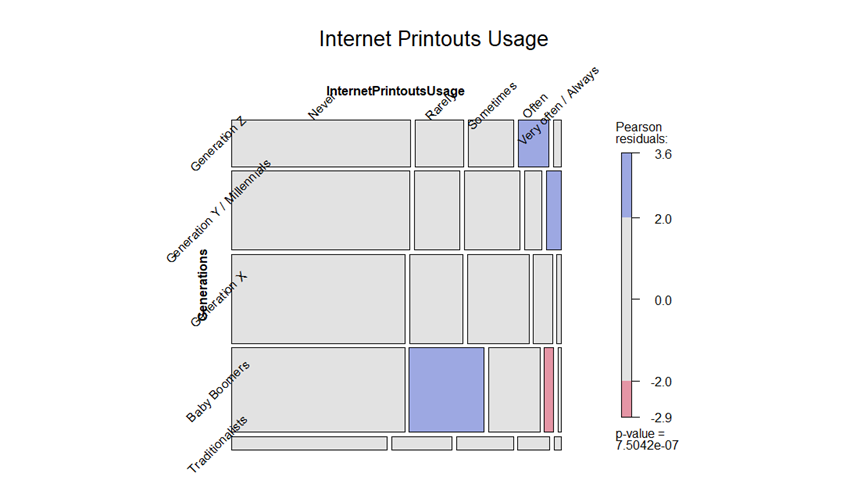 |
| **Figure S21**. GPS Device Usage (F019g)  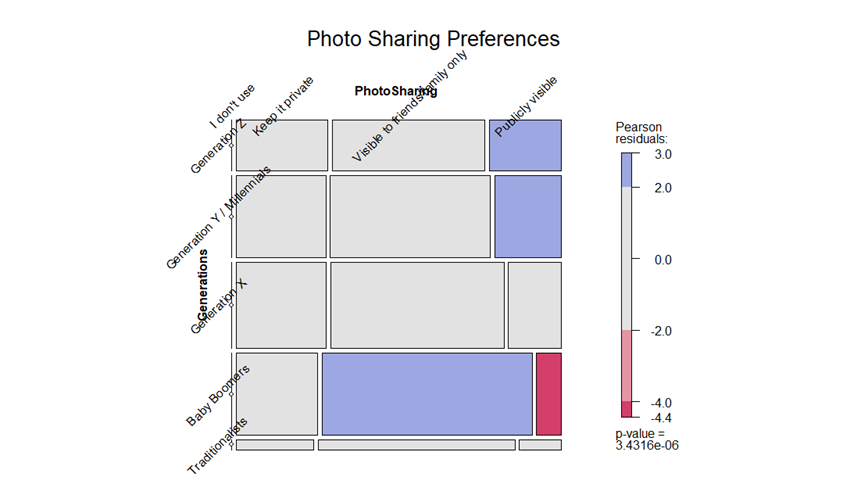  **Figure S23**. Photo Sharing Preferences (F030a)  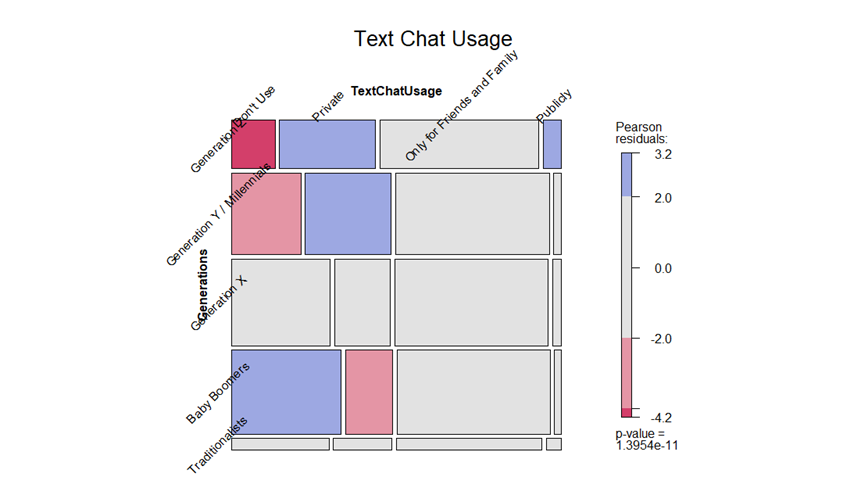**Figure S25**. Text Chat Usage (F030c)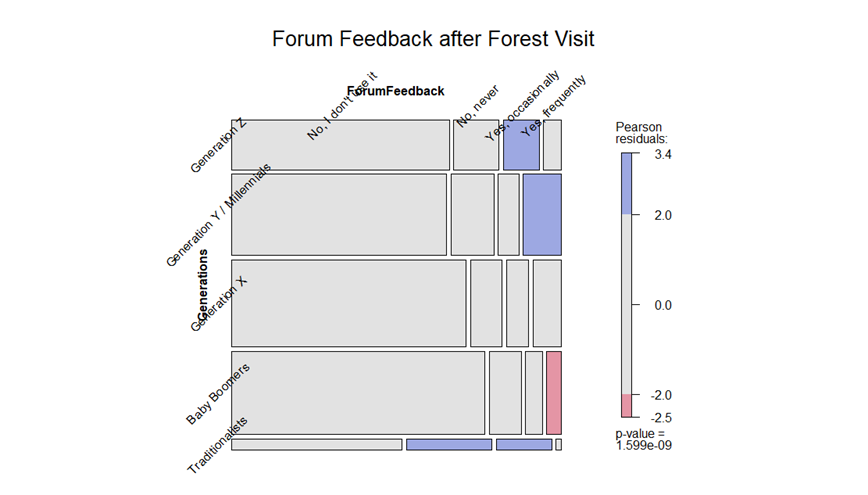  **Figure S27**. Forum Feedback after Forest Visit (F030e)  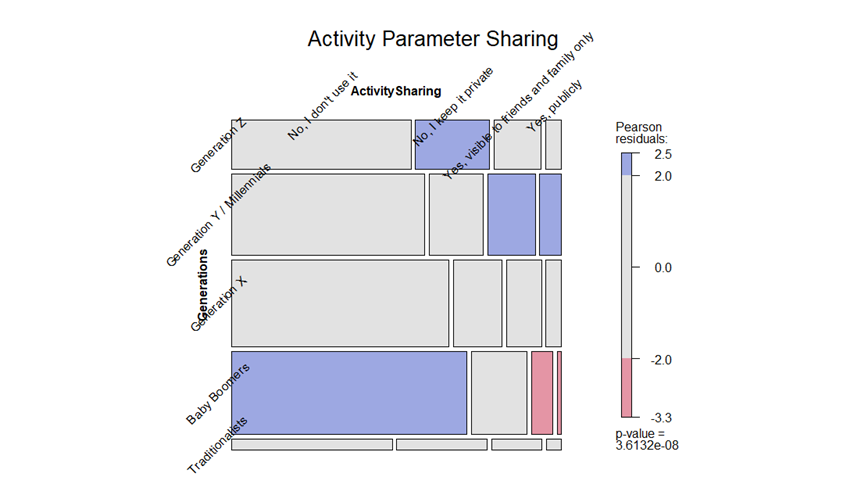  **Figure S29**. Activity Parameter Sharing (F030j)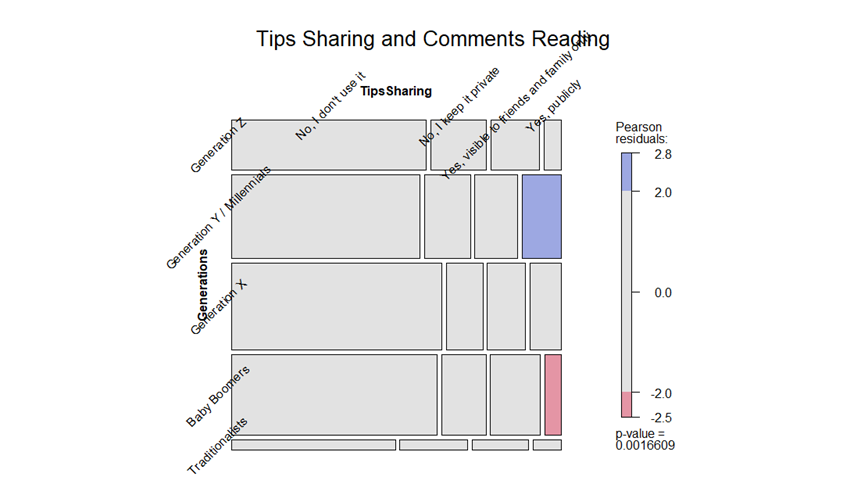  **Figure S31**. Tips Sharing and Comments Reading (F030l) | | \| **Figure S22**. Internet Printouts Usage (F019h) \| \| --- \| \| 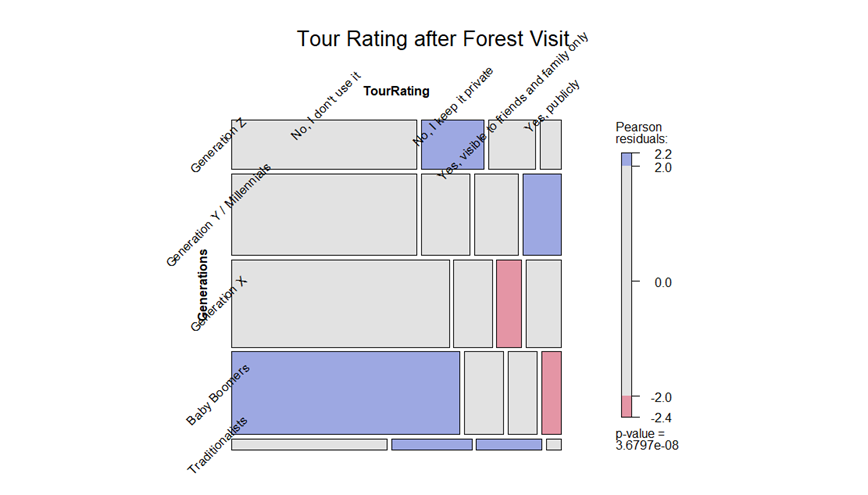  **Figure S24**. Tour Rating after Forest Visit (F030b) \| \| 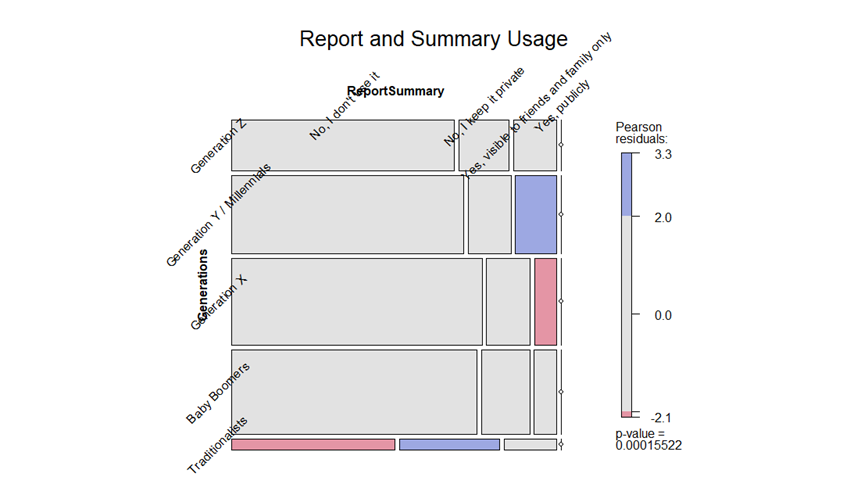 \| \| **Figure S26**. Report and Summary Usage (F030d) \| \| 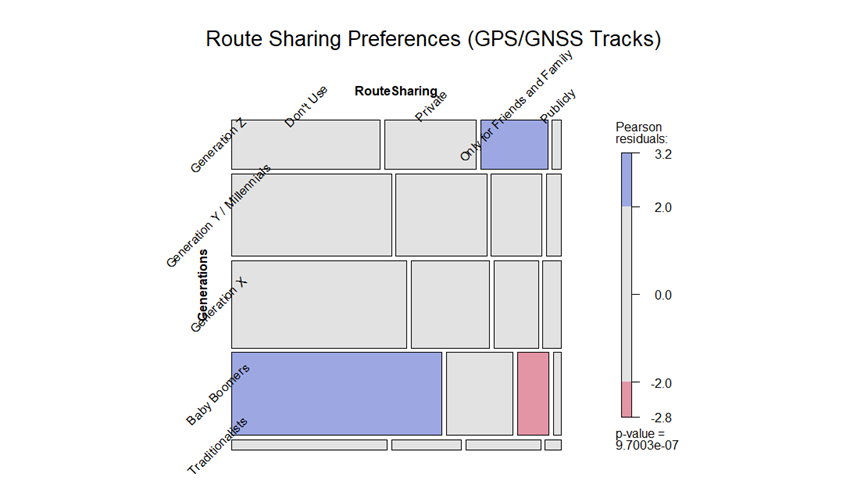  **Figure S28**. Route Sharing Preferences (GPS/GNSS Tracks) (F030b) \| \|  \| \| 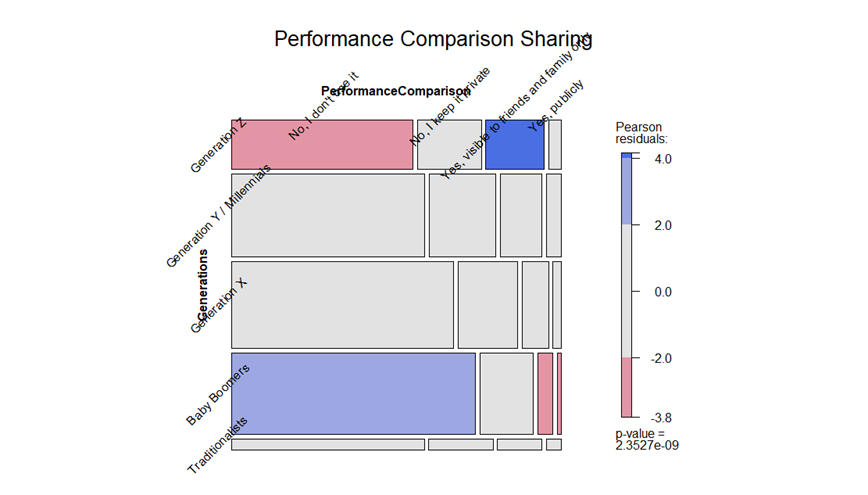 \| \| **Figure S30**. Performance Comparison Sharing (F030k) \| \|  \| \|  \| |
