# Supplementary material for: Understanding generational differences in digital skills and recreational behaviour for effective visitor management in forest destinations
Source: Sci Rep. 2025 May 23;15:17887. doi: 10.1038/s41598-025-02036-5 (PMC12098668; doi:10.1038/s41598-025-02036-5)
Supplement: Supplementary file 4 — Supplementary Material 4 [file 41598_2025_2036_MOESM4_ESM.docx]

**Supplementary Information (S4)**

***S4.1 Hot Spot Analysis of Forest Starting Points by Generations and Digital Skills***

**Figures S46-50**. Hot spot maps of the forest recreation starting points by generation: Generation Z (S46); Generation Y (S47); Generation X (S48); Baby Boomers (S49); Traditionalists (S50).


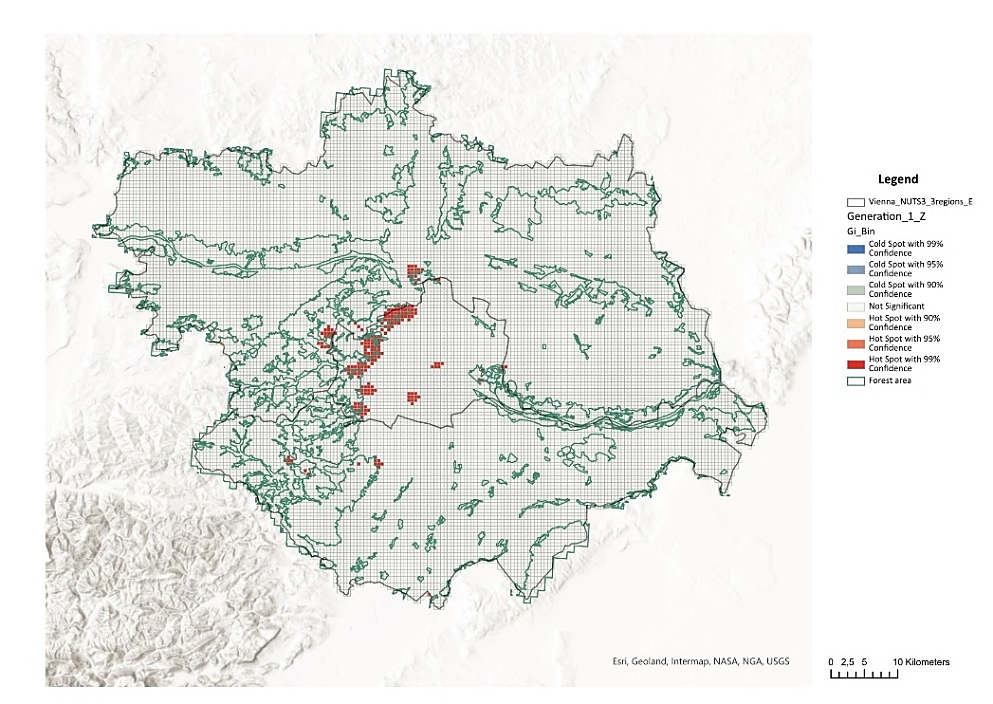

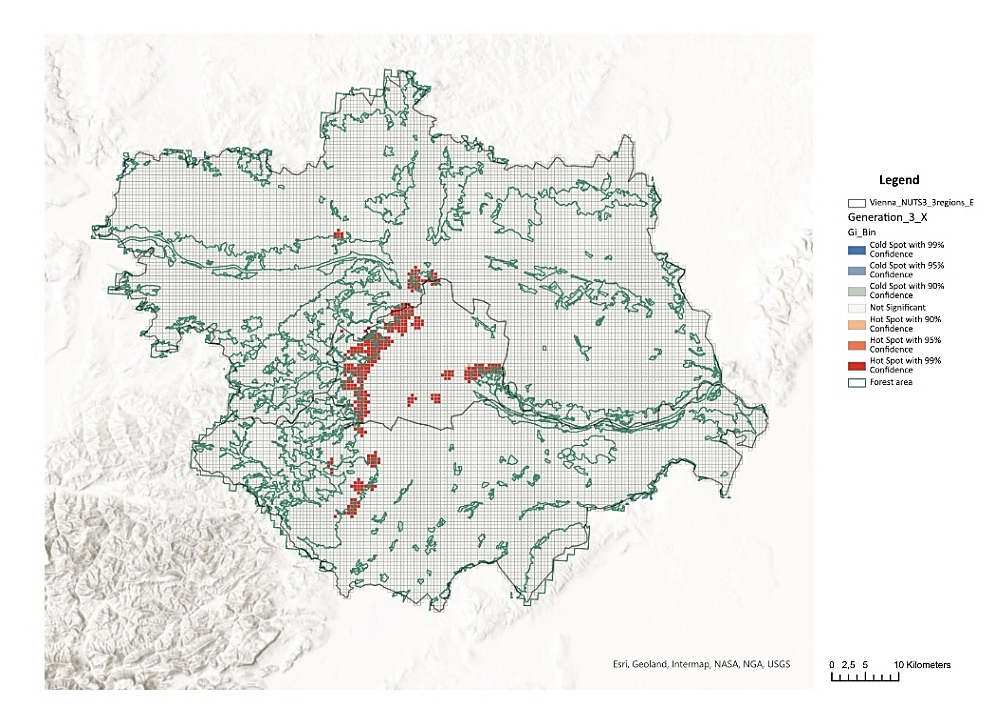

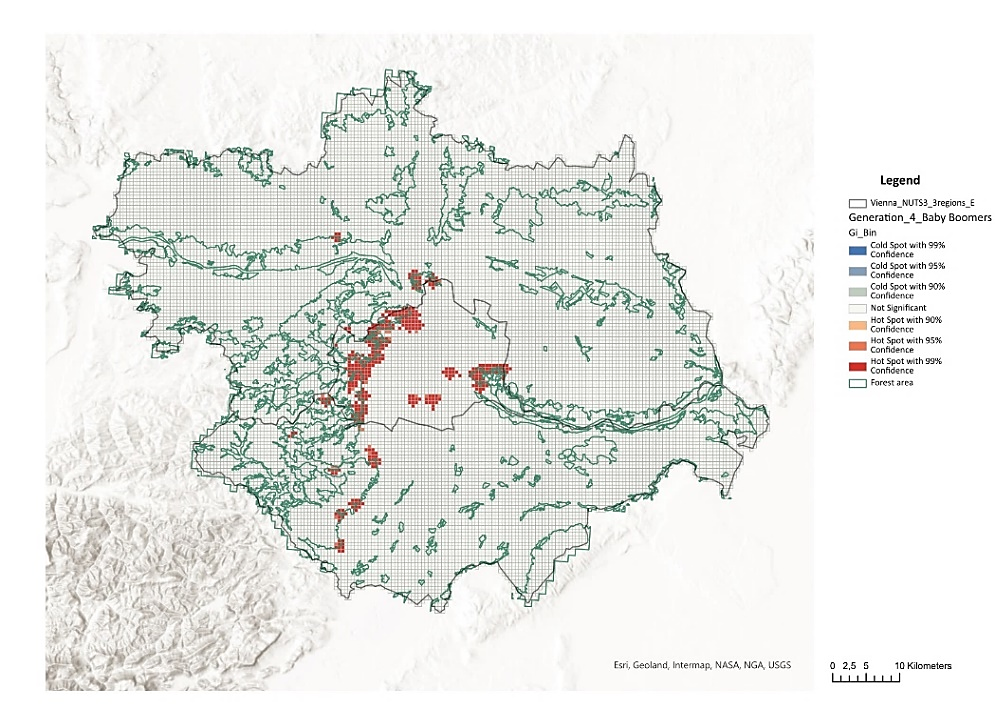

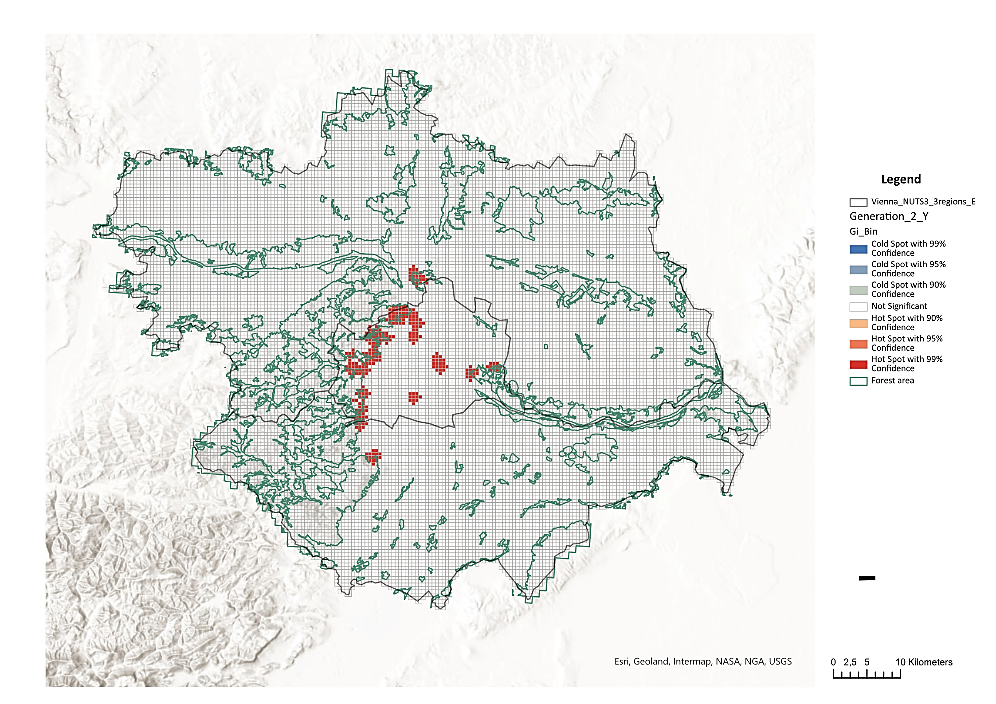

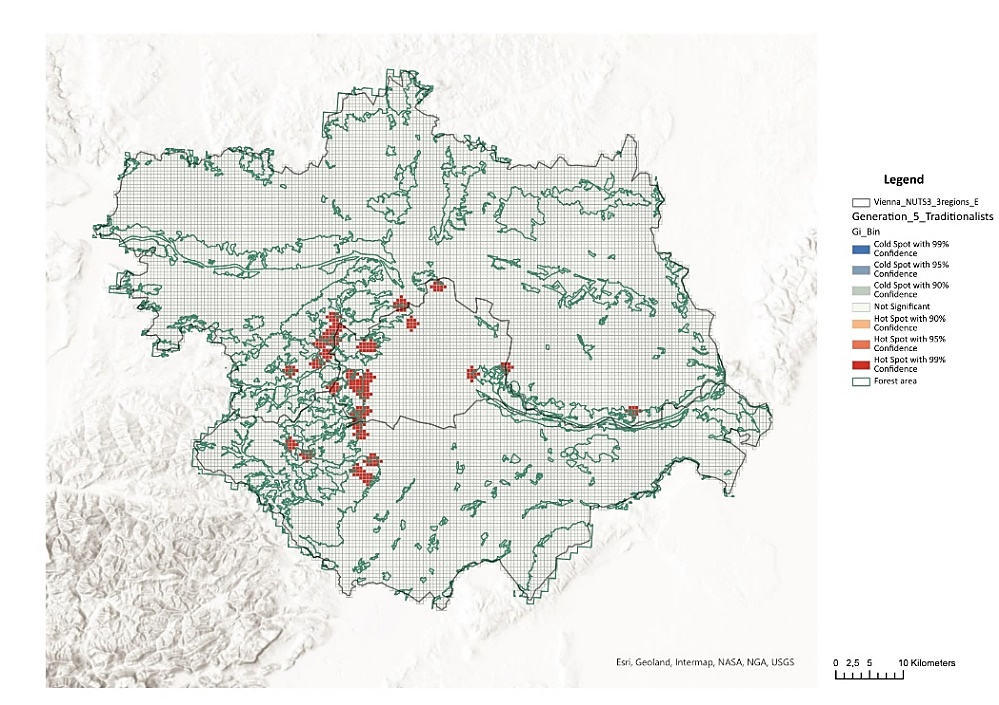

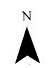

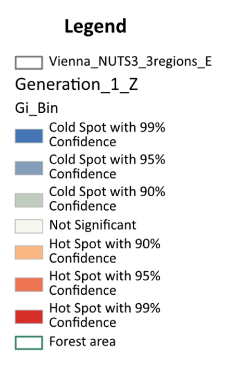

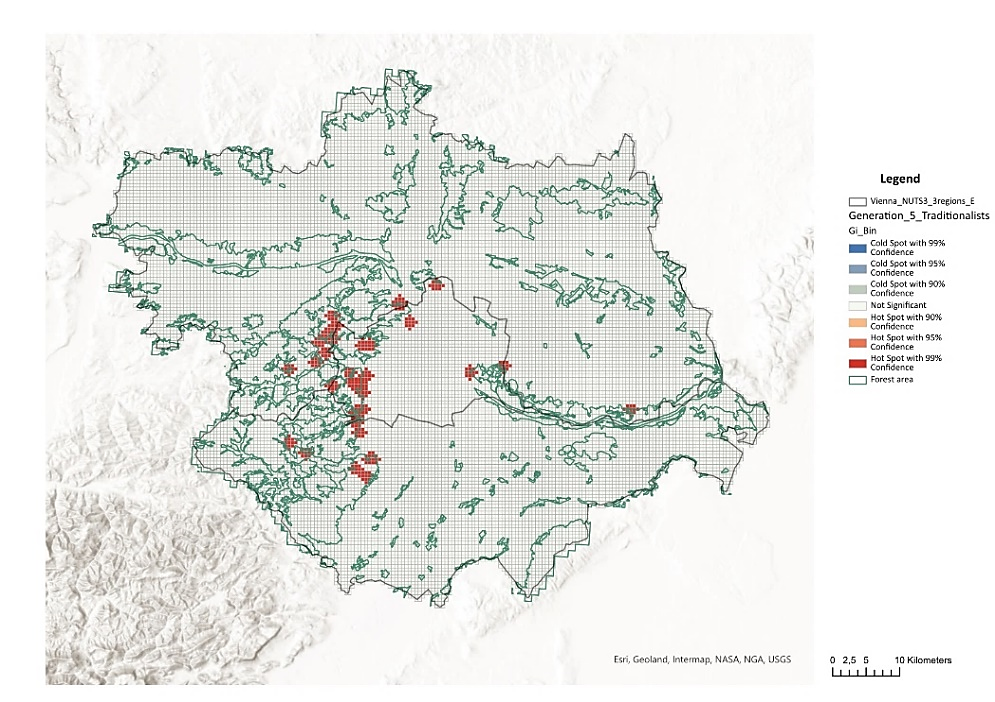


**Figure S48**: Generation X

**Figure S50**: Traditionalists

**Figure S49**: Baby Boomers

**Figure S47**: Generation Y

**Figure S46**: Generation Z

**Figure S37**: Traditionalists

| **Figures S51-54**: Hot spot maps of the forest recreation starting points by self-reported digital skill levels: Not at all (S51), Basic (S52), Advanced (S53), Very advanced (S54).  **Figure S54**: Digital Skill Level: Very Advanced 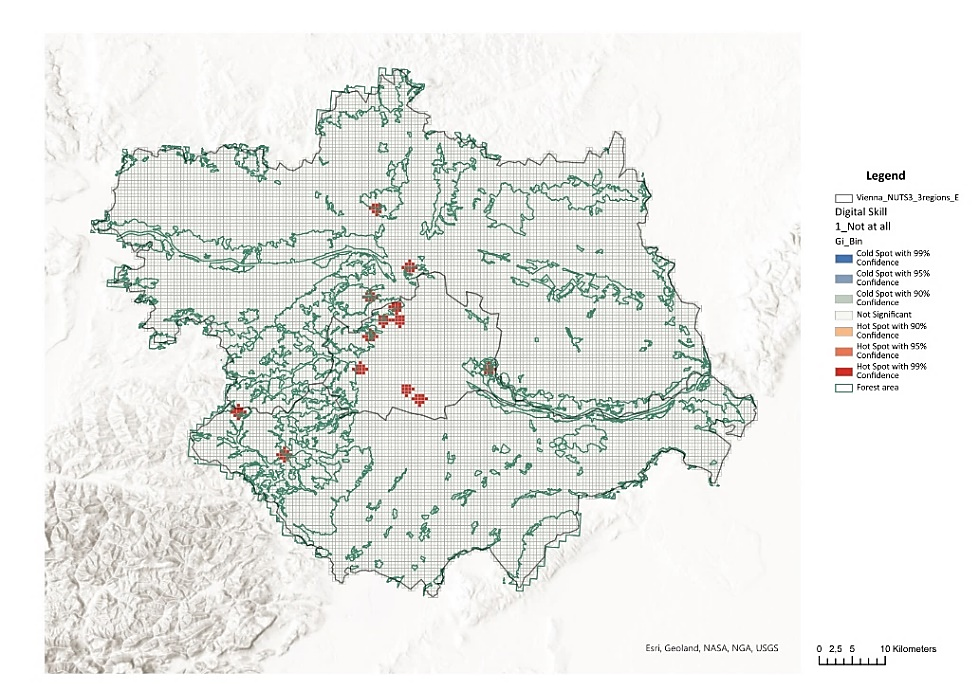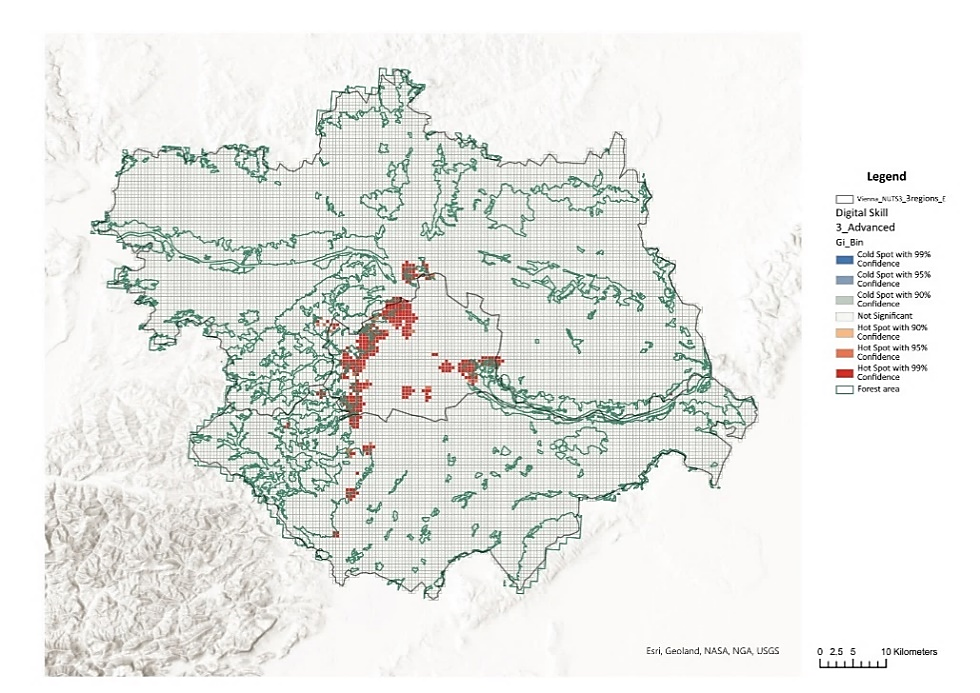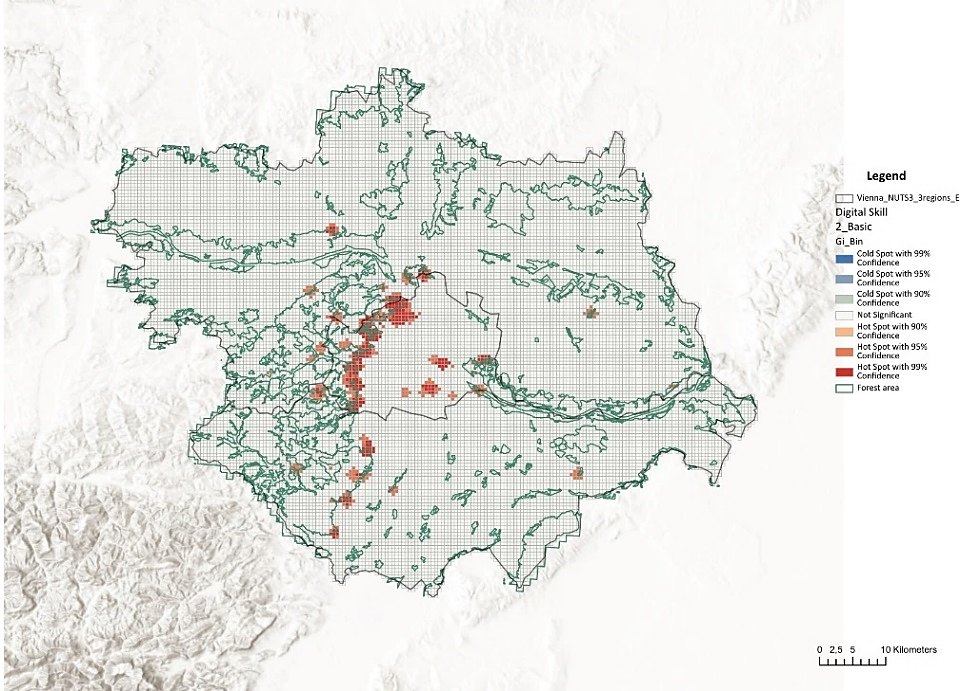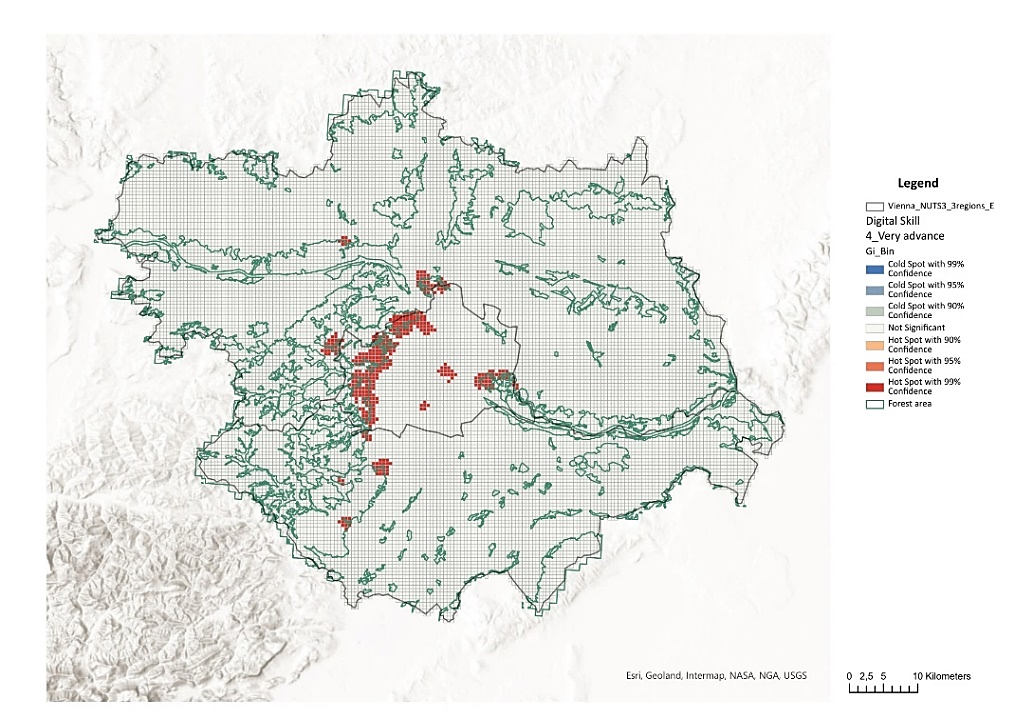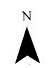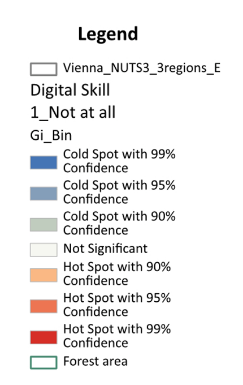 **Figure S53**: Digital Skill Level: Advanced  **Figure S52**: Digital Skill Level: Basic  **Figure S51**: Digital Skill Level: Not at all |
| --- |

The maps (Figures S46–S54) were created by the authors (Stefan and Taczanowska, 2024) using ArcGIS Pro (version 3.1.0, ESRI, Redlands, CA, USA; https://www.esri.com/arcgis) as part of this study. The hot spot analysis was conducted using the Getis-Ord Gi statistic with a fixed distance band of 500–1,000 metres to identify spatial clusters in forest visitation starting points (n = 1,116). They identify significant "hot spots" where visitation density is notably high. Figures S46–S50 show patterns by generation; Figures S51–S54 show patterns by digital skill level. Generational differences are evident in the spatial dispersion: younger groups (Gen Z, Y) show more spatially dispersed hot spots, while older groups (e.g. Baby Boomers, Traditionalists) tend to cluster around fewer, familiar locations. Higher digital skill levels were associated with more diverse and spatially distributed starting points.

**Table S5.** Validation statistics of hot spot clustering by generation. Summarises mean intensity, dispersion metrics, Z-scores, and p-values for hot spot detection across five generational groups. Z-scores above 1.96 indicate statistically significant clustering.

| Category | Mean | Std. Dev. | Skewness | Kurtosis | Sum | Max | Z-Score (avg) | P-Value (avg) |
| --- | --- | --- | --- | --- | --- | --- | --- | --- |
| Generation Z | 0,00653 | 0,09492 | 138,87 | 471,97 | 124 | 4 | 1,85 | 0,064 |
| Generation Y | 0,01342 | 0,18261 | 25,53 | 956,43 | 255 | 10 | 2,1 | 0,036 |
| Generation X | 0,01699 | 0,18799 | 20,21 | 615,31 | 323 | 9 | 2,35 | 0,019 |
| Baby Boomers | 0,01879 | 0,20829 | 22,5 | 845,97 | 357 | 12 | 2,5 | 0,012 |
| Traditionalists | 0,00232 | 0,06236 | 43,29 | 2672,73 | 44 | 5 | 1,6 | 0,109 |

**Table S6.** Validation statistics of hot spot clustering by digital skill level. Summarises spatial clustering metrics across self-assessed digital competence levels. The highest Z-scores were observed among “Advanced” and “Very Advanced” users, indicating greater spatial diversity in visit origins.

| \| Category \| Mean \| Std. Dev. \| Skewness \| Kurtosis \| Sum \| Max \| Z-Score (avg) \| P-Value (avg) \| \| --- \| --- \| --- \| --- \| --- \| --- \| --- \| --- \| --- \| \| Not at all \| 0,00203 \| 0,07845 \| 38,19 \| 1459,62 \| 39 \| 3 \| 1,7 \| 0,089 \| \| Basic \| 0,0121 \| 0,1517 \| 25,48 \| 1180,65 \| 230 \| 10 \| 2 \| 0,046 \| \| Advanced \| 0,02779 \| 0,28389 \| 20,2 \| 598,76 \| 528 \| 12 \| 2,8 \| 0,005 \| \| Very Advanced \| 0,01726 \| 0,20421 \| 22,06 \| 745,58 \| 328 \| 11 \| 2,4 \| 0,016 \| |
| --- | --- | --- | --- | --- | --- | --- | --- | --- | --- | --- | --- | --- | --- | --- | --- | --- | --- | --- | --- | --- | --- | --- | --- | --- | --- | --- | --- | --- | --- | --- | --- | --- | --- | --- | --- | --- | --- | --- | --- | --- | --- | --- | --- | --- | --- |

**S4.2 Statistical Analysis of Forest Visit Frequency, Generational Differences, and Digital Competence**

***S4.2.1 Forest Visit Frequency – Distribution and Transformation***

Self-reported annual forest visits (n = 2,521) ranged from 0 to 400, with a median of 7 and a mean of 23.5. The variable ‘annual_visits’ was computed as the sum of self-reported visits across the four seasons (spring, summer, autumn, winter), based on the survey items (F004) listed in Table S1. The distribution was strongly right-skewed (skewness = 4.16; kurtosis = 23.21). A Shapiro-Wilk test confirmed non-normality (**W = 0.4764, p < 2.2e-16**). To improve symmetry, a log transformation [log(1 + x)] was applied. It improved symmetry (skewness = 0.985), although normality was still not achieved (W = 0.953, p < 2.2e-16, suggesting that non-parametric methods remain appropriate for statistical testing.

All statistical analyses were conducted by the first author (Stefan) in R (v4.3.1), using the following packages: haven (for SPSS import), dplyr (data wrangling), ggplot2 and ggpubr (visualizations and Q-Q plots), moments (skewness & kurtosis), FSA (Dunn post-hoc test), and rstatix (Kruskal-Wallis post-hoc test).

**Table S7.** *Descriptive statistics of self-reported annual forest visits (before and after log transformation)*

| Statistic | Raw Values | Log-transformed values |
| --- | --- | --- |
| Minimum | 0.0 | 0.00 |
| 1st Quartile | 1.0 | 0.693 |
| Median | 7.0 | 2.08 |
| Mean | 23.5 | 2.94 |
| 3rd Quartile | 19.0 | 2.94 |
| Maximum | 400.0 | 6.00 |
| Skewness | 4.161 | 0.985 |
| Kurtosis | 23.211 | 3.21 |

***S4.2.2 Generational and Cluster Differences in Forest Visit Frequency***

A Kruskal-Wallis test revealed significant differences in log-transformed annual visits across the five generational groups (**χ² = 17.48, df = 4, p = 0.0016**). A separate Kruskal-Wallis test assessed differences across the four assigned visitor clusters, revealing highly significant differences (**χ² = 209.89, df = 3, <2.2e-16**). This suggests that distinct visitor types engage with forests at varying frequencies.

***Table S8.*** *Kruskal-Wallis Test results*

| Variable | Chi-Square (χ²) | df | p-value |
| --- | --- | --- | --- |
| Generations | 17.475 | 4 | 0.001563 |
| Clusters | 209.89 | 3 | <2.2e-16 |

***S4.2.3 Differences in Annual Visits by Digital Competence***

A **Kruskal-Wallis test** was also conducted to analyse differences in annual visits based on digital competence levels. Differences in visit frequency by digital skill levels were also significant (**χ² = 18.63, df = 4, p = 0.0009**). **Dunn’s post-hoc test** (Bonferroni-adjusted) indicated that individuals with “Advanced” digital skills visited forests significantly more often than those with “Basic” skills (**p = 0.0044**). Other pairwise comparisons were not statistically significant.

A **Spearman correlation** test found a weak but statistically significant positive correlation between digital competence and annual forest visits (**ρ = 0.066, p = 0.0009**), indicating that digital competence is marginally associated with more frequent forest visitation. These results suggest that while digital competence might influence forest visitation behaviour, other factors likely play a larger role.

***Table S9.*** *Dunn’s post-hoc comparisons by digital skill. Note: Full Dunn’s test results are provided in Table S10.*

| Comparison | Z | p-value (Adj) |
| --- | --- | --- |
| Advanced - Basic | 2.85 | 0.0443 |
| Basic - Highly Advanced | -2.73 | 0.0625 (borderline) |
| Other comparisons | — | > 0.05 (n.s.) |

***Table S10.*** *Dunn’s post-hoc comparisons by digital skill, full pairwise test output (Z-scores, adjusted p-values). Dunn's Test for Pairwise Comparisons suggests that "Advanced" users visit significantly more often than "Basic" users.*

| Comparison | Z | p-value (Unadj) | p-value (Adj) |
| --- | --- | --- | --- |
| Advanced - Basic | 2.85 | 0.044 | 0.0443 |
| Advanced - Highly Advanced | -0.14 | 0.8843 | 1.0 |
| Basic - Highly Advanced | -2.73 | 0.0062 | 0.0625 (borderline) |
| Advanced - No Answer | 1.69 | 0.0893 | 0.8931 |
| Basic - No Answer | 0.85 | 0.3928 | 1.0 |
| Highly Advanced - No Answer | 1.72 | 0.0845 | 0.8454 |
| Advanced - None | 2.75 | 0.0059 | 0.0586 |
| Basic - None | 1.89 | 0.0578 | 0.5784 |
| Highly Advanced - None | 2.77 | 0.0055 | 0.0556 |
| No Answer - None | 0.75 | 0.4483 | 1.0 |

***S4.2.4 Conclusion***

*Forest visitation frequency varied significantly across* ***generations****,* ***digital visitor clusters****, and* ***digital competence levels****. Although digital skills showed a statistically significant effect, its impact was relatively small, implying that other factors, such as lifestyle, accessibility, or motivation, may play a more influential role. Given the skewed nature of the data,* ***non-parametric testing*** *was appropriate throughout.*
